# Distinct USP25 and USP28 Oligomerization States Regulate Deubiquitinating Activity

**DOI:** 10.1016/j.molcel.2019.02.030

**Published:** 2019-05-02

**Authors:** Malte Gersch, Jane L. Wagstaff, Angela V. Toms, Bradford Graves, Stefan M.V. Freund, David Komander

**Affiliations:** 1 Medical Research Council Laboratory of Molecular Biology, Francis Crick Avenue, Cambridge CB2 0QH, UK; 2 Chemical Genomics Centre, Max-Planck-Institute of Molecular Physiology, Otto-Hahn-Str. 11, 44227 Dortmund, Germany; 3 Department of Chemistry and Chemical Biology, Technical University Dortmund, Otto-Hahn-Str. 4a, 44227 Dortmund, Germany; 4 FORMA Therapeutics, Arsenal Street, Watertown, MA 02472, USA; 5 Ubiquitin Signalling Division, The Walter and Eliza Hall Institute of Medical Research, 1G Royal Parade, Parkville, VIC 3052, Australia; 6 Department of Medical Biology, The University of Melbourne, Melbourne, VIC 3010, Australia

**Keywords:** ubiquitin, deubiquitylating enzyme, ubiquitin specific protease, c-MYC, TRAF

## Abstract

The evolutionarily related deubiquitinating enzymes (DUBs) USP25 and USP28 comprise an identical overall domain architecture but are functionally non-redundant: USP28 stabilizes c-MYC and other nuclear proteins, and USP25 regulates inflammatory TRAF signaling. We here compare molecular features of USP25 and USP28. Active enzymes form distinctively shaped dimers, with a dimerizing insertion spatially separating independently active catalytic domains. In USP25, but not USP28, two dimers can form an autoinhibited tetramer, where a USP25-specific, conserved insertion sequence blocks ubiquitin binding. In full-length enzymes, a C-terminal domain with a previously unknown fold has no impact on oligomerization, but N-terminal regions affect the dimer-tetramer equilibrium *in vitro*. We confirm oligomeric states of USP25 and USP28 in cells and show that modulating oligomerization affects substrate stabilization in accordance with *in vitro* activity data. Our work highlights how regions outside of the catalytic domain enable a conceptually intriguing interplay of DUB oligomerization and activity.

## Highlights

• Insertions in USP25 and USP28 catalytic domains mediate their oligomerization
• DUB activity of USP25, but not USP28, is inhibited by tetramerization
• An autoinhibitory motif in USP25 blocks Ub binding, impairing its activity
• Dimers of USP28, as well as dimers and tetramers of USP25, exist in cells

## Graphical Abstract

**Figure:**
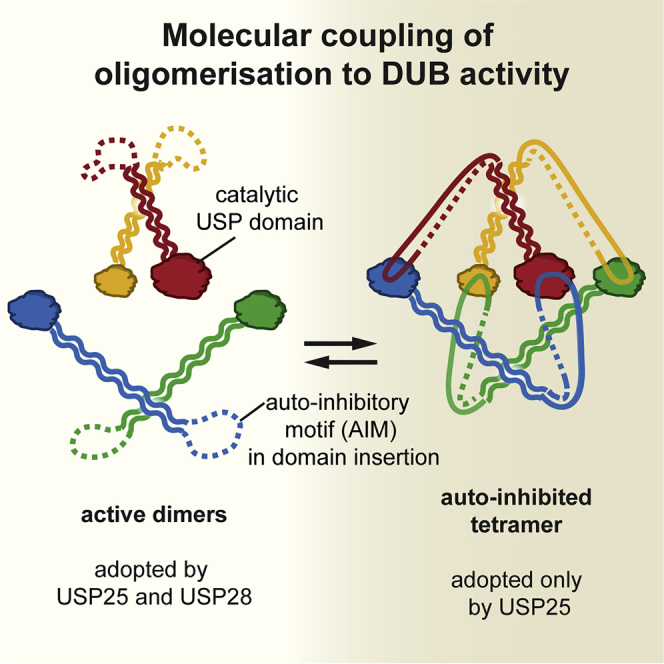

## Teaser

Deubiquitinating enzymes restrict the degree of ubiquitin-mediated signaling in cells, and their activity must therefore be tightly regulated. In this manuscript, Gersch et al. show how the catalytic activity of USP25, but not of its closely related paralog USP28, is regulated by oligomerization through sequences inserted into their catalytic domains.

## Introduction

The complement of human deubiquitinases (DUBs) comprises ∼100 enzymes, including more than 50 ubiquitin-specific proteases (USPs) (Clague et al., 2013, Mevissen and Komander, 2017). USPs are precision tools that bind and deubiquitinate their substrates, often leading to substrate stabilization. What is less clear is the degree of redundancy in the system, particularly for subsets of highly similar USPs that most likely arose from gene duplication events (Figure S1A). Biological roles of DUBs are currently emerging (Clague et al., 2013), and the available data suggest non-redundant roles even for highly similar enzymes.

A striking case is the DUB pair USP28 and USP25 (Figures S1B and S1C). USP28 has been identified as a regulator of the DNA damage response (DDR) (Zhang et al., 2006) and of transcription via stabilization of c-MYC (Popov et al., 2007), which has led to intense study. USP28 adopts a mostly nuclear localization, and reported substrates include LSD1 involved in chromatin and DNA methylation (Wu et al., 2013) and 53BP1 involved in DDR and cell cycle regulation (Cuella-Martin et al., 2016). Several functions of USP28 have been linked to cullin-SCF E3 ligase biology, and genetic analysis suggested a connection between USP28, FBW7, and substrate levels (Cremona et al., 2016). Pathophysiologically, USP28 drives colorectal and non-small-cell lung cancer (Diefenbacher et al., 2014, Wang et al., 2018), which has made it a sought-after pharmacological target (Harrigan et al., 2018).

In contrast, USP25 has been linked to various cytosolic roles (Figure S1C), mostly involving inflammatory processes. Viruses and cytokines trigger ubiquitin-dependent activation of nuclear factor κB (NF-κB) and cell death, and these processes are under tight control by deubiquitinases (Harhaj and Dixit, 2012). USP25 has been genetically shown to negatively regulate TRAF3 and TRAF6 signaling downstream of Toll-like receptor 4 (TLR4) (Lin et al., 2015, Zhong et al., 2013) and TRAF3 and TRAF6 signaling downstream of the interleukin-17 (IL-17) receptor (Zhong et al., 2012). USP25 also binds tankyrases, linking it to Wnt signaling (Xu et al., 2017). Overall, USP25 and USP28 are among the biologically well-studied DUBs, yet how they regulate such diverse cellular processes is largely unknown.

USP25 and USP28 share an identical domain structure and highly homologous catalytic domains (Figure S1B). An N-terminal region incorporates a number of ubiquitin-binding domains (ubiquitin-associated [UBA] and 2 ubiquitin-interaction motifs [UIMs]) as well as a SUMO2/3-selective SUMO-interaction motif (SIM) that drives SUMOylation of a neighboring Lys residue. Modification with SUMO prevents UIM-mediated ubiquitin interactions required for efficient ubiquitin chain cleavage (Meulmeester et al., 2008), which seems to be shared by USP28 (Zhen et al., 2014). NMR analysis showed that the N-terminal domains are embedded within a flexible sequence context (Yang et al., 2017).

**Figure 1:**
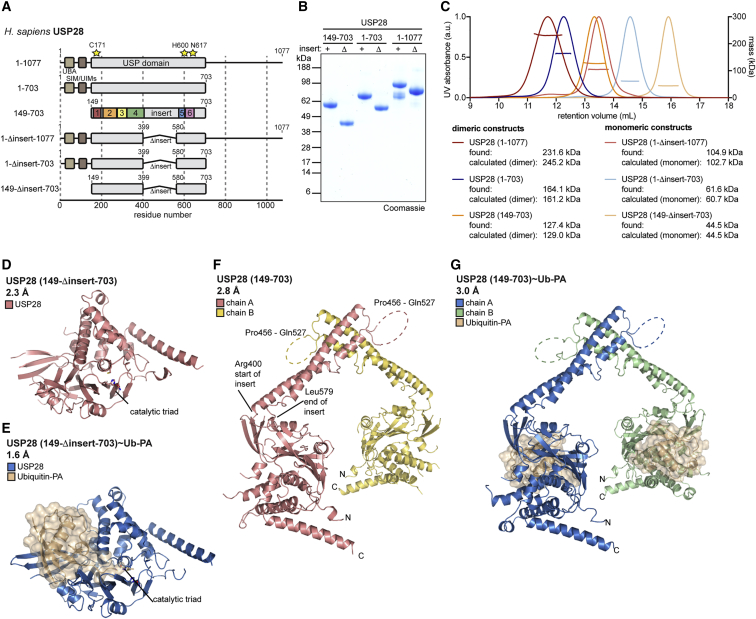
An Insertion in the Catalytic Domain of USP28 Mediates Its Dimerization (A) Schematic representation of human USP28 constructs. The catalytic domain is shown in gray; an N-terminal UBA domain, SUMO-interaction motif (SIM), and ubiquitin-interaction motifs (UIMs) are shown in brown. Residues of the catalytic triad are shown as yellow stars. Colored boxes refer to USP box annotation (Ye et al., 2009). (B) Coomassie-stained SDS-PAGE gel of indicated recombinant proteins. (C) SEC-MALS analysis of proteins shown in (B). Identified masses are matched to either monomeric (light colors) or dimeric (dark colors) expected masses. (D and E) Crystal structures of catalytic domains of USP28 with the insertion deleted in the apo form (D) or bound to ubiquitin-propargylamine (Ub-PA; transparent surface) (E). Catalytic triad residues are indicated. (F and G) Crystal structures of the catalytic domain of human USP28 in its apo form (F) or bound to Ub-PA (G). N and C termini, insertion boundaries, and disordered residues are indicated.

Less is known about the remaining 85% of the enzymes. USP catalytic domains are usually around 350 amino acids (aa) in length; however, in USP25/28, the catalytic domains span ∼550 aa due to a large, conserved insertion of unknown function at a common insertion point between USP boxes 4 and 5 (Figure 1A; Ye et al., 2009). The catalytic domains are followed by an unannotated region of ∼340 aa of unknown structure; several reports suggest roles in substrate binding for the C-terminal part (Xu et al., 2017).

We here characterize USP25 and USP28 at the molecular level and report crystal structures of the catalytic domains with and without insertions, and with and without ubiquitin, as well as a structure of the USP25 C-terminal domain. We validate the structures on a biochemical level and in cells on structural and functional levels to explain the molecular distinction of USP25 and USP28. Our data reveal a further paradigm for USP regulation, showing how similar domain architectures were adopted to exist in cells in active and autoinhibited conformations, in this case by virtue of oligomerization via USP-specific insertions.

## Results

### Biochemical Characterization of USP28

Human USP28 comprises 1,077 aa, of which the N-terminal 150 have been structurally characterized (Zhen et al., 2014). To gain insights into the catalytic domain architecture, we designed constructs with boundaries according to previous studies and secondary structure predictions (Zhen et al., 2014). Box annotation was used to identify and remove a 180-aa insertion within the catalytic domain to aid structural biology (Gersch et al., 2017, Ye et al., 2009; Figure 1A). Proteins were expressed and purified from *E. coli*, yielding homogeneous material for most constructs (Figure 1B). Unusual running behavior on gel filtration prompted more detailed size-exclusion chromatography multi-angle light scattering (SEC-MALS) analysis, revealing that proteins lacking the insertion were monomeric, and USP28 variants including the insertion were dimeric (Figure 1C). This was unchanged when catalytic domains were modified with the ubiquitin-based reactive probe ubiquitin-propargylamine (Ub-PA) (Ekkebus et al., 2013; Figure S1D). Intriguingly, at the concentrations tested, there were no apparent monomer-dimer equilibria in solution, suggesting that the insertion is necessary and sufficient to establish a tight dimerization (Figures 1C and S1D). This was further confirmed by SEC-MALS experiments using fluorescently labeled USP28 catalytic domain with Ub-PA bound: exclusively dimeric behavior was observed at concentrations below 1 nM (Figure S1E).

We further characterized DUB activity by assessing cleavage of a fluorescent Ub-KG-TAMRA substrate by USP28 variants (Figures S1F and S1G). All constructs including the insertion displayed near-identical catalytic efficiency, suggesting that N- and C-terminal regions do not interfere with a minimal ubiquitin cleavage reaction. Interestingly, deletion of the insertion decreased catalytic efficiency to ∼50%, similarly for all constructs (Figure S1G).

### Structural Analysis of USP28

To explain the oligomeric behavior and activity differences, we initiated structural studies. A variety of crystal structures of USP28 were determined for constructs spanning the catalytic domain (aa 149–703; Figures 1D–1G and S1H–S1K; Table 1). Crystals of USP28 without the insertion diffracted to 2.3-Å resolution (Figure 1D). Crystals of the same protein construct modified with Ub-PA diffracted to 1.6 Å (Figure 1E), and the structures were determined by molecular replacement using the previously published structure of USP7 (Hu et al., 2002) as a search model. Both structures revealed a canonical catalytic domain with high similarity to previous USP structures with and without ubiquitin; DALI analysis showed the highest similarity to USP7 (PDB: 1NBF; root-mean-square deviation [RMSD] ∼2.0 Å; Z ∼31.5; Figure S1L). Unlike USP7, apo USP28 showed an aligned catalytic triad (Hu et al., 2002).

**Table 1:** Data Collection and Refinement Statistics

	USP28 (149-Δinsert-703) (PDB: 6HEH)	USP28 (149-Δinsert-703)∼Ub-PA (PDB: 6HEI)	USP28 (149-703) (PDB: 6HEJ)	USP28 (149-703)∼Ub-PA (PDB: 6HEK)	USP25 (157-714) (PDB: 6HEL)	USP25 (748-1048) (PDB: 6HEM)
**Data Collection**						

Beamline	ESRF ID30B	Diamond I03	ESRF ID30B	Diamond I04-1	ESRF ID29	Diamond I02
Wavelength (Å)	0.9686	0.9762	0.9763	0.9282	0.9790	0.9795
Space group	*I*4_1_32	*P*22_1_2_1_	*I*222	*I*222	*I*4	*P*2_1_2_1_2_1_
*a*, *b*, *c* (Å)	189.15, 189.15, 189.15	49.51, 86.41, 97.85	104.24, 200.46, 206.06	103.21, 199.79, 204.90	139.18, 139.18, 190.47	55.96, 78.29, 84.91
*α*, *β*, *γ* (°)	90, 90, 90	90, 90, 90	90, 90, 90	90, 90, 90	90, 90, 90	90, 90, 90
Anisotropy correction	–	–	yes	yes	yes	–
Total reflections	233,139 (20,128)	210,197 (20,685)	163,141 (7,235)	206,967 (18,758)	111,871 (6,207)	184,886 (17,717)
Unique reflections	27,219 (2,698)	52,145 (5,114)	31,781 (1,590)	30,634 (2,785)	20,988 (1,050)	40,292 (3,983)
Resolution (Å)	50.55–2.26 (2.34–2.26)	49.51–1.64 (1.70–1.64)	143.68–2.79 (3.03–2.79)	143.05–3.03 (3.36–3.03)	59.17–2.94 (3.39–2.94)	57.56–1.72 (1.78–1.72)
Ellipsoidal resolution limits (Å) [direction]	–	–	4.48 [a^∗^]	3.81 [a^∗^]	3.86 [a^∗^]	–
			2.79 [b^∗^]	3.15 [b^∗^]	3.86 [b^∗^]	
			2.80 [c^∗^]	3.02 [c^∗^]	2.94 [c^∗^]	
*R*_*merge*_	0.065 (0.699)	0.049 (0.637)	0.053 (1.03)	0.100 (1.24)	0.052 (1.43)	0.071 (0.751)
*R*_*meas*_	0.068 (0.752)	0.057 (0.733)	0.059 (1.16)	0.109 (1.35)	0.058 (1.57)	0.080 (0.852)
*I/σ(I)*	19.8 (2.7)	13.5 (2.3)	15.6 (1.5)	14.2 (1.6)	15.9 (1.2)	13.5 (2.5)
*CC1/2*	0.999 (0.827)	0.998 (0.690)	0.998 (0.551)	0.999 (0.608)	0.999 (0.501)	0.996 (0.755)
Wilson B-factor (Å^2^)	44	24	115	92	103	21
Spherical completeness (%)	99.8 (99.2)	99.8 (99.5)	58.8 (13.6)	73.6 (25.3)	54.6 (7.8)	99.9 (99.9)
Ellipsoidal completeness (%)	–	–	93.5 (62.7)	95.2 (78.1)	93.4 (70.1)	–
Redundancy	8.6 (7.5)	4.0 (4.0)	5.1 (4.6)	6.8 (6.7)	5.3 (5.9)	4.6 (4.4)

**Refinement**						

Molecules/ASU	1	1	2	2	2	1
Reflections used for refinement	27,199 (2,681)	52,143 (5,114)	31,767	30,626	20,964	40,291 (3,982)
*R*_*work*_*/R*_*free*_ (%)	19.6/21.5	18.6/21.4	26.1/28.6	21.8/23.7	25.4/27.8	18.1/20.9
No. atoms	2,872	3,693	6,531	8,503	7,163	2,793
Protein	2,739	3,409	6,451	8,477	7,147	2,431
Water	128	276	70	*–*	16	349
*B*-factors (Å^2^)	51.5	29.8	89.1	91.9	80.8	29.3
Protein	51.5	29.1	89.2	92.0	80.8	27.9
Water	50.3	37.8	76.1	–	53.2	38.8
RMSDs						
Bond lengths (Å)	0.004	0.007	0.006	0.005	0.018	0.008
Bond angles (°)	0.92	1.21	1.05	1.00	1.33	1.16
Ramachandran statistics: favored/allowed/outliers (%)	96.6/3.4/0.0	97.8/2.2/0.0	96.7/3.3/0.0	96.4/3.5/0.1	95.8/4.0/0.2	99.3/0.7/0.0
Rotamer outliers (%)	0.0	0.0	12.9	3.3	8.1	0.0
Clashscore	3.8	3.3	13.4	9.3	19.7	1.7

Statistics for the highest-resolution shell are shown in parentheses. Merging statistics for anisotropy-corrected datasets were calculated from ellipsoidally truncated data. Asterisks indicate reciprocal cell directions.

Crystal structures of USP28, in which the insertion was included in the construct, yielded a dimeric entity in the asymmetric unit. An apo structure at 2.8 Å revealed a dimer of two USP28 catalytic domains (Figure 1F) that are spatially separated by 56 Å (distance between catalytic Cys171 residues) through helices from the domain insertion. Consistent with spatial separation, both catalytic domains are active, as confirmed by a second structure at 3.0 Å, in which each domain is modified by Ub-PA (Figure 1G). Despite the addition of Ub-PA, both structures crystallized in near-identical crystallographic settings, perhaps explaining similar relative orientations of individual catalytic domains and dimerization domains (Figure S1M). A kink in the extended helical stalk in one molecule of the dimer generates a slight asymmetry (Figure S1N). Catalytic domains are very similar between all four structures (RMSD < 1.2 Å).

**Figure 2:**
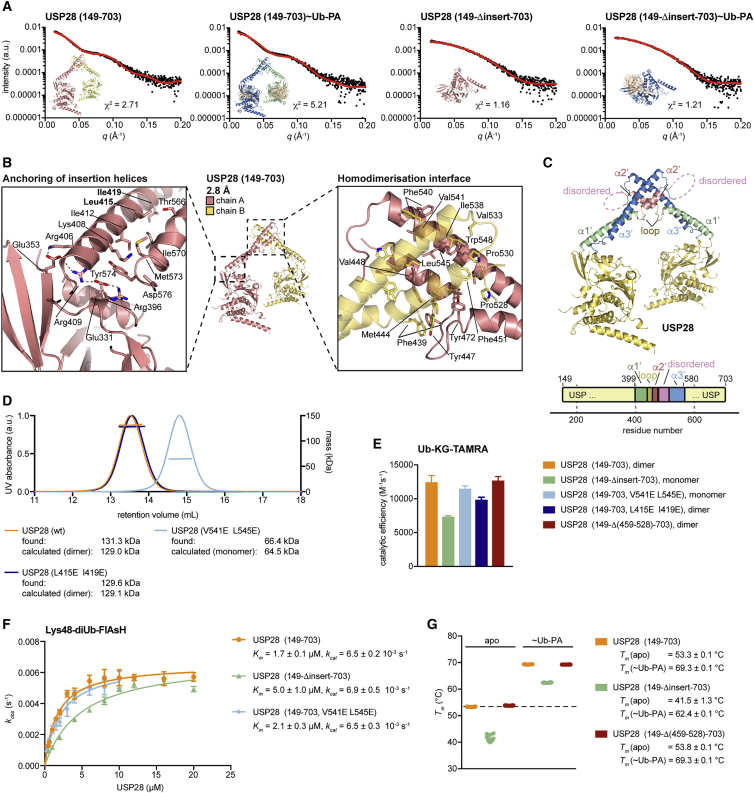
Structural Analysis of the USP28 Homodimerization Interface (A) Small-angle X-ray scattering (SAXS) data (black) collected from indicated protein samples. Expected scattering curves (red) were calculated from shown crystal structures. (B) Cartoon representation of USP28 (149–703). Boxed regions are magnified and highlight the connection of the insertion helices with the catalytic domain (left) and the dimerization interface (right). (C) Cartoon representation (top) and schematic representation (bottom) of the USP28 (149–703) structure with the insertion annotated. (D) SEC-MALS analysis of indicated proteins. Identified masses are matched to either monomeric or dimeric expected masses. (E) Catalytic activities of USP28 constructs determined from Ub-KG-TAMRA cleavage assays by fluorescence anisotropy measurements. Data are shown as mean ± SD from 2–5 independent experiments. See Figure S2F for anisotropy time courses. (F) Catalytic activities of USP28 constructs determined from Lys48-diUb-FlAsH cleavage assays. Data are shown as mean ± SE from 3 independent experiments performed in technical triplicates. Kinetic parameters obtained from fitted curves are listed. (G) Protein melting temperatures from thermal shift assays of indicated USP28 protein samples, either in the apo or Ub-PA-bound forms. Individual data points are plotted (*n* = 10), and melting temperatures are listed as mean ± SD.

It was unclear to what degree the distinctive dimeric structure was generated by crystal packing. Small-angle X-ray scattering (SAXS) is a suitable technique to experimentally determine the shape of molecules in solution and compare it to calculated data based on a structural model (Rambo and Tainer, 2013). Indeed, although insertion-lacking catalytic domains scattered as expected from a single globular domain, a distinct SAXS profile was observed from dimeric proteins that included the insertion (Figure 2A). Calculated scattering curves from the crystal structure models were an excellent fit for all SAXS measurements in solution (Figure 2A). SAXS data can also be expressed as pairwise distance distribution functions, which for monomeric USP28 samples revealed a single peak (*r* ∼35 Å), corresponding to internal distances within an isolated catalytic domain. Characteristically, dimeric USP28 proteins showed a second peak (*r* ∼80 Å) that closely reflected the average distance between the centers of mass of two catalytic domains in the crystal structures (Figure S2A; Table S1). Overall, SAXS data collected in solution were in excellent agreement with the dimeric USP28 crystal structure.

This suggested that the dimerized insertion domain is relatively rigidly anchored to the catalytic core. Molecularly, outgoing and incoming helices that form the stalk of the dimerization domain appeared indeed anchored to the catalytic domain by polar contacts (Figure 2B). The insertion of USP28 protrudes as an anti-parallel α-helical stalk from the base of the fingers subdomain. A 40-aa helix (α1′) at the start breaks to form a second, shorter (15-aa) helix (α2′), and both pack against an anti-parallel 60-aa helix (α3′) with hydrophobic interactions (Figures 2C and S2B–S2D). The α2′ and α3′ helices form a pseudo-symmetric, hydrophobic interface with the second molecule in the dimer (Figure 2B), with further contacts via the short loop between α1′ and α2′. Together, this generates a ∼100° angle between the helical stalks (Figure 2C). Intra- and intermolecular interface residues within the insertions are invariant in evolution (Figures S2B and S2C). A linker between the dimerization-helices α2′ and α3′ comprises 72 residues (aa 456–527) that are not conserved and disordered in the structures. Deletion of this region does not impact on dimerization behavior and has no apparent effect on activity *in vitro* (Figures 2E, S2E, and S2F).

Double-point mutations to disrupt dimerization (V541E L545E) or to destabilize the stalk of the insertion (L415E I419E) were introduced and showed expected monomeric or dimeric behavior on gel filtration (Figure 2D). Importantly, although removal of the insertion affected activity toward an Ub-KG-TAMRA substrate (Figures 2E and S1G), monomerized USP28 (V541E L545E) showed identical activity compared to dimeric USP28, and a variant with the insertion-destabilizing mutation (L415E I419E) was only mildly impaired in activity (Figure 2E).

We investigated the activity differences further by a full kinetic characterization using a Lys48-linked diUb-FlAsH substrate (Pruneda et al., 2016; Figure 2F). Wild-type and monomeric (V541E L545E) USP28 displayed virtually identical kinetic parameters, whereas deletion of the insertion led to a 3-fold reduction in *K*_*m*_ but no change in *k*_*cat*_. Thermal shift analysis revealed that deletion of the insertion led to a drastic decrease in protein stability in both the apo and Ub-PA-bound forms, whereas deletion of the disordered region in the center of the insertion did not affect protein stability (Figure 2G).

These data showed that the USP28 insertion per se has no strong impact on activity but indirectly contributes to stabilization of the catalytic domain and its ubiquitin-binding site. Notably, the insertion protrudes out of the catalytic domain just behind the distal ubiquitin binding site (Figure S1L). A role of the insertion in stabilizing the ubiquitin-binding site is further supported by the observation that parts of the fingers subdomain in the insert-deleted apo structure were disordered but ordered in Ub-PA-bound structures (Figures 1D and 1E). We conclude that USP28 is a dimer with separated, independently active catalytic domains (Figure S2G).

### Distinct Biophysical Behavior of USP25

**Figure 3:**
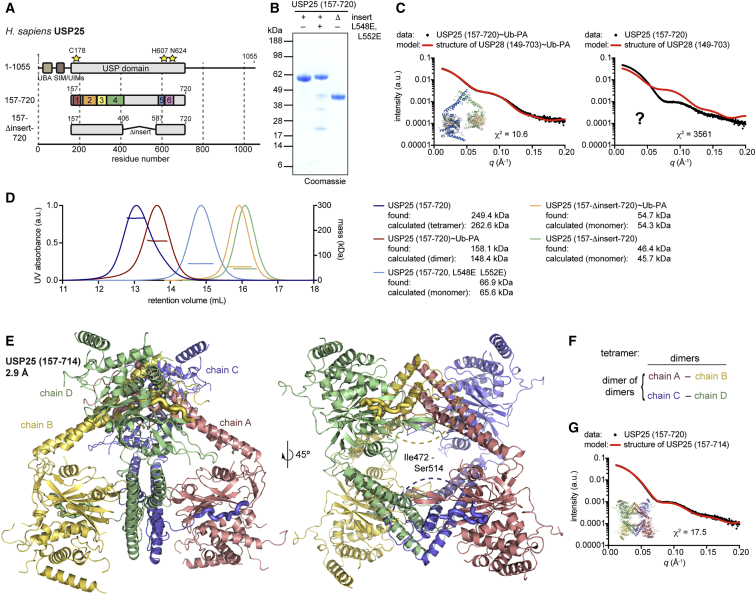
The Catalytic Domain of USP25 Is Tetrameric (A) Schematic representation of human USP25 constructs as in Figure 1A. A construct ending at residue 714 was used for crystallization. (B) Coomassie-stained SDS-PAGE gel of indicated recombinant proteins. (C) SAXS data (black) collected from indicated USP25 protein samples and overlaid with expected scattering curves (red) calculated from USP28 crystal structures, indicating the distinct scattering of apo USP25. (D) SEC-MALS analysis of indicated USP25 proteins. Identified masses are matched to either monomeric (light colors) or multimeric (dark colors) expected masses. (E) Crystal structure of the catalytic domain of USP25 in two orientations. Chains A and B constitute the asymmetric unit, and chains C and D are shown from symmetry-related molecules. Residues 515–528, which contact the catalytic domain of adjacent chains, are shown as thick tubes. Dotted lines indicate disordered residues. (F) Schematic of the USP25 tetramer as dimer of dimers. (G) SAXS data (black, repeated from C for clarity) overlaid with the expected scattering curve (red) calculated from the tetrameric USP25 crystal structure.

Next, we focused our attention on USP25, whose sequence has an overall identity and similarity with USP28 of 48% and 76%, respectively, and which includes a similar insertion. Corresponding catalytic domain constructs of USP25 with and without insertion were purified (Figures 3A and 3B) and compared by SAXS and SEC-MALS in apo and Ub-PA-bound states.

SAXS profiles of insertion-deleted constructs and of the insertion-containing covalent USP25∼Ub-PA complex were perfectly matched by calculated profiles from respective USP28 models (Figures 3C and S3A). Strikingly, however, the apo version of insertion-containing USP25 catalytic domain showed a distinctive SAXS profile that did not fit the profile derived from the USP28 apo structure (Figure 3C).

This was also reflected by SEC-MALS analysis. Monomeric behavior of insertion-lacking USP25 variants and dimeric behavior of the USP25∼Ub-PA complex contrasted a tetrameric behavior observed for the USP25 catalytic domain when the insertion was present (Figure 3D). SAXS distance distribution analysis revealed a broad, single-peak distribution, indicative of a large, globular shape (Figure S3B).

### Structure of Tetrameric USP25 Catalytic Domain

Attempts to crystallize this intriguing USP25 tetramer eventually resulted in a 2.9-Å crystal structure (Figures 3E, S3C, and S3D; Table 1). Interestingly, USP25 formed a dimer of dimers (Figure 3F), wherein the distinctive, V-shaped dimerization interface of one dimer inserted between catalytic domains of a second dimer, forming a symmetric tetramer (Figure 3E). Calculation of SAXS profiles from this structure generated a perfect fit for the previously unassigned experimental data (Figure 3G). Also, as expected from the ubiquitin-bound USP25 dimer observed in SAXS, the individual dimers of apo USP25 resembled USP28, with structurally similarly dimerized insertions and catalytic domains (Figures S3E–S3H). Superposition based on the dimerization domain reveals a slight rotation of catalytic domains with respect to the insertion; superposition based on the catalytic domain explains how this is generated by a different angle of how the insertions protrude from the catalytic core (Figure S3H). As in USP28, all residues that interact in *cis* and in *trans* are highly conserved throughout species (Figures S3E–S3G), and conserved residues coincide with residues that were observed to be structured (Figure S3F). Moreover, mutation of the equivalent set of hydrophobic residues in the dimerization interface (L548E L552E) led to the formation of monomeric USP25 (Figure 3D), further substantiating the notion that a dimeric arrangement is required for tetramerization.

### An Auto-inhibition Motif Links USP25 Oligomerization to Activity

Tetramerization orients catalytic domains such that ubiquitin-binding sites face outward and are accessible to solvent. Blocking loops 1 and 2, regulatory elements that line the C terminus of a bound ubiquitin (Clerici et al., 2014), are pried open by direct interactions with the insertion stalk provided by the second dimer.

**Figure 4:**
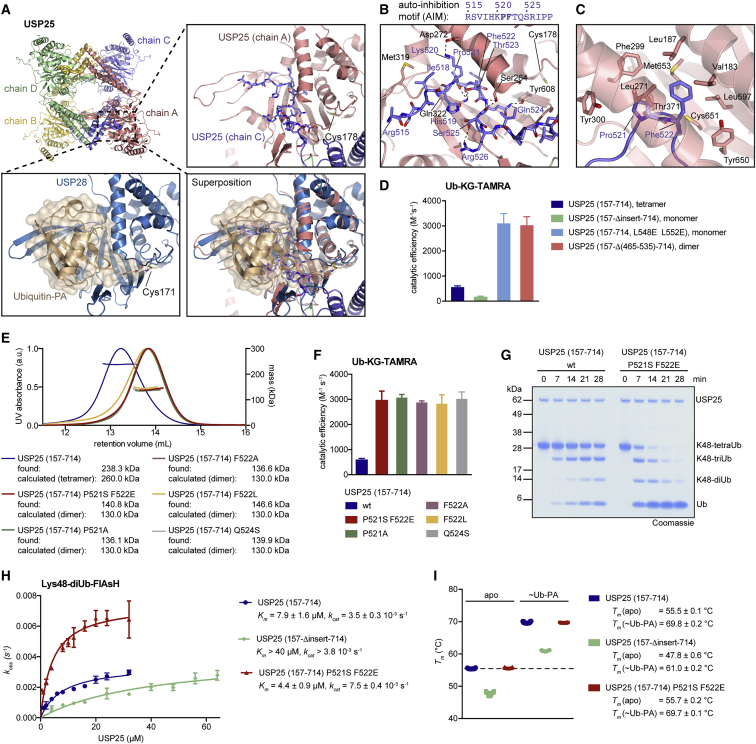
Tetramerization of USP25 Mediates Autoinhibition In *trans* through a Conserved AIM within the Domain Insertion (A) Top left: cartoon representation of the tetrameric catalytic domain of USP25. The area shown as close up is indicated. Top right: close-up view of chain A with the AIM of chain C shown as sticks is shown. Bottom left: USP28 (149-Δinsert-703) bound to Ub-PA under a semitransparent surface is shown. Bottom right: Superposition of the close ups shows the mutually exclusive binding of the AIM and ubiquitin. The catalytic cysteines Cys171 (USP25) and Cys178 (USP25) are shown for orientation. (B) Detailed view of the interaction between AIM and ubiquitin-binding site of USP25. Dashed lines indicate polar interactions. (C) A hydrophobic pocket in the USP25 catalytic domain accommodates Pro521 and Phe522 of the AIM. (D) Catalytic activities of USP25 constructs determined from Ub-KG-TAMRA cleavage assays by fluorescence anisotropy measurements. Data are shown as mean ± SE from 5 independent experiments. See Figure S4G for anisotropy time courses. (E) SEC-MALS analysis of indicated USP25 proteins. Identified masses are matched to either tetrameric or dimeric expected masses. (F) Catalytic activities of USP25 constructs analyzed in (E), determined from Ub-KG-TAMRA cleavage assays by fluorescence anisotropy measurements. Data are shown as mean ± SE from 3 independent experiments. See Figure S4H for anisotropy time courses. (G) Time course analysis of Lys48-linked tetraUb cleavage. The assay was performed three times with consistent results. (H) Catalytic activities of USP25 constructs determined from Lys48-diUb-FlAsH cleavage assays. Data are shown as mean ± SE from 3 independent experiments performed in technical triplicates. Kinetic parameters obtained from fitted curves are listed. (I) Protein melting temperatures from thermal shift assays of indicated samples. Individual data points are plotted (*n* = 10), and melting temperatures are listed as mean ± SD.

Importantly, we found that the tetramer was autoinhibited. A 14-residue section of the linker that is disordered in USP28 becomes ordered in USP25 and binds the ubiquitin binding site in a neighboring catalytic domain in the tetramer; this way all four catalytic domains are unable to bind ubiquitin (Figures 3E, 4A, and S4A–S4C). The ordered 14-mer autoinhibitory motif (AIM) is highly conserved in USP25 and centers on a Pro-Phe motif that inserts deeply into a grove below the α5 helix of the catalytic domain (Figures 4B, 4C, S4D, and S4E).

We confirmed the autoinhibitory nature of the AIM biochemically. Removal of the AIM in a construct that lacked all residues disordered in USP28 (construct 157-Δ(465-535)-714) led to dimeric USP25 (Figure S4F), which was 6-fold more active than tetrameric wild-type protein (Figures 4D and S4G). The same activity was obtained for a USP25 construct that included the AIM but was monomerized by mutations in the dimerization domain (L548E L552E). Deletion of the entire insertion (i.e., including the dimerization region) decreased activity, similar to what had been seen for USP28 (Figures 4D and S1G). Point mutations within the Pro-Phe motif or of the adjacent Gln524 disrupted the tetramer (Figure 4E) and activated USP25 accordingly for Ub-KG-TAMRA (Figures 4F and S4H) as well as polyubiquitin (Figure 4G) cleavage. Quantitative kinetic analysis of Lys48-diubiquitin-FlAsH cleavage revealed that the activation mediated by the P521S F522E mutation was due to the combination of a 2-fold higher *k*_*cat*_ and a 2-fold lower *K*_*m*_ (Figure 4H). As for USP28, deletion of the insertion of USP25 led to a more than 5-fold reduction in *K*_*m*_ (Figure 4H) and a large protein destabilization (Figure 4I). Tetrameric and dimeric forms of USP25 showed identical melting temperatures. These data confirm that the insertions of both USP25 and USP28 are important to stabilizing the catalytic domains and their ubiquitin-binding sites.

### Mechanism of the AIM

The AIM spans across the ubiquitin-binding site at the connection between the Palm and Fingers subdomains. The entire stretch would interfere with ubiquitin binding, as the AIM physically occupies much of the ubiquitin binding surface, forming numerous polar contacts (Figure 4B). Importantly, strong anchoring is provided by a section of the AIM, aa 519–524, that inserts itself into the core of the catalytic domain, occupying an unexpected pocket below the α5 helix. In particular, Pro521 and Phe522 rest deeply within the USP core (Figures 4C and S4A), and this interaction acts as a wedge that pushes the α5 helix away from the catalytic domain, e.g., when compared to apo and Ub-PA-bound states of USP28 (Figures S4D and S4E). A clear difference between inhibited and active structures is the conformation of an α5 hydrophobic core residue, Phe259 (USP25)/Phe266 (USP28), the sidechain of which rotates and moves >7 Å within the Palm subdomain; in active USP28, it anchors α5 in an active position and links the α5 helix to the activity state of the catalytic cysteine (Figure S4E).

The importance of correct α5 helix positioning as a regulatory mechanism has previously been noted also for USP7. A C-terminal activating peptide of USP7 binds on the opposite site of α5 compared to the AIM and seemingly pushes it into an active position (Figure S4D; Faesen et al., 2011, Rougé et al., 2016), and USP7 point mutations to improve α5 anchoring activate the enzyme (Özen et al., 2018). Together, this emphasizes the important role of α5 positioning in activating and inhibiting USP enzymes.

Cleavage of polyubiquitin requires the binding of a distal and of a proximal ubiquitin moiety across the active site. Superposition of tetrameric USP25 and of the structure of inactive USP30 in complex with Lys6-linked diubiquitin revealed that a catalytic domain of USP25 within the tetramer is unable to bind polyubiquitin because the proximal ubiquitin would clash with the helical stalks of another dimer (Figure S4I). Consequently, the ability of tetrameric USP25 to cleave polyubiquitin indicates that autoinhibited tetrameric and dimeric forms are in an equilibrium. Because disruption of the tetramer activates the enzyme for cleavage of longer chains, polyubiquitin alone seems insufficient to fully activate USP25 (Figure 4G). This transient dissociation of the tetramer would also explain that Ub-PA was able to generate a dimeric USP25 species (Figure 3D).

We conclude that USP25 forms an autoinhibited dimer of dimers, in which the insertion not only performs the key underlying dimerization role but also acts, via its AIM, as a regulatory element. We are not aware that a similar mechanism of activity regulation for another DUB has been described (Mevissen and Komander, 2017).

### A Dimer-Tetramer Equilibrium in Full-Length USP25

**Figure 5:**
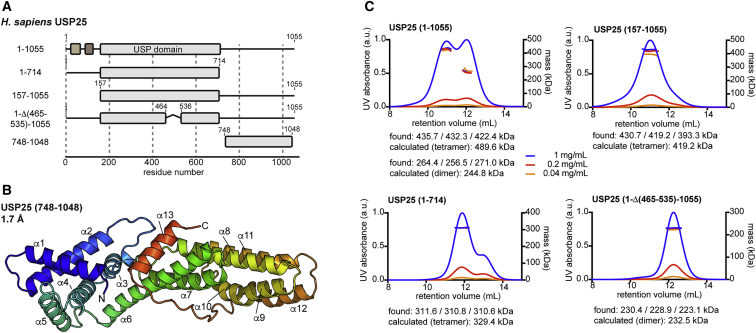
A Dimer-Tetramer Equilibrium in Full-Length USP25 (A) Schematic representation of human USP25 constructs as in Figure 3A. The deleted sequence between residues 464 and 536 corresponds to the sequence that was found to be disordered in USP28 and includes the AIM in USP25. (B) Crystal structure of the C-terminal domain of USP25 in spectral colors from blue (N terminus) to red (C terminus). α helices are numbered consecutively. (C) SEC-MALS analysis of a five-fold dilution series (blue: 1 mg/mL; red: 0.2 mg/mL; yellow: 0.04 mg/mL sample concentration) of indicated USP25 proteins. Identified masses are matched to either tetrameric or dimeric expected masses.

We next set out to understand whether regions outside of the catalytic domain can regulate the oligomeric state of USP25. The region N-terminal to the catalytic domain comprises several regulatory elements embedded into a flexible sequence context (Meulmeester et al., 2008, Zhen et al., 2014, Yang et al., 2017). A C-terminal region comprising ∼340 aa of unknown structure and function, yet with high conservation, is shared by USP25 and USP28 (Figures 5A, S5A, and S5B). We embarked on structural studies for this domain, excluding a C-terminal peptide in USP25 (aa 1,049–1,055) that facilitates an interaction with tankyrases to mediate their stabilization (Xu et al., 2017). A 1.7-Å crystal structure of USP25 (748–1,048) was solved by *ab initio* molecular replacement (Bibby et al., 2012) and revealed an α-helical domain (Figures 5B, S5C, and S5D; Table 1) without any similarity to previously reported structures in the protein data bank according to DALI (Holm and Laakso, 2016). The C-terminal region has been implicated in substrate binding for both USP25 and USP28 (Xu et al., 2017, Bosch-Comas et al., 2006, Serra et al., 2014) and harbors the insertion site for isoform-specific sequences.

High sequence similarity suggested a similar fold in USP28 (Figure S5E), which could not be crystallized from several constructs. Despite the relatively large size, NMR spectra for the 39-kDa ^15^N-labeled USP28 C-terminal domain (aa 736–1,077) were of high quality with discrete resonances, and relaxation behavior was consistent with a monomeric domain. NMR is an excellent method to pick up low-affinity interactions, yet the addition neither of unlabeled USP28 catalytic domain (aa 149–740) nor of unlabeled USP28 N terminus (aa 1–159) showed perturbation in the spectra, suggesting that the C-terminal domain forms an independent entity (Figure S5F). This was consistent with data that N- or C-terminal deletions of USP28 did not change its oligomerization or activity (Figures 1C and S1G).

In USP25, the situation was again different. C-terminally extended USP25 (aa 157–1,055) was exclusively tetrameric (Figures 5A and 5C), indicating that the C-terminal domain does not affect oligomeric behavior per se, as also found for USP28. In contrast, inclusion of the N terminus (aa 1–714 or full-length) unveiled a tetrameric species but also a dimeric species not present in constructs lacking the N terminus (Figures 5A and 5C). Although these data show that also full-length USP25 can adopt a tetrameric species from the dimerization of dimers, it hinted that the N terminus may regulate the oligomerization of USP25. A dimer-tetramer equilibrium as observed for full-length USP25 is consistent with the basal cleavage activity of tetrameric USP25 for polyubiquitin chains (Figures 4G and S4I).

### Validation of Oligomeric States in Cells

**Figure 6:**
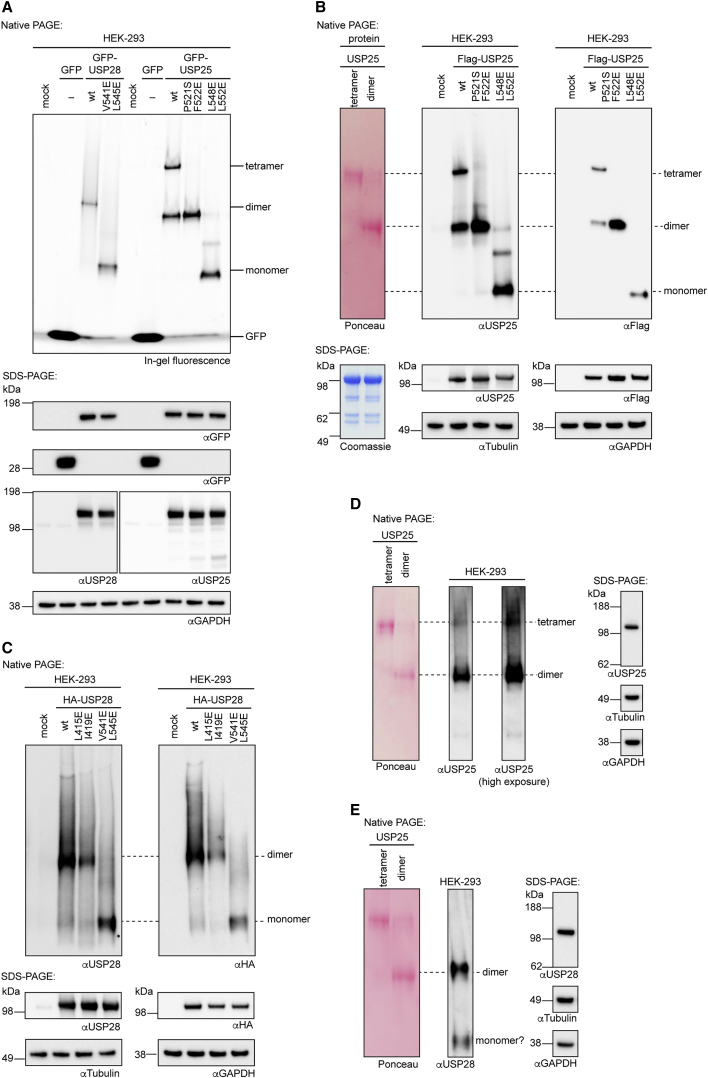
Analysis of Oligomerization States Adopted by USP25 and USP28 in Cells (A) Lysates from HEK293 cells transfected with vectors for the expression of GFP-tagged proteins or controls as indicated were analyzed by in-gel fluorescence after separation by native PAGE (top) or immunoblotting after separation by SDS-PAGE (bottom). (B) Lysates from HEK293 cells transfected with vectors for the expression of FLAG-tagged USP25 proteins or empty vector control as indicated were analyzed by immunoblotting after separation by native PAGE (top) or SDS-PAGE (bottom). As a size standard, tetrameric and dimeric fractions of recombinant, full-length human USP25 were used (see Figure S6A), visualized by Ponceau staining after native PAGE (top, same membrane as the blots to the right; see Figure S8 for uncropped blots and an overlay of the membranes) or Coomassie staining (bottom, separate gel). (C) Analysis as in (B) with indicated HA-tagged USP28 proteins. (D) Lysate of untreated HEK293 cells was separated by native PAGE (left) or SDS-PAGE (right) and analyzed by immunoblotting for endogenous USP25 as indicated. Size standard as in (B) is shown. (E) Analysis as in (D) with immunoblotting for endogenous USP28. For all assays, at least three independent experiments were performed and consistent results were obtained. See Figure S8 for uncropped blots.

We next sought to establish whether the distinct oligomeric states of USP28 and USP25 also form in cells. We transfected HEK293 cells with vectors encoding full-length, GFP-tagged versions of both proteins in wild-type and mutant forms and assessed their oligomerization in cell lysates by native PAGE and in-gel GFP fluorescence. We observed three oligomerization states that separated according to our predictions from the *in vitro* studies, and we assigned these as tetramer, dimer, and monomer (Figure 6A). Prompt processing of the samples after cell lysis by native PAGE was essential to observe the species assigned as tetramer (incubation of the lysate on ice for 5 h led to a decrease in its abundance, presumably due to its dissociation).

Because the migration behavior of protein complexes in native PAGE is dependent on their size as well as on their shape, we used recombinant full-length USP25 as a custom marker. Size-exclusion chromatography immediately before native PAGE analysis allowed the separation of almost exclusively tetrameric and dimeric samples (Figures 6B and S6A). When analyzed on a native PAGE gel alongside lysates from cells that were transfected with FLAG-tagged USP25 versions, immunoblotting allowed us to validate the identity of the species directly (Figure 6B). Ectopically expressed wild-type USP25 showed both tetrameric and dimeric populations with migration behavior identical to the recombinant protein samples, whereas USP25 mutated in the AIM (P521S F522E) was exclusively dimeric. Disruption of the dimerization interface (L548E L552E) led to monomeric protein in line with the *in vitro* findings. An equivalent experiment with hemagglutinin (HA)-tagged USP28 revealed that mutation of the dimerization interface (V541E L545E), but not mutations destabilizing the stalk (L415E I419E), changed the oligomerization in cells according to *in vitro* predictions (Figures 2D and 6C).

By immunoblotting for endogenous USP25 in HEK293 lysate following native PAGE, we observed an intense band corresponding to a dimeric oligomerization and a weak band that migrated exactly as the recombinant tetrameric species (Figure 6D). Albeit weaker, this band was consistently observed in three independent experiments, supporting that endogenous USP25 exists as a tetramer in cells, albeit at low levels under steady-state conditions. No tetrameric arrangement was observed in the analysis of endogenous USP28, which was mainly dimeric, with a second, much weaker band migrating at a size consistent with a monomer (Figures 6C and 6E).

Collectively, these results demonstrate that the distinctly different oligomerization states of USP25 and USP28 also exist in cell lysate, both in ectopically expressed and endogenous proteins. This establishes the relevance of the observed structural arrangements and corroborates the role of the AIM of USP25 in mediating the formation of a tetrameric arrangement in cells. Although we cannot exclude that other proteins partake in cellular USP25 or USP28 complexes, the similar sizes of purified and cellular endogenous proteins probably suggest that USP25 and USP28 are not exclusively stably associated with large-protein machineries.

### Functional Relevance of Oligomeric States

To investigate the functional consequences of disrupting the respective higher oligomeric states of USP25 and USP28, we assessed their ability to stabilize previously identified substrates in cells (Wu et al., 2013, Xu et al., 2017). When the chromatin modulator and histone demethylase LSD1 was co-expressed with USP28, both dimeric wild-type and monomeric mutant USP28 were able to stabilize LSD1 to the same degree in a concentration-dependent manner (Figure S6B). Thus, the dimeric arrangement of USP28 may have a functional relevance in other cellular contexts, but it seems dispensable for the stabilization of LSD1, in line with *in vitro* activity data (Figures 2E and 2F).

Further, we observed that strictly dimeric, mutant USP25 (P521S F522E) was more effective in stabilizing ectopically expressed tankyrase-2 than wild-type USP25 (Figure S6C). The small but consistent increase in substrate levels (Figure S6D) is in line with about one-third of the wild-type proteins forming an autoinhibited tetramer in cells under the conditions tested (see Figure 6B). These results suggest a functional role of the AIM in mediating autoinhibition by the coupling of autoinhibition and oligomerization in a cellular context.

### Molecular Distinction of USP25 and USP28

**Figure 7:**
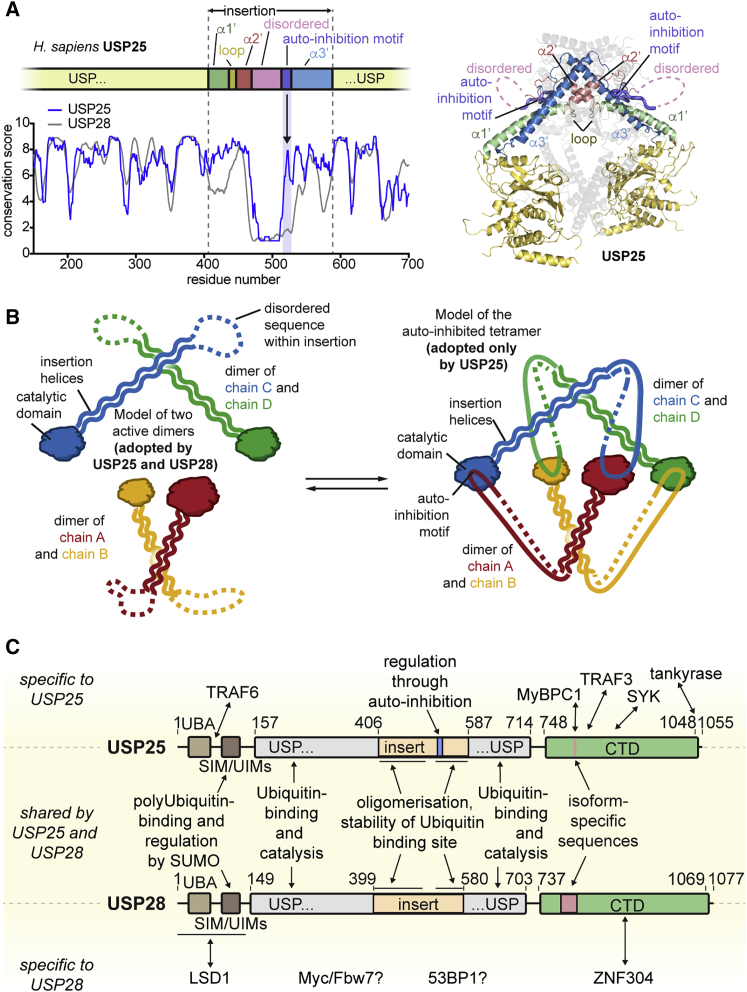
Molecular Comparison of USP28 and USP25 (A) Schematic representation of the sequence of the USP25 catalytic domain with parts of the insertion highlighted in different colors. Sequence conservation of USP25 and USP28 catalytic domains plotted as a 5-residue rolling window average of scores obtained from the ConSurf webserver (left). Cartoon representation of chains A and B of the USP25 crystal structure with the same color code (right). The other chains completing the tetramer are shown as transparent cartoons in gray. The spike in the USP25 sequence conservation at residue 522 corresponding to the AIM is highlighted with a black arrow. (B) Cartoon model showing how tetramerization facilitates autoinhibition in *trans* in USP25 (left and right), but not USP28 (left). (C) Schematic representation of domain topologies of human USP25 and USP28. Boundaries of indicated domains and motifs are shown with residue numbers. Interactions and features either specific to USP25 (top), shared by USP25 and USP28 (middle), or specific to USP28 (bottom) are linked to the relevant sequence regions with black arrows.

The AIM in USP25 is strictly conserved (Figure 7A), whereas the equivalent sequence in USP28, which was disordered in the structures (Figures 1F and 1G), lacks conservation (Figure 7A). This suggests that autoinhibition by tetramerization has been a feature specific to USP25, but not USP28, throughout evolution (Figure 7B). To experimentally investigate this hypothesis, we assessed the oligomeric states of selected orthologs. USP25 catalytic domains from chicken (*Gallus gallus*) and mouse (*Mus musculus*) were tetrameric but became dimeric upon Ub-PA binding (Figure S7A). Like full-length human USP25 (Figure S3J), also full-length mouse USP25 showed a dimer-tetramer equilibrium (Figure S7B). In contrast, human USP28 did not display any sign of tetrameric behavior *in vitro* and in cells (Figures 1, 2, and 6). Importantly, USP28 catalytic domains from chicken and zebrafish (*Danio rerio*), and full-length zebrafish USP28, were exclusively dimeric (Figures S7A and S7B).

To establish the molecular requirements for tetramer formation of USP25, we created chimeric catalytic domains of USP25 and USP28 in which either the entire inserts (chimeras 1 and 3) or the AIM of USP25 and its equivalent sequence in USP28 (chimeras 2 and 4) were swapped (Figure S7C). SEC-MALS analysis revealed that all chimeras were clean dimers (Figure S7D), which shows that the USP25 autoinhibition motif is necessary (chimera 4 is dimeric), but not sufficient (chimeras 1 and 2 are dimeric), for tetramer formation. Instead, the molecular combination of the USP25 catalytic domain and the USP25 insertion, including its AIM, was strictly required for tetramer formation. To our surprise, we noticed sequences in several USP28 homologs that were near identical to the AIM of human USP25 (Figures S2C and S3F). The most prominent case was zebrafish USP28, which shares the central IHKPFTQ motif with human USP25 but has mutations in the flanking parts and is still dimeric (see chimera 5 in Figure S7D and Figure S7B). This shows that subtle changes within the AIM or around it are sufficient to prevent tetramerization.

These data help define the molecular distinction between USP25 and USP28. They show that, although the AIM is necessary, but not sufficient, for tetramerization (and autoinhibition) in USP25, the equivalent motif in USP28 is unable to induce tetramerization. Although potentially restrained by crystal packing and more flexible in solution, it is worth noting that USP25 and USP28 dimers showed large differences in the relative positions of catalytic domains with respect to insertion and AIM (Figure S3H), and this may also prevent regulatory USP28 tetramerization.

## Discussion

We here provide a molecular comparison of two USP enzymes, USP25 and USP28, which are highly similar at the sequence level, yet display striking differences in their oligomerization capabilities and concomitant regulation of enzymatic activities. Our data show that the catalytic domains of USP25 and USP28 share insertion sequences that (1) support distal ubiquitin binding, (2) are important for protein stability, and (3) mediate oligomerization in surprisingly distinct ways not anticipated from sequence similarity alone (Figures 7B and 7C). The presence of a conserved AIM in USP25 family members endows USP25 with a paralog-specific link of oligomerization and activity. Such coupling of oligomerization and activity is reminiscent of, e.g., UDP-α-d-glucose-6-dehydrogenase, which forms an inactive hexamer that can be broken into active dimers through allosteric regulation (Keul et al., 2018). Evolutionarily, the described mechanism likely originated from the dimer, as its shape constitutes a requirement for the formation of the observed autoinhibited tetramer (Figure 3D). All of our mechanistic insights are in full agreement with a recent crystal structure of a longer USP25 fragment in an autoinhibited tetrameric state, which forms a dimer-tetramer equilibrium in solution (Liu et al., 2018).

In addition, we validated the oligomeric differences of USP25 and USP28 in cells and observed endogenous USP25 to be mainly dimeric with a small tetrameric population (Figure 6). This establishes the relevance of the observed oligomerizations and will fuel future work into how cells utilize these enzymatic states with different activities e.g., during signaling.

Because the tetramer of USP25 was observed only in small quantities at the endogenous level but readily formed upon overexpression, it is tempting to speculate that autoinhibition of USP25 is regulated by local concentrations. Rather than asking how autoinhibition is relieved, it may be sensible to ask how and under which conditions the tetramer is formed and the enzyme is inactivated. Molecular crowding of USP25 (by means of recruitment to, e.g., TRAF E3 ligase complexes; see Introduction) could lead to tetramer formation and quench the local deubiquitination activity, potentially enabling a negative feedback loop. Although ubiquitin, in principle, is able to overcome the autoinhibition of tetrameric USP25, the presence of polyubiquitin in the micromolar concentration range is insufficient for full activation (Figure 4D). However, we cannot exclude a regulatory role by polyubiquitin at higher local concentration. Future studies should investigate the interplay of USP25 oligomerization with ligase assemblies at their receptors.

The existence of USP28 as a constitutive dimer is also intriguing. USP28 has multiple substrates, including the important proteins FBW7, c-MYC, and 53BP1, and our structure-based refinement of the USP28 domain architecture will aid the identification of their binding mechanisms (Figure 7C). Many cullin-SCF E3 ligase complexes are dimeric, which is often facilitated by adaptor dimerization; this concept extends to FBW7 (Tang et al., 2007). Adaptation of a specific dimeric USP structure in which catalytic domains are independently active and spatially separated may invoke intriguing mechanisms of how ligase-DUB interplay is established. Despite dimerization of USP28 being dispensable for the stabilization of LSD1 (Figure S7B), future studies including putative ubiquitinated cullin-based substrates could lead to further mechanistic and biological insights into USP biology.

Collectively, we have identified molecular underpinnings of how two very similar USP enzymes can be functionally different, further highlighting that gene duplication does not necessarily facilitate functional redundancy. It is fascinating how subtle changes in sequence regulate oligomeric behavior with direct impact on enzymatic activity. All of the described features of USP25 and USP28 arise from sequences outside the core catalytic domain (Figure 7C). Our data illustrate a conceptually intriguing interplay of catalytic and non-catalytic elements in USP DUBs to achieve diverging functions of highly homologous enzymes and, for USP25, also reveal a mechanism of autoinhibition that could be pharmaceutically explored. In this respect, USP enzymes are rich in regulatory capabilities and will continue to provide a fertile ground for future discoveries, as well as provide new pharmaceutical targets.

## STAR★Methods

### Key Resources Table

**Table:** 

REAGENT or RESOURCE	SOURCE	IDENTIFIER
**Antibodies**		

Rabbit monoclonal anti-USP25	abcam	Cat. # ab187156
Rabbit polyclonal anti-USP28	proteintech	Cat. # 17707-1-AP
Mouse monoclonal anti-Flag M2	Sigma	Cat. # F3165
Mouse monoclonal anti-HA	BioLegend	Cat. # 16B12
Goat polyclonal anti-GFP	abcam	Cat. # ab6673
Mouse monoclonal anti-GFP JL-8	TaKaRa	Cat. # 632381
Mouse monoclonal anti-GAPDH	Ambion	Cat. # AM4300
Mouse monoclonal anti-Tubulin	Sigma	Cat. # T6199
Sheep anti-mouse IgG-HRP	Sigma	Cat. # GENXA931
Donkey anti-rabbit IgG-HRP	Sigma	Cat. # GENA934
Donkey anti-goat IgG-HRP	Santa Cruz Biotech	Cat. # sc-2020

**Bacterial and Virus Strains**		

*E. coli* TOP10	Thermo Fisher	Cat. # C404010
*E. coli* Rosetta2(DE3)pLacI	Merck	Cat. # 71404

**Chemicals, Peptides, and Recombinant Proteins**		

Ubiquitin-KG-TAMRA	P. Geurink, H. Ovaa (LUMC)	n/a
KG-TAMRA	P. Geurink, H. Ovaa (LUMC)	n/a
NT-495 dye	Nanotemper	Cat. # MO-L003
BSA	Pierce	Cat. # 23209
Polyethylenimine, Linear, MW 25000	Polysciences	Cat. # 23966

**Critical Commercial Assays**		

Phusion High-Fidelity DNA Polymerase	NEB	Cat. # M0530
Clarity Western ECL substrate	Bio-Rad	Cat. # 1705060
SuperSignal Femto Maximum Sensitivity Substrate	ThermoFisher	Cat. # 34094

**Deposited Data**		

Structure of USP28 (149-703)	This study	PDB: 6HEJ
Structure of USP28 (149-703)∼Ub-PA	This study	PDB: 6HEK
Structure of USP28 (149-Δinsert-703)	This study	PDB: 6HEH
Structure of USP28 (149-Δinsert-703)∼Ub-PA	This study	PDB: 6HEI
Structure of USP25 (157-714)	This study	PDB: 6HEL
Structure of USP25 (748-1048)	This study	PDB: 6HEM

**Experimental Models: Cell Lines**		

HEK293	ATCC	Cat. # CRL-1573

**Recombinant DNA**		

Codon-optimized human USP28 gene in pMK-RQ	This study	MG-22-21
Human USP28 (1-1077) in pOPINB	This study	MG-31-04
Human USP28 (1-Δinsert-1077) in pOPINB	This study	MG-31-05
Human USP28 (1-703) in pOPINB	This study	MG-31-02
Human USP28 (1-Δinsert-703) in pOPINB	This study	MG-31-03
Human USP28 (149-703) in pOPINB	This study	MG-26-50
Human USP28 (149-Δinsert-703) in pOPINB	This study	MG-26-54
Human USP28 (149-703) V541E L545E in pOPINB	This study	MG-31-12
Human USP28 (149-703) L415E I419E in pOPINB	This study	MG-31-15
Human USP28 (149-Δ(459-528)-703) in pOPINB	This study	MG-31-20
Human USP25 (157-720) in pOPINB	This study	MG-31-70
Human USP25 (157-Δinsert-720) in pOPINB	This study	MG-31-72
Human USP25 (157-720) L548E L552E in pOPINB	This study	MG-31-71
Human USP25 (157-714) in pOPINB	This study	MG-32-75
Human USP25 (157-Δinsert-714) in pOPINB	This study	MG-32-95
Human USP25 (157-714) L548E L552E in pOPINB	This study	MG-32-96
Human USP25 (157-Δ(465-535)-714) in pOPINB	This study	MG-32-77
Human USP25 (1-1055) in pOPINB	This study	MG-32-71
Human USP25 (1-714) in pOPINB	This study	MG-32-72
Human USP25 (157-1055) in pOPINB	This study	MG-32-89
Human USP25 (1-Δ(465-535)-1055) in pOPINB	This study	MG-32-93
*D. rerio* USP28 (138-684) in pOPINB	This study	MG-38-47
*G. gallus* USP28 (143-706) in pOPINB	This study	MG-38-55
*M. musculus* USP25 (157-715) in pOPINB	This study	MG-38-53
*G. gallus* USP25 (174-726) in pOPINB	This study	MG-38-57
*M. musculus* USP25 (1-1055) in pOPINB	This study	MG-38-52
*D. rerio* USP28 (1-738,763-1187) in pOPINB	This study	MG-38-46
Human USP25 (157-714) P521S F522E in pOPINB	This study	MG-38-84
Human USP25 (157-714) P521A in pOPINB	This study	MG-38-85
Human USP25 (157-714) F522A in pOPINB	This study	MG-38-86
Human USP25 (157-714) F522L in pOPINB	This study	MG-38-87
Human USP25 (157-714) Q524S in pOPINB	This study	MG-38-88
Chimera: Human USP25 (157-[Insert of Human USP28]-714) in pOPINB	This study	MG-38-79
Chimera: Human USP28 (149-[Insert of Human USP25]-703) in pOPINB	This study	MG-38-80
Chimera: Human USP25 (157-[AIM of Human USP28]-714) in pOPINB	This study	MG-38-81
Chimera: Human USP28 (149-[AIM of Human USP25]-703) in pOPINB	This study	MG-38-82
Chimera: Huma USP25 (157-[AIM of *D. rerio* USP28]-714) in pOPINB	This study	MG-38-83
Human USP25 (748-1048) in pOPINB	This study	MG-31-80
Human USP28 (736-1077) in pOPINB	This study	MG-31-10
GFP in pOPINE	This study	MG-52-30
GFP-USP28 (2-1077) in pOPINE	This study	MG-52-31
GFP-USP28 (2-1077) V541E L545E in pOPINE	This study	MG-52-69
GFP-USP25 (2-1055) in pOPINE	This study	MG-52-64
GFP-USP25 (2-1055) P521S F522E in pOPINE	This study	MG-52-65
GFP-USP25 (2-1055) L548E L552E in pOPINE	This study	MG-52-68
Flag-USP25 (2-1055) in pOPINE	This study	MG-52-49
Flag-USP25 (2-1055) P521S F522E in pOPINE	This study	MG-52-50
Flag-USP25 (2-1055) L548E L552E in pOPINE	This study	MG-32-08
HA-USP28 (2-1077) in pOPINE	This study	MG-23-34
HA-USP28 (2-1077) V541E L545E in pOPINE	This study	MG-32-13
HA-USP28 (2-1077) L415E I419E in pOPINE	This study	MG-32-23
HA-USP28 (2-1077) C171A in pOPINE	This study	MG-32-21
HA-Tankyrase-2 (2-1166) in pOPINE	This study	MG-52-35
Flag-LSD1 (2-852) in pOPINE	This study	MG-52-33

**Software and Algorithms**		

XDS Version November 11, 2017	W. Kabsch (MPI Heidelberg)	http://homes.mpimf-heidelberg.mpg.de/∼kabsch/xds/
Autoproc	Global Phasing Ltd	https://www.globalphasing.com/
CCP4 7.0.060	CCP4 team	http://www.ccp4.ac.uk/
Phenix 1.13-2998	Phenix team	https://www.phenix-online.org/
Staraniso webserver	Global Phasing Ltd	http://staraniso.globalphasing.org
AMPLE	Bibby et al., 2012	http://pcwww.liv.ac.uk/∼drigden/
ScÅtter 3.0	R. Rambo (DLS Didcot)	http://www.bioisis.net/
Fast SAXS Profile Computation with Debye Formula webserver	Schneidman-Duhovny et. al. 2016	https://modbase.compbio.ucsf.edu/foxs/
Pymol v1.8.2.2	Schrödinger LLC	https://pymol.org/2/
CONCORD webserver	Wei et al., 2011	http://helios.princeton.edu/CONCORD/
ConSurf webserver	Biosof LLC	http://consurf.tau.ac.il/2016/
DALI webserver	Holm and Laakso, 2016	http://ekhidna2.biocenter.helsinki.fi/dali/
Pro-origami webserver	A. Stivala (U Melbourne)	http://munk.cis.unimelb.edu.au/pro-origami/
NetWheels	A. Mól, W. Fontes, M. Castro (U Brasília)	http://www.lbqp.unb.br/NetWheels/
ESPript 3.0	P. Gouet and X. Robert (Lyon)	http://espript.ibcp.fr/ESPript/ESPript/
Astra 6.1	Wyatt Technology	https://www.wyatt.com/products/software/astra
Topspin 3.1	Bruker	https://www.bruker.com
Image Lab	Bio-Rad	http://www.bio-rad.com/en-uk/product/image-lab-software?ID=KRE6P5E8Z
Illustrator CS6	Adobe	https://www.adobe.com/uk/products/illustrator.html

### Contact for Reagent and Resource Sharing

Requests for resources and reagents should be directed to and will be fulfilled by the lead contact, David Komander (dk@wehi.edu.au).

### Experimental Model and Subject Details

HEK293 cells (ATCC CRL-1573) were maintained in DMEM + GlutaMAX supplemented with 10% (v/v) fetal bovine serum (FBS) and penicillin-streptomycin at 37°C in a humidified atmosphere containing 5% CO_2_. Cells were tested negative for mycoplasma contamination with a Lonza MycoAlert Assay.

### Method Details

#### Cloning and constructs

Human USP28 constructs were cloned from a gene obtained from GeneArt (Thermo Fisher) with codon-optimization for bacterial expression. Human USP25, LSD1 and TNKS2 were cloned from vectors kindly provided by Sylvie Urbe, Bradley Bernstein (Addgene plasmid #49042) and Marc de la Roche, respectively. Mouse, chicken and zebrafish orthologs were cloned from cDNA libraries.

Protein sequences correspond to Uniprot entries Q96RU2 (*H. sapiens* USP28), Q9UHP3 (*H. sapiens* USP25), P57080 (*M. musculus* USP25), Q5ZID5 (*G. gallus* USP28), F1NCR4 (*G. gallus* USP25), E7FD72 (*D. rerio* USP28), O60341 (*H. sapiens* LSD1) and Q9H2K2 (*H. sapiens* TNKS2).

Constructs were assembled using In-Fusion cloning (Clontech) into pOPINB (all constructs for bacterial expression) or pOPINE (constructs for transient transfection of mammalian cells with N-terminal tags as indicated) vectors and amplified in *E. coli* TOP10 cells (Thermo Fisher). Site-directed mutagenesis was carried out using the QuikChange method or with overlap extension PCR. Boundaries were chosen according to secondary structure predictions using the Concord webserver (Wei et al., 2011). A triple GS linker was used to replace deleted sequences in constructs where a deletion is indicated.

GFP-tagged constructs were cloned with a monomeric (A206K) eGFP sequence.

#### Protein expression and purification

Expression was carried out in *E. coli* Rosetta2(DE3)pLacI cells (Merck) that were chemically transformed with expression vector and subsequently grown in 2xTY media supplemented with kanamycin (50 mg/L) and chloramphenicol (34 mg/L). Cultures were grown at 37°C for ∼3-4 h until an OD_600_ of 1.0 and then cooled to 18°C for ∼1 h. Expression was induced by addition of 0.5 mM isopropyl-β-d-1-thiogalactopyranoside (IPTG) and cultures were kept shaking at 18°C over night. Following harvest by centrifugation, cell pellets were flash-frozen in liquid nitrogen and stored at –80°C. ^15^N-labeled protein was obtained from expression in M9(–) media supplemented with 1 g/L ^15^N-ammonium chloride and as described otherwise.

All purification steps were carried out on Äkta Explorer, Äkta Purifier and Äkta Pure systems (GE Healthcare) at 4°C. Pellets were resuspended in buffer A (50 mM sodium phosphate pH 8.0, 300 mM NaCl, 4 mM β-mercaptoethanol, 20 mM imidazole) supplemented with DNaseI and lysozyme. After lysis by sonication (4 min, 55% power, 10 s on / 10 s off, Fisherbrand FB705 sonicator), the lysate was cleared by centrifugation (22 000 rpm, 30 min, 4°C), filtered, and loaded on 5 mL HisTrap FF columns. Elution was carried out with a gradient over 6 column volumes (CV) into buffer B (as buffer A, with 500 mM imidazole). Protein containing fractions were pooled, supplemented with 3C protease and dialyzed into buffer C (25 mM Tris pH 8.5, 50 mM NaCl, 5 mM DTT) for 2-24 h at 4°C.

For anion-exchange chromatography, samples were loaded onto a 6 mL Resource Q column equilibrated in buffer C and eluted with a 20 CV gradient into buffer D (25 mM Tris pH 8.5, 500 mM NaCl, 5 mM DTT). Peak fractions were pooled, concentrated via centrifugation and loaded onto HiLoad 16/600 Superdex 200 pg or Superdex 75 pg columns for size-exclusion chromatography in buffer E (20 mM Tris pH 8.0, 100 mM NaCl, 5 mM DTT) or buffer F (as buffer E, with 5% (v/v) glycerol). The purity of peak fractions was assessed by SDS-PAGE. Fractions were concentrated at 4°C and 3,200 xg in spin concentrators (10 kDa MWCO, Viva spin) and flash-frozen in liquid nitrogen. All protein concentrations were determined by absorption at 280 nm unless noted otherwise.

#### Covalent ubiquitin-propargylamine complexes

Ub(1-75)-PA and His_6_-3C-Ub(1-75)-PA suicide probes were assembled as described previously (Gersch et al., 2017) and analyzed by intact protein mass spectrometry confirming > 95% purity (expected mass of the His_6_-3C-Ub(1-75)-PA probe: 10 657 Da, mass found: 10 657 Da).

Covalent attachment of His_6_-3C-Ub(1-75)-PA to USP25/USP28 proteins was carried out following the anion exchange step. A 1.3 eq molar excess of probe was spiked into the pooled elution fractions and allowed to react for 1–4 h at 4°C. Dialysis into buffer A was carried out and the sample was loaded onto a 5 mL HisTrap for affinity chromatography. Following elution with buffer B, the protein complex was treated with 3C protease for 1 h at 4°C and subjected to gel filtration chromatography into buffer E as described above.

#### Crystallization

Crystallization experiments were carried out in 96-well sitting-drop vapor diffusion plates in MRC format (Molecular Dimensions) at 18°C and set up using a mosquito HTS robot (TTP Labtech). Typical drop ratios of 200 nL + 200 nL and 500 nL + 500 nL (protein solution + reservoir solution) were used for coarse screening and fine screening, respectively, unless noted otherwise.

USP28 (149-703) was concentrated to 13.3 mg/mL in buffer F and crystallized in 0.8 M ammonium sulfate, 100 mM sodium citrate pH 5.5 as large cuboids (∼200x100x40 μm^3^). The crystal-containing drop was overlaid with an equal volume of cryo solution made of reservoir (three parts) and glycerol (one part) and incubated for 2 min. Crystals were then transferred into the pure cryo solution and harvested after another 2 min incubation.

USP28 (149-703)∼Ub-PA was concentrated to 14.1 mg/mL in buffer F and crystallized in 8% (w/v) PEG 3350, 100 mM sodium citrate pH 5.4 and 200 mM ammonium acetate as large cuboids (250x140x100 μm^3^). Cryoprotection was achieved by swiping the crystal quickly through a cryo solution containing (20% (v/v) PEG 400, 10% (w/v) PEG 3350, 100 mM sodium citrate pH 5.6, 200 mM ammonium acetate) followed by immediate vitrification in liquid nitrogen.

USP28 (149-Δinsert-703) was concentrated to 15 mg/mL in buffer E and crystallized in 12% (w/v) PEG 8000, 100 mM sodium chloride, 200 mM lithium sulfate, 100 mM MES pH 6.6 as large cubes. Crystals were cryoprotected with a solution containing the components of the reservoir and 25% (v/v) ethylene glycol.

USP28 (149-Δinsert-703)∼Ub was concentrated to 25 mg/mL in buffer E and crystallized in 22% (w/v) PEG 3350, 300 mM potassium sodium tartrate, yielding small cuboid crystals (40x50x90 μm^3^) growing to their maximal size within 7 days. Cryoprotection was achieved by transferring the crystals into a solution containing the components of the reservoir and 25% (v/v) ethylene glycol.

USP25 (157-714) was concentrated to 11.5 mg/mL in buffer E and crystallized in 12% (w/v) PEG 3350, 167 mM magnesium acetate as long rods (2000x100x100 μm^3^). Crystals were harvested into a cryo solution containing 12% (w/v) PEG 3350, 150 mM magnesium acetate, 20 mM Tris pH 8.0, 25% (v/v) glycerol, incubated for 5 min and vitrified in liquid nitrogen.

USP25 (748-1048) was concentrated to 11.6 mg/mL in buffer E and crystallized in 20% (w/v) PEG 4000, 600 mM sodium chloride, 100 mM MES pH 6.5 as needles (300x50x30 μm^3^). Crystals were cryoprotected by transferring them into a solution containing the components of the reservoir and 25% (v/v) glycerol.

#### Data collection, structure solution and refinement

Diffraction data were collected at 100 K at the Diamond Light Source (DLS), Harwell, UK on beamlines I02, I03 and I04-1, and at the European Synchrotron Radiation Facility (ESRF), Grenoble, France on beamlines ID29 and ID30B. Datasets leading to structures of USP28 (149-Δinsert-703), USP28 (149-Δinsert-703)∼Ub-PA and USP25 (748-1048) were processed using XDS (Kabsch, 2010), and scaled using Aimless (Evans and Murshudov, 2013) in the CCP4 suite of programs. The datasets leading to the structures of USP28 (149-703), USP28 (149-703)∼Ub-PA and USP25 (157-714) showed a high degree of anisotropy, and anisotropy correction was hence performed using the STARANISO web server (http://staraniso.globalphasing.org). For this purpose, the data were indexed and integrated in the respective space groups by XDS (USP28 (149-703)∼Ub-PA and USP25 (157-714)) or autoproc (USP28 (149-703)) and the unmerged datasets were submitted to the STARANISO server. *R*_*free*_ flags were then added to the merged and ellipsoidally scaled output data using the Phenix reflection file editor. Anisotropy correction greatly improved the quality and interpretability of the electron density maps and was critical for converging refinement runs.

The structure of USP28 (149-Δinsert-703)∼Ub-PA was solved by molecular replacement using MR Phaser (McCoy et al., 2007) and a chainsaw-derived search model of USP7 in complex with ubiquitin (PDB: 1NBF). Model building using Coot (Emsley et al., 2010) and refinement with Phenix.Refine (Adams et al., 2011) yielded the final structure that was used as a search model to solve the apo structure of USP28 (149-Δinsert-703).

The coordinates of the USP28 (149-Δinsert-703)∼Ub-PA structure were then used as a search model to phase the data leading to the structure of USP28 (149-703)∼Ub-PA. Two copies of the search model could be placed, and clear helical density extending away from the catalytic USP domain was visible. Several rounds of manual model building using Coot, refinement by Refmac (with external restraints against the high-resolution structure of USP28 (149-Δinsert-703)∼Ub-PA generated by ProSmart (Nicholls et al., 2012)) and Phenix.Refine were used to arrive at the final model. The sequence register of the domain insertion could be assigned unambiguously due to the clear connectivity to the catalytic domain. The apo structure of USP28 (149-703) was solved by molecular replacement using two copies of the high resolution catalytic domain structure of USP28 (149-Δinsert-703) as well as one copy of the dimeric insertion sequence of the USP28 (149-703)∼Ub-PA structure as search models.

The dataset leading to the structure of USP25 (157-714) was initially processed in spacegroup *I*422 and solved by molecular replacement using chainsaw models of the catalytic domain derived from the USP28 (149-Δinsert-703) structure and the insertion sequence of one copy of the USP28 (149-703)∼Ub-PA structure. However, refinement stalled at an *R*_*free*_ of ∼37% with little interpretable difference density. The dataset was hence reprocessed in space group *P*1 with anisotropy correction as described above. Space group validation runs were carried out with the Zanuda pipeline as implemented in the CCP4 suite of programs using models obtained from perturbation of the intermediate *I*422 model with Phenix.Dynamics. Consistently, automated refinement with Refmac resulted in a ∼2%–3% high *R*_*free*_ in *I*422 compared to all other space groups (*C*2, *I*222, *F*222, *I*4). The final model was obtained from anisotropy-corrected data processed in spacegroup *I*4 and refined with Phenix.Refine to an *R*_*free*_ of 27.8%.

Secondary structure prediction of the USP25 C-terminal sequence suggested a high α-helical content. The structure of USP25 (748-1048) was subsequently solved by *ab initio* molecular replacement with 15 residue polyalanine helices as implemented in AMPLE (Bibby et al., 2012) with an automated pipeline using MR Phaser for generating initial phases, SHELXE for phasing and density modification, Refmac for refinement and ArpWarp for model building, yielding a near-finished model with an *R*_*free*_ of 26%. Further rounds of refinement were carried out with Phenix.Refine to arrive at the final model with an *R*_*free*_ of 20.9%.

Data collection, anisotropy correction and refinement statistics are given in Table 1.

#### Small-angle X-ray scattering

SAXS analysis was carried out at beamline B21 at Diamond Light Source, Harwell, UK. Samples (45 μL at 3-12 mg/mL) were subjected to size exclusion chromatography on a Shodex KW-403 column in buffer E and subsequent SAXS analysis. The program ScÅtter was used for data analysis (http://www.bioisis.net, Robert P. Rambo, Diamond Light Source). Peak frames were averaged and background-corrected against frames of the same run. Guinier fits were carried out to determine *I*(0) and *R*_*g*_ by truncating the data at the low *q* range to satisfy the *q* x *R*_*g*_ requirement of > 1.30. Guinier plots were linear and confirmed the absence of aggregates. Data from ∼0.013 Å^-1^ < *q* < ∼0.22 Å^-1^ extrapolated to zero angle were used for real space analysis. *D*_*max*_ was fit manually to the *P*(*r*) distributions to obtain good agreement between real space and reciprocal space derived *I*(0) and *R*_*g*_ values. All samples were measured in duplicate with near identical results. The FoXS webserver (Schneidman-Duhovny et al., 2016) was used for the calculation of a theoretical scattering profile of structures and its fitting to an experimental profile (χ^2^ values are given in the figures). See Table S1 for parameters derived from SAXS data analysis.

#### Fluorescence polarization assays

DUB activity was quantified by fluorescence polarization assays using Ub-KG-TAMRA or Lys48-diUb-FlAsH (Pruneda et al., 2016) substrates. Kinetic experiments were carried out in black, round-bottom, non-binding surface 384-well plates (Greiner) at 25°C in PBS (phosphate-buffered saline, pH 7.4) supplemented with 5 mM DTT and 0.05 mg/mL BSA with 0.1 μM substrate concentration and indicated enzyme concentrations in 20 μL reactions. Data were recorded on a PheraStar plate reader (BMG Labtech), equipped with an optic module using 540 nm/590 nm (TAMRA) and 485 nm/520 nm (FlAsH) filter pairs for excitation and emission, respectively. For TAMRA assays, typically one read per min over 60 min was recorded, whereas for FlAsH assays one read per 20-30 s was recorded to cover the steep decline in polarization at high enzyme concentrations. Polarization values of 50 mP for free KG-TAMRA and 160 mP for Ub-KG-TAMRA were determined in a cuvette-based spectrofluorometer and used as reference before conversion into anisotropies (mA). Data were analyzed using Microsoft Excel and Graphpad Prism and catalytic efficiencies were derived as described previously (Gersch et al., 2017). All experiments were performed with technical triplicates and in at least two independent experiments.

#### Gel-based ubiquitin-chain cleavage assay

Cleavage of Lys48-tetraUb (assembled from wild-type ubiquitin as reported by Michel et al., 2018) was followed by SDS-PAGE and Coomassie staining. 100 μL reactions were set up by mixing 50 μL of USP25 (2x: 0.6 μM) and 50 μL of Lys48-tetraUb (2x: 0.17 mg/mL, 5 μM) in 20 mM Tris pH 8.0, 100 mM sodium chloride and 5 mM DTT. Aliquots were taken at several time points by mixing 20 μL with 5 μL of LDS sample buffer, of which 10 μL were run on a gel. Samples were preequilibrated at 37°C, and reactions were incubated 37°C.

#### SEC-MALS

Size-exclusion multi-angle light scattering (SEC-MALS) analysis was carried out using an Agilent 1200 Series chromatography system coupled to a DAWN Heleos II multi-angle light scattering detector as well as an Optilab rEX refractive index detector (Wyatt Technology). Samples (100 μL of 1-2 mg/mL protein solutions unless noted otherwise) were subjected to size-exclusion chromatography on a Superdex 200 10/300 column run at a flow of 0.5 mL/min in PBS supplemented with 2 mM DTT. Masses and errors were derived from analysis in Astra 6.1 (Wyatt Technology) following calibration with BSA.

#### Fluorescence-detection size-exclusion chromatography

USP28(149-703)∼Ub-PA was buffer-exchanged into PBS on a Superdex 200 10/300 column, concentrated via centrifugation and fluorescently labeled with NHS-functionalized dye NT-495 (MO-L003, Nanotemper, Munich). Free dye was removed by gel filtration into PBS + 2 mM DTT. Comparison of the peak profiles was used to confirme that labeling did not interfere with dimerization. Protein concentration and labeling efficiency (90%) were determined through absorption measurements at 280 nm and 493 nm, respectively. Fluorescence-detection size-exclusion chromatography (F-SEC) analysis was carried out on an Äkta Purifier coupled to an AUC-905 autosampler, a Superdex 200 Increase 3.2/300 column and a Hitachi 5440 FL detector (Excitation: 493 nm, Emission: 521 nm) in PBS + 2 mM DTT running at 0.05 mL/min with 25 μL injections of sample. PMT voltage and averaging time were varied to ensure the dynamic range of the detector was used. Curves were scaled in signal intensity with regard to the integral of the peak according to the injected amount of protein for comparison of all samples on one a.u. scale.

#### Thermal shift assay

Thermal shift assays were carried out on a Corbett RG-6000 real time PCR cycler (30°C to 85°C with 7 s per 0.5°C). Samples contained 4 μM protein and 4x Sypro Orange dye in PBS supplemented with 5 mM DTT. Protein melting curves were obtained as the maxima of d*F*/d*T* versus *T* plots. All data were recorded with 10 technical replicates and were consistent accross two independent experiments.

#### NMR spectroscopy

All NMR data were collected at 298 K using Bruker Avance spectrometers with ^1^H resonance frequencies of 600 or 800 MHz fitted with ^1^H{^13^C,^15^N} triple-resonance cryoprobes with 5% D_2_O added to each sample as a lock solvent. ^1^H-^15^N BEST–TROSY (band selective excitation short transients transverse relaxation optimized spectroscopy) experiments were collected as standard ^1^H,^15^N-2D correlation experiments with an in-house optimized pulse sequence (Favier and Brutscher, 2011). Data used for the overlay of 2D spectra for binding tests were recorded with ^15^N-labeled samples at 75 μM to which equimolar amounts of a different, unlabeled protein were added where indicated. Samples were prepared in PBS + 4 mM DTT. All spectra were processed using the program Topspin 3.1 and analyzed using the program Sparky 3.115.

#### Native PAGE, in-gel GFP fluorescence and immunoblotting analysis

HEK293 cells (ATCC CRL-1573) were maintained in DMEM + GlutaMAX supplemented with 10% (v/v) fetal bovine serum (FBS) and penicillin-streptomycin at 37°C in a humidified atmosphere containing 5% CO_2_. Cells were tested negative for mycoplasma contamination with a Lonza MycoAlert Assay. On day one, cells were seeded in 6 well dishes (0.6 – 1 million cells / well). On day two, cells were transfected with 3 μg vector and 9 μg PEI premixed in 200 μL OPTI-MEM. On day three, cells were washed with ice-cold PBS and lysed in lysis buffer (150 μL/well) containing 50 mM Tris pH 7.5, 150 mM sodium chloride, 2 mM EDTA, 10% (w/v) glycerol, 0.8% (v/v) NP-40, protease inhibitors (Complete EDTA-free protease inhibitor tablets, Roche), 4 mM DTT and 10 mM beta-mercaptoethanol. After incubation for 10 min at 4°C, 150 μL/well lysis buffer supplemented with 10 mM magnesium chloride and 1:1000 benzonase (Merck) were added. Following clearance by centrifugation (14,000 xg, 10 min, 4°C), protein concentrations of the supernatants were measured by Bradford assay and BSA as standard. Whole cell lysate samples for SDS-PAGE analysis were obtained by mixing supernatant and 4x LDS sample buffer.

For native PAGE analysis, lysates were mixed 1:1 with 2x native PAGE sample buffer (200 mM Tris pH 8.6, 20% (v/v) glycerol, 0.005% (w/v) bromophenol blue) and promptly analyzed. Proteins were separated on NuPAGE 3%–8% Tris-Acetate protein gels (1.0 mm, ThermoFisher) with 25 mM Tris, 192 mM glycine, pH 8.2-8.4 as running buffer at constant 150 V for 110 min at room temperature. For the measurement of in-gel GFP fluorescence, gels were imaged on an Amersham Typhoon Biomolecular Imager using the Cy2 channel (GE Healthcare). For analysis by immunoblotting, gels were incubated for 10 min in native PAGE running buffer supplemented with 0.1% (w/v) SDS with gentle agitation. Proteins were then transferred to a nitrocellulose membrane using the Trans-Blot Turbo system (Bio-Rad). Membranes were blocked in a 5% (w/v) milk solution in PBS-T (PBS + 0.1% (v/v) Tween-20) for 20 min and incubated for 1 h at room temperature with a primary antibody recognizing either USP25 (abcam, ab187156, 1:1000), Flag (Sigma, F3165, 1:2000), USP28 (proteintech, 17707-1-AP, 1:1000), HA (BioLegend, 16B12, 1:1000), GFP (abcam, ab6673, 1:1000), GAPDH (Ambion, AM4300, 1:10000) or Tubulin (Sigma, T6199, 1:4000). All antibodies were used in 3% (w/v) BSA in PBS-T with 0.02% (w/v) sodium azide. Membranes were subsequently incubated with donkey anti-rabbit IgG-HRP (Sigma, GENA934, 1:5000), sheep anti-mouse IgG-HRP (Sigma, GENXA931, 1:5000) or donkey anti-goat IgG-HRP (Santa Cruz Biotech, sc-2020, 1:5000) in blocking solution for 1 h at room temperature. Blots were developed with Clarity Western ECL substrate (Bio-Rad) or Amersham Western Blotting Detection Reagent (GE Healthcare), and imaged using a ChemiDoc MP Imaging System (Bio-Rad).

As a customized size marker, we used recombinant full-length human USP25 analyzed on a HiLoad 16/600 Superdex 200 pg column in buffer E immediately before the experiment. Fractions corresponding to the tetrameric and dimeric oligomerization states (see Figure S6A) were concentrated to 1.0 mg/mL, and samples were prepared as described above for native PAGE analysis. Blots were stained by Ponceau S solution after the transfer and imaged, followed by the blocking step. Ponceau S images and immunoblots were aligned based on the membrane contours (see Figure S8).

For co-transfection experiments, cells were transfected with 0.6 μg/well of Flag-LSD1 or HA-TNKS2 vector, and up to 3 μg/well of vector of USP28 or USP25, respectively, topped up to 3 μg with empty vector where appropriate. Samples were harvested as described above and analyzed by SDS-PAGE and immunoblotting.

### Quantification and Statistical Analysis

Statistical details of the experiments can be found in the figure legends and in the figures. Data are given as mean ± standard deviation (SD) or mean ± standard error (SEM) as defined in the legend.

### Data and Software Availability

Protein structures have been deposited with the protein data bank under PDB: 6HEH, 6HEI, 6HEJ, 6HEK, 6HEL, 6HEM.

## Supplemental Information

Document S1. Figures S1–S8 and Table S1

Document S2. Article plus Supplemental Information
