# Supplementary material for: Distinct USP25 and USP28 Oligomerization States Regulate Deubiquitinating Activity
Source: Mol Cell. 2019 May 2;74(3):436–451.e7. doi: 10.1016/j.molcel.2019.02.030 (PMC6509359; doi:10.1016/j.molcel.2019.02.030)
Supplement: Document S1. Figures S1–S8 and Table S1 [file mmc1.pdf]

Molecular Cell, Volume *74*

Supplemental Information

Distinct USP25 and USP28 Oligomerization States Regulate Deubiquitinating Activity

Malte Gersch, Jane L. Wagstaff, Angela V. Toms, Bradford Graves, Stefan M.V. Freund, and David Komander

Supplemental Figure 1

A

USP DUBs			identity	similarity
USP12	USP46		88%	96%
USP18	USP41		79%	83%
USP5	USP13		58%	82%
USP44	USP49		58%	78%
USP20	USP33		56%	78%
**USP25**	**USP28**		**48%**	**76%**
USP6	USP32		56%	68%
USP16	USP45		36%	66%
USP35	USP38		40%	66%
USP9X	USP24		27%	58%
USP2	USP21		35%	57%
USP26	USP29	USP37	29%	56%
USP4	USP11	USP15	33%	56%
USP31	USP43		33%	54%
USP36	USP42		28%	49%
USP22	USP27	USP51	39%	49%

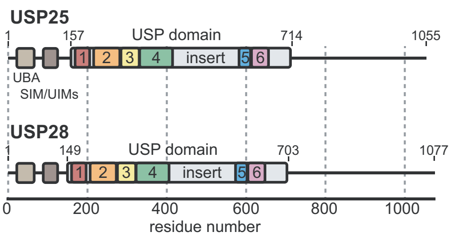

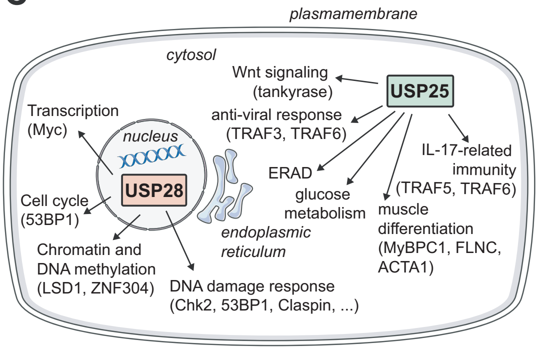

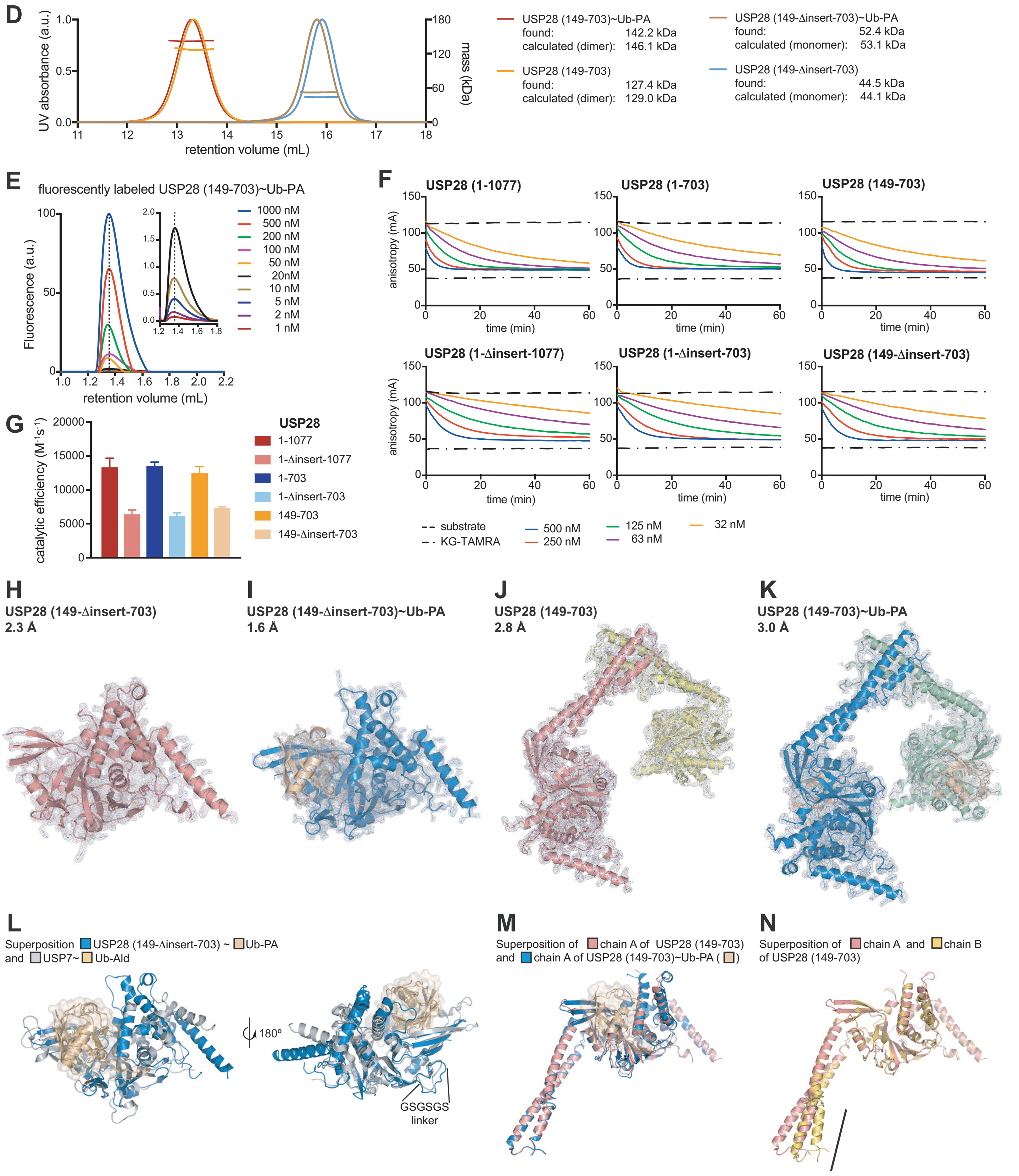

Figure S1. Cellular roles for USP25 and USP28. Biochemical and structural analysis of human USP28 proteins. Related to Figure 1.

(A) List of pairs or triplets of highly homologous human USPs DUBs. The percentage of identical residues as well as identical and similar residues is given. Data are based on alignments of full length protein sequences carried out with the Uniprot webserver. USP25 and USP28 are highlighted in bold.

(B) Schematic representation of human USP25 and USP28 constructs. The catalytic domain is shown in grey, an N-terminal UBA domain, SUMO-interaction motif (SIM) and ubiquitin-interaction motifs (UIMs) are shown in brown. Colored boxes indicate the conserved boxes characteristic of USP domains (Ye et al., 2009).

(C) Previously identified cellular roles linked to USP25 and USP28 indicated by arrows. Subcellular compartments and structures labeled in italics.

(D) SEC-MALS analysis of indicated constructs either in the apo form or bound to Ub-PA. Data of samples in the apo form are as shown in Figure 1C for easy comparison. Identified masses are matched to either monomeric or dimeric expected masses.

(E) A dilution series of a fluorescently labeled USP28 (149-703)~Ub-PA protein sample was analyzed on a size-exclusion chromatography system fitted with a fluorescence detector. Data collected on a more sensitive detector setting are shown as an inlet figure. The same central peak position is indicated with dotted lines in both graphs. The given concentrations correspond to the injected sample concentrations.

(F) Representative fluorescence anisotropy-time traces of Ub-KG-TAMRA cleavage assays from averaged technical triplicates of indicated USP28 constructs and concentrations.

(G) Catalytic activities of USP28 constructs determined from data shown in F. Data are shown as mean ± standard deviation from 2-5 independent experiments, each collected from technical triplicates.

(H, I, J and K) Overall views of the asymmetric units of indicated crystal structures (see Figure 1D-G for cartoon representations). Residues are shown as lines and in cartoon representation. Overlaid electron density maps in grey correspond to weighted $2|F_O|–|F_C|$ electron density contoured at 1 σ.

(L) Superposition of the Ub-PA-bound structure of USP28 (149-∆insert-703) and USP7 bound to ubiquitin-aldehyde (PDB-ID: 1NBF).

(M) Superposition of the apo and Ub-PA-bound structures of USP28 (149-703).

(N) Superposition of the two chains forming the asymmetric unit of the apo structure of USP28 (149-703). Notable differences in the insertion sequence are highlighted with a black line.

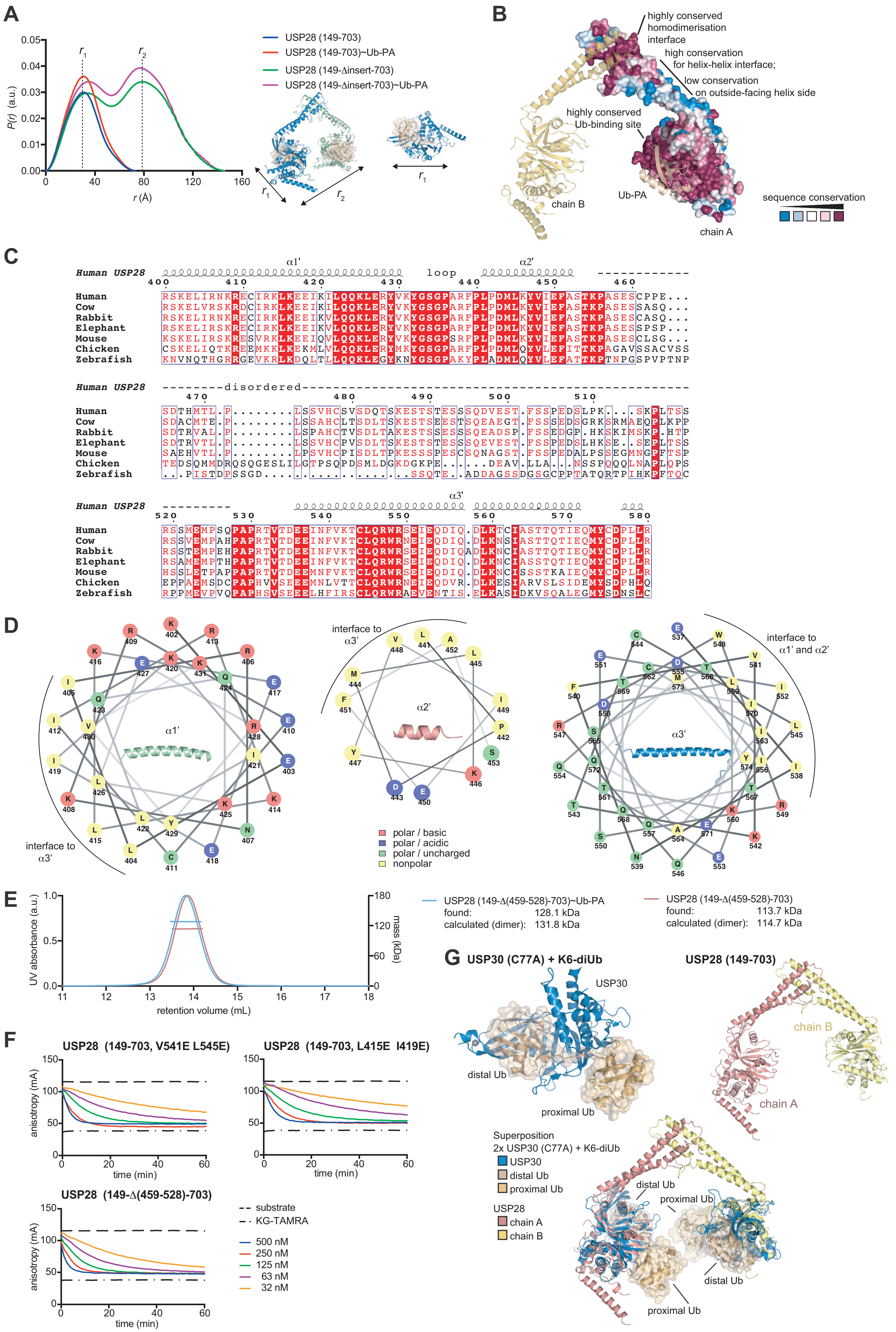
A
P(r) (a.u.)
r (Å)
r_1
r_2
USP28 (149-703)
USP28 (149-703)~Ub-PA
USP28 (149-Δinsert-703)
USP28 (149-Δinsert-703)~Ub-PA
B
highly conserved homodimerisation interface
high conservation for helix-helix interface; low conservation on outside-facing helix side
highly conserved Ub-binding site
chain B
Ub-PA
chain A
sequence conservation
C
Human USP28
α1'
loop
α2'
Human RSKELIRNKRECIRKLKEEIKILQQKLERYVKYGSGPARFPLPDMLKYVIEFASTKPASESCPPE...
Cow RSKELVRSKRDCIRKLKEEIKILQQKLERYVKYGSGPARFPLPDMLKYVIEFASTKPASESSASQ...
Rabbit RSKELIRNKRECIRKLKEEIKVLQQKLERYVKYGSGPARFPLPDMLKYVIEFASTKPASESCASQ...
Elephant RSKELIRSKRECIRKLKEEIKVLQQKLERYVKYGSGPARFPLPDMLKYVIEFASTKPASESSPSP...
Mouse KSKELIRSKRESVRKLKEEIQVLQQKLERYVKYGSGPSRFPLPDMLKYVIEFASTKPASESCLSG...
Chicken CSKELIQTKREEMKKLKEKMLVLQQKLERYMKYGSGPARFPLPDMLQYVLEFITTKPAGAVSSACVSS
Zebrafish KNVNQTHGRRGEVKRLKDQLTLLQQKLEGYKNYGSGPAKYPLADMLQYVLEFATTKPTNPGSPVPTNP
Human USP28
disordered
Human SDTHMTL.P........LSSVHCSVSDQTSKESTSTESSSQDVEST.FSSPEDSLPK...SKPLTSS
Cow SDACMTE.P........LSSAHCLTSDLTSKESTSEESTSQEAEGT.FSSSEDSGRKSRMAEQPLKPP
Rabbit SDTRVAL.P........LSPAHCTVSDLTAKESTSSESSSQEADSP.FSSEDGP.HKSKIMSKP.HTP
Elephant SDTRVTL.P........LSSVHCPVSDLTSKESTSTESSSQEVEST.FSSPEDSLHKSE..SEPLTSS
Mouse SAEHVTL.P........LPSVHCPISDLTPKESSSPESCSQNAGST.FSSPEDALPSSEGMNGPFTSP
Chicken TEDSQMMDRQSQGESLILGTPSQPDSMLDGKDGKPE.....DEAV.LLA...NSSPQQQLNAPLQPS
Human USP28
α3'
Human RSSMEMPSQPAPRTVTDEEINFVKTCLQRWRSEIEQDIQ.DLKTCIASTTQTIEQMYCDPLLR
Cow RSSVEMPAHPAPRTVTDEEINFVKTCLQRWRNEIEQDIQ.DLKNSIASTTQTIEQMYCDPLLR
Rabbit RSSTEMPEHPAPRTVTDEEINFVKTCLQRWRSEIEQDIQADLKNCIASTTQTIEQMYCDPLLR
Elephant RSAMEMPTHPAPRTVTDEEINFVKTCLQRWRSEIEQDIQ.DLKNCIASSTQTIEQMYCDPLLR
Mouse HSSLETPAPPAPRTVTDEEMNFVKTCLQRWRSEIEQDIQ.DLKNCISSSTKAIEQMYCDPLLR
Chicken EPPAEMSDCPAPHVVSEEEMNLVTTCLQRWRNEIEQDVR.DLKESIARVSLSIDEMYSDPHLQ
Zebrafish RPPMEVPVQPAPHSVSEEELHFIRSCLQRWRAEVENTIS.ELKASIDKVSQALEGMYSDNSLC
D
α1'
interface to α3'
α2'
α3'
interface to α1' and α2'
polar / basic
polar / acidic
polar / uncharged
nonpolar
E
UV absorbance (a.u.)
mass (kDa)
retention volume (mL)
USP28 (149-Δ(459-528)-703)~Ub-PA
found: 128.1 kDa
calculated (dimer): 131.8 kDa
USP28 (149-Δ(459-528)-703)
found: 113.7 kDa
calculated (dimer): 114.7 kDa
F
USP28 (149-703, V541E L545E)
USP28 (149-703, L415E I419E)
USP28 (149-Δ(459-528)-703)
anisotropy (mA)
time (min)
substrate
KG-TAMRA
500 nM
250 nM
125 nM
63 nM
32 nM
G
USP30 (C77A) + K6-diUb
USP30
distal Ub
proximal Ub
USP28 (149-703)
chain B
chain A
Superposition
2x USP30 (C77A) + K6-diUb
USP30
distal Ub
proximal Ub
USP28
chain A
chain B

Figure S2. Structural analysis of the USP28 insertion sequences. Related to Figure 2.

(A) Pair-wise distance distribution functions *P(r)* calculated from SAXS data shown in Figure 2A for indicated constructs. The most frequent distances are highlighted with dashed lines and labeled r_1 and r_2. These are similar to the radius of gyration of the isolated USP domain (r_1) as well as the distance between the two USP domains in the dimer (r_2).

(B) Sequence conservation calculated from 96 USP28 protein sequences annotated as USP28 in the Uniprot database mapped as colored surface on chain A of the USP28 (149-703) structure. Chain B as well as the position of Ub-PA of the USP28 (149-Δinsert-703)~Ub-PA complex structure are shown as semi-transparent cartoons.

(C) Sequence alignment of representative USP28 members of vertebrate families for the insertion sequence. Secondary structure and disorder assignments were carried out with respect to the USP28 (149-703) structure.

(D) Helical wheel diagrams for the three helices α1’, α2’ and α3’ of the USP28 insertion generated with the NetWheels webserver. Residues are colored according to their chemical nature as indicated. Interfaces are labeled and highlighted with black arcs. Cartoon representation of the respective helices as shown in Figure 2C.

(E) SEC-MALS analysis of USP28 catalytic domain constructs where the disordered part of the insertion is deleted (149-Δ(459-528)-703).

(F) Representative fluorescence anisotropy-time traces of Ub-KG-TAMRA cleavage assays from averaged technical triplicates of indicated USP28 constructs and concentrations. Catalytic efficiencies are reported in Figure 2E.

(G) Cartoon representation of inactive USP30 bound to K6-linked diUb (PDB-ID: 5OHP), with distal and proximal ubiquitin moieties labeled and also shown as transparent surfaces (*top left*). Cartoon representation of USP28 (149-703) (*top right*). Superposition of two copies of the USP30 complex on each of the catalytic domains of USP28 (*bottom*).

Supplemental Figure 3

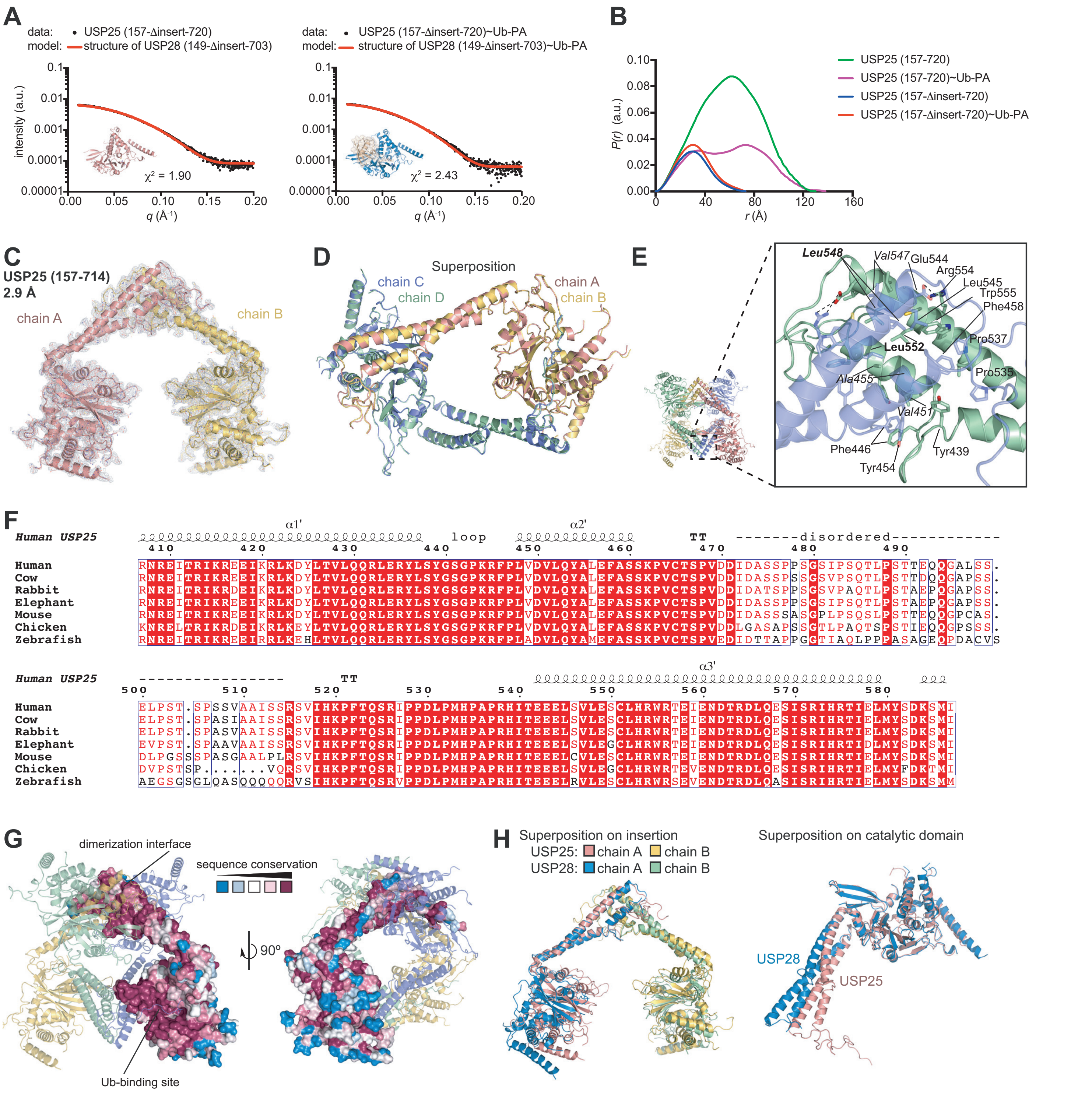

Figure S3. Structural and biochemical analysis of the catalytic domain of USP25. Related to Figure 3.

(A) Small angle X-ray scattering (SAXS) data shown as black dots which was collected from indicated USP25 protein samples. Expected scattering curves are shown in red that were calculated from solved USP28 X-ray crystal structures shown as small inlets.

(B) Pair-wise distance distribution functions *P(r)* calculated from SAXS data shown in Figures 3C and S3A for indicated constructs.

(C) Overall views of the asymmetric unit of the USP25 crystal structure. Residues are shown as lines and in cartoon representation. The overlaid electron density map in grey corresponds to weighted $2|F_O|–|F_C|$ electron density contoured at 1 σ.

(D) Superposition of chain A on top of chain B forming one asymmetric unit. A superposition of symmetry-related chains C (corresponding to A) and D (corresponding to B) is also shown.

(E) Close-up view of the indicated dimerization interface of USP25 chains C and D (compare to Figure 2B on the right for the respective USP28 interaction). Relevant residues are shown as sticks. Residues mutated to generate monomeric USP25 are labeled in bold, residues characteristically different from the respective USP28 interface are labeled in italics.

(F) Sequence alignment of representative USP25 members of vertebrate families for the insertion sequence. Secondary structure and disorder assignments were carried out with respect to the USP25 catalytic domain structure.

(G) Sequence conservation calculated from USP25 protein sequences mapped as colored surface on chain A of the USP25 (157-714) structure in two orientations. Chains B, C and D are shown as semi-transparent cartoons.

(H) Superpositions of cartoon representations of the crystal structures of USP25 and USP28. Shown are an alignment of dimeric forms (*left*) and individual catalytic domains (*right*).

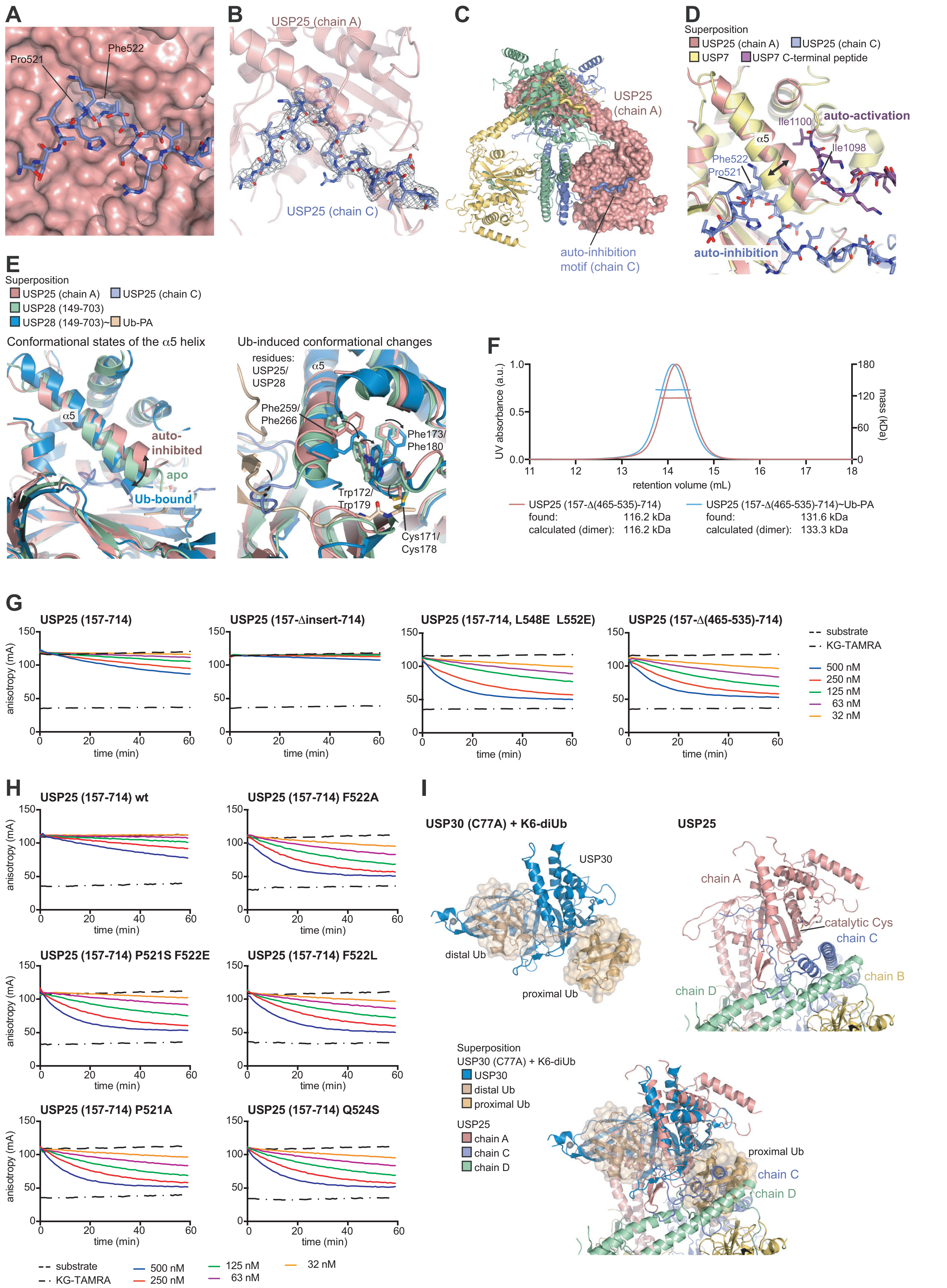

A
Pro521
Phe522
B
USP25 (chain A)
USP25 (chain C)
C
USP25 (chain A)
auto-inhibition motif (chain C)
D
Superposition
USP25 (chain A)
USP25 (chain C)
USP7
USP7 C-terminal peptide
Ile1100
auto-activation
α5
Ile1098
Phe522
Pro521
auto-inhibition
E
Superposition
USP25 (chain A)
USP25 (chain C)
USP28 (149-703)
USP28 (149-703)~Ub-PA
Conformational states of the α5 helix
α5
auto-inhibited
apo
Ub-bound
Ub-induced conformational changes
residues: USP25/ USP28
α5
Phe259/ Phe266
Phe173/ Phe180
Trp172/ Trp179
Cys171/ Cys178
F
UV absorbance (a.u.)
mass (kDa)
retention volume (mL)
USP25 (157-Δ(465-535)-714)
found: 116.2 kDa
calculated (dimer): 116.2 kDa
USP25 (157-Δ(465-535)-714)~Ub-PA
found: 131.6 kDa
calculated (dimer): 133.3 kDa
G
USP25 (157-714)
USP25 (157-Δinsert-714)
USP25 (157-714, L548E L552E)
USP25 (157-Δ(465-535)-714)
anisotropy (mA)
time (min)
substrate
KG-TAMRA
500 nM
250 nM
125 nM
63 nM
32 nM
H
USP25 (157-714) wt
USP25 (157-714) F522A
USP25 (157-714) P521S F522E
USP25 (157-714) F522L
USP25 (157-714) P521A
USP25 (157-714) Q524S
I
USP30 (C77A) + K6-diUb
USP30
distal Ub
proximal Ub
USP25
chain A
catalytic Cys
chain C
chain B
chain D
Superposition
USP30 (C77A) + K6-diUb
USP30
distal Ub
proximal Ub
USP25
chain A
chain C
chain D

Figure S4. Mechanism of conserved auto-inhibition through oligomerization in USP25. Related to Figure 4.

(A) Close-up view of the pocket within the catalytic domain of USP25 (shown as red surface) that engages the auto-inhibition motif (shown as blue sticks).

(B) Electron density map in grey of the autoinhibition-motif contoured at 0.8 σ corresponding to weighted $2|F_O|-|F_C|$ electron density. The USP25 catalytic domain is shown as a semitransparent cartoon.

(C) Cartoon representation of tetrameric USP25 as in Figure 3D. Chain A is shown as a surface to highlight binding of the AIM of chain C to the catalytic domain of chain A.

(D) Superposition of cartoon representations of USP25 (red) and USP7 (yellow, PDB-ID: 5JTJ) in complex with the auto-inhibition motif (blue) and the USP7 C-terminal activating peptide (purple), respectively. Their binding to opposite sites of the α5 helices of the catalytic domains and the diverging α5 conformations are highlighted by a black arrow. Key hydrophobic residues in both peptidic motifs are labeled.

(E) Superposition of indicated crystal structures in cartoon representation showing the different positioning of the α5 helix in the catalytic USP domains depending on auto-inhibition or ubiquitin binding (*left*). A putative conformational relay mechanism links the positioning of the α5 helix through the sidechain conformation of Phe259 (USP25) / Phe266 (USP28) to the catalytic cysteine and its following aromatic residues (*right*). Conformational changes are highlighted with black arrows. The cartoon representation of ubiquitin is not shown in the left panel for clarity.

(F) SEC-MALS analysis of USP25 constructs in which the sequence corresponding to the part that is disordered in USP28 is deleted (construct corresponds to USP28 (149-Δ(459-528)-703), see Figure S2F).

(G and H) Representative fluorescence anisotropy-time traces of Ub-KG-TAMRA cleavage assays of indicated USP25 proteins and concentrations. Catalytic efficiencies are shown in Figure 4D and 4F.

(I) Cartoon representation of inactive USP30 bound to K6-linked diubiquitin (PDB-ID: 5OHP), with distal and proximal ubiquitin moieties labeled and also shown as transparent surfaces (*top left*). Cartoon representation of USP25 (157-714) (*top right*). Superposition of both structures on the USP domain of chain A of USP25,

showing the clashing of the proximal ubiquitin moiety in its putative position with chains C and D from the second USP25 dimer (*bottom*).

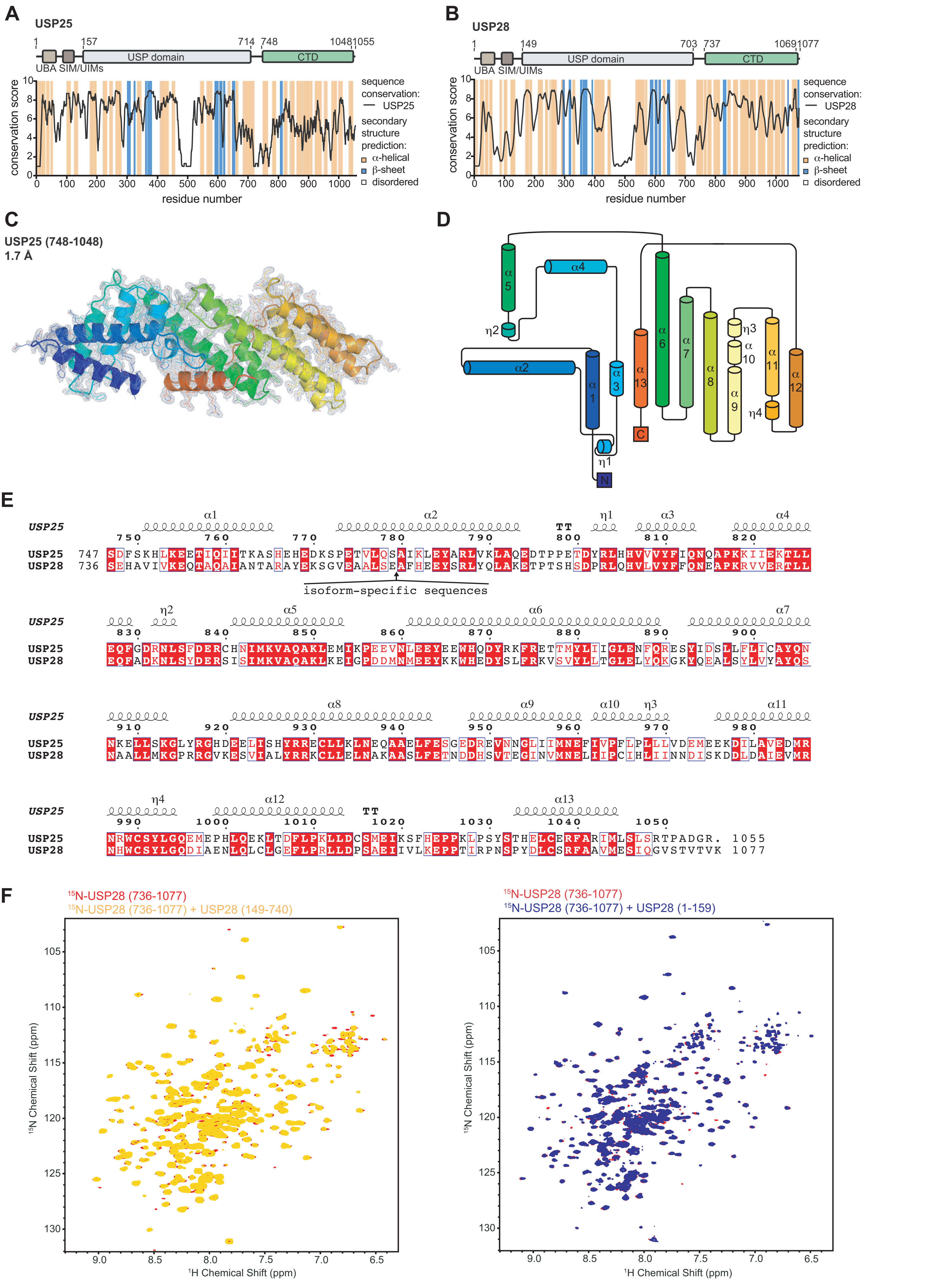
A
USP25
1 157 714 748 1048 1055
UBA SIM/UIMs
USP domain
CTD
conservation score
residue number
sequence conservation:
USP25
secondary structure prediction:
α-helical
β-sheet
disordered
B
USP28
1 149 703 737 1069 1077
UBA SIM/UIMs
USP domain
CTD
conservation score
residue number
sequence conservation:
USP28
secondary structure prediction:
α-helical
β-sheet
disordered
C
USP25 (748-1048)
1.7 Å
D
α4
α5
η2
α2
α1
α3
η1
N
α13
C
α6
α7
α8
η3
α10
α9
α11
η4
α12
E
USP25 747 SDFSKHLKEETIQIITKASHEHEDKSPETVLQSAIKLEYARLVKLAQEDTPPETDYRLHHVVVYFIQNQAPKKIIEKTLL
USP28 736 SEHAVIVKEQTAQAIANTARAYEKSGVEAALSEAFHEEYSRLYQLAKETPTSHSDPRLQHVLVYFFQNEAPKRVVERTLL
isoform-specific sequences
USP25 EQFGDRNLSFDERCHNIMKVAQAKLEMIKPEEVNLEEYEEWHQDYRKFRETTMYLIIGLENFQRESYIDSLLFLICAYQN
USP28 EQFADKNLSYDERSISIMKVAQAKLKEIGPDDMNMEEYKKWHEDYSLFRKVSVYLLTGLELYQKGKYQEALSYLVYAYQS
USP25 NKELLSKGLYRGHDEELISHYRRECLLKLNEQAAELFESGEDREVNNGLIIMNEFIVPFLPLLVDEMEEKDILAVEDMR
USP28 NAALLMKGPRRGVKESVIALYRRKCLLELNAKAASLFETNDDHSVTEGINVMNELIIPCIHLIINNDISKDDLDAIEVMR
USP25 NRWCSYLGQEMEPHLQEKLTDFLPKLLDCSMEIKSFHEPPKLPSYSTHELCERFARIMLSLSRTPADGR. 1055
USP28 NHWCSYLGQDIAENLQLCLGEFLPRLLDPSAEIIVLKEPPTIRPNSPYDLCSRFAAVMESIQGVSTVTVK 1077
F
15N-USP28 (736-1077)
15N-USP28 (736-1077) + USP28 (149-740)
15N-USP28 (736-1077)
15N-USP28 (736-1077) + USP28 (1-159)
15N Chemical Shift (ppm)
1H Chemical Shift (ppm)

Figure S5. Structural analysis of the C-terminal domains of USP25 and USP28. Related to Figure 5.

(A) Sequence conservation of USP25 plotted as a rolling window average in black of scores obtained from the ConSurf webserver. Secondary structure prediction of human USP25 from the Concord webserver indicated as background color. Schematic representation of the domain architecture of USP25 above the graph including the newly identified C-terminal domain.

(B) Analysis as in (A) for USP28.

(C) Overall view of the asymmetric unit of the crystal structures of USP25 (748-1048). Residues are shown as lines and in cartoon representation. The overlaid electron density map in grey corresponds to weighted $2|F_O|-|F_C|$ electron density contoured at 1 σ.

(D) Topology diagram of the USP25 (748-1048) structure generated with the Pro-origami webserver. α helices and 3_{10} helices (η) are consecutively numbered.

(E) Sequence alignment of the C-terminal regions of human USP25 and USP28 with secondary structure elements indicated according to the USP25 (748-1048) structure. The insertion point where sequences of varying length are added to both USP25 and USP28 in isoforms lies within α2 of the CUEL domain. These sequences at least for USP25 customize the function of the DUB by facilitating different protein-protein interactions (Bosch-Comas et al., 2006). With large parts of the inserted sequences being predicted to be disordered, it is unknown how these would structurally remodel the C-terminal region of USP25 and USP28.

(F) ^{1}H-^{15}N BEST-TROSY NMR spectra showing the ^{15}N-labeled C-terminal domain of USP28 (736-1077) (red) overlaid with ^{15}N-labeled USP28 (736-1077) in the presence of unlabeled USP28 (149-740) (yellow, *left*) or unlabeled USP28 (1-159) (blue, *right*). Labeled proteins were at 75 µM and unlabeled proteins were added to equimolar amounts.

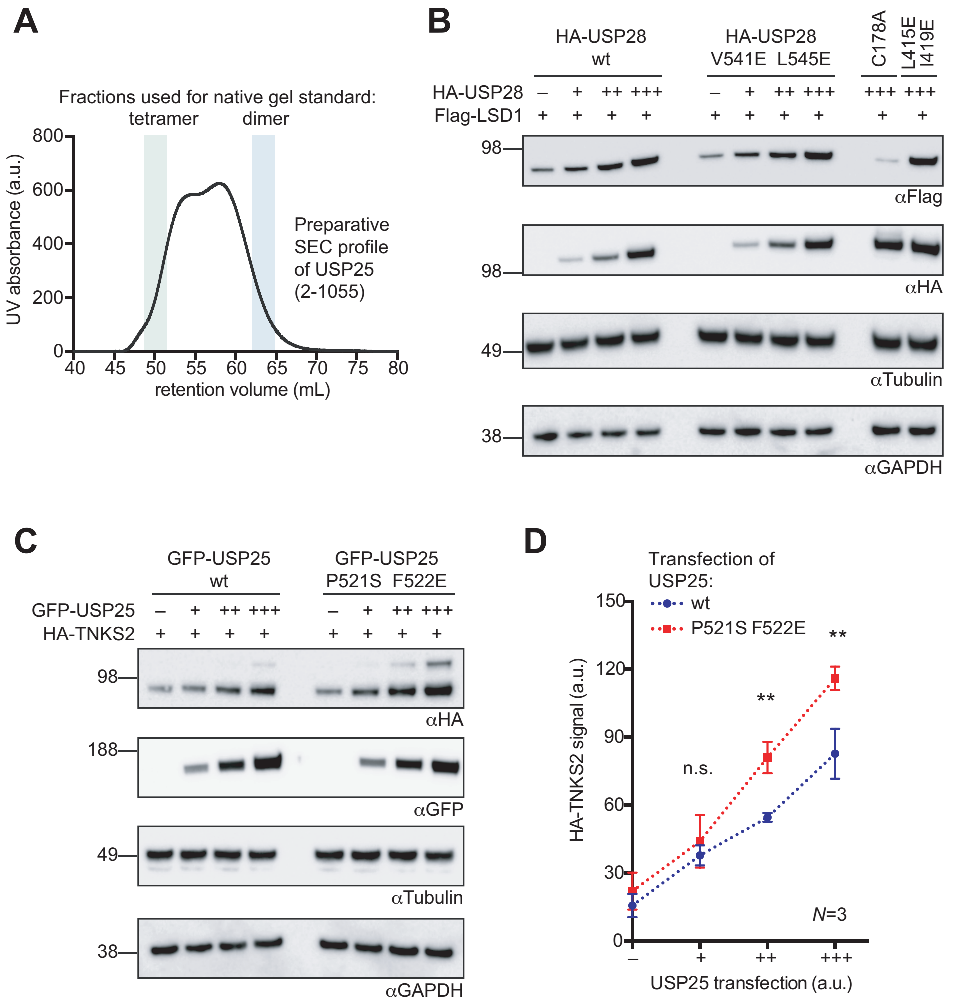
A
Fractions used for native gel standard:
tetramer
dimer
UV absorbance (a.u.)
retention volume (mL)
Preparative SEC profile of USP25 (2-1055)
B
HA-USP28 wt
HA-USP28 V541E L545E
C178A
L415E I419E
HA-USP28
Flag-LSD1
αFlag
αHA
αTubulin
αGAPDH
C
GFP-USP25 wt
GFP-USP25 P521S F522E
GFP-USP25
HA-TNKS2
αHA
αGFP
αTubulin
αGAPDH
D
Transfection of USP25:
wt
P521S F522E
HA-TNKS2 signal (a.u.)
n.s.
**
N=3
USP25 transfection (a.u.)

Figure S6. Functional assessment of oligomerization states of USP25 and USP28. Related to Figure 6.

(A) Size-exclusion chromatogram of full-length human USP25. Fractions used as size standards for native PAGE analysis are indicated with colors.

(B) Immunoblotting for indicated epitopes in lysates from HEK-293 cells transfected with vectors for the expression of Flag-LSD1 and increasing amounts of vector for HA-tagged USP28 proteins.

(C) Experiment as in B with HA-TNKS2 as substrate and increasing amounts of vector of GFP-tagged USP25 constructs. Since transfection of identical vector amounts of USP25 (P521S F522E) and USP25 wt led to a larger amount of protein of USP25 (P521S F522E), presumably due to its higher activity and increased auto-deubiquitination, the amounts of transfected vector were adjusted to achieve similar cellular protein concentrations.

(D) Quantification of the HA-TNKS2 immunoblotting signal from three independent repeats of the experiment shown in C. Data are shown as mean ± standard deviation (**: $p < 0.01$, two-tailed *t*-test; n.s., not significant).

Supplemental Figure 7

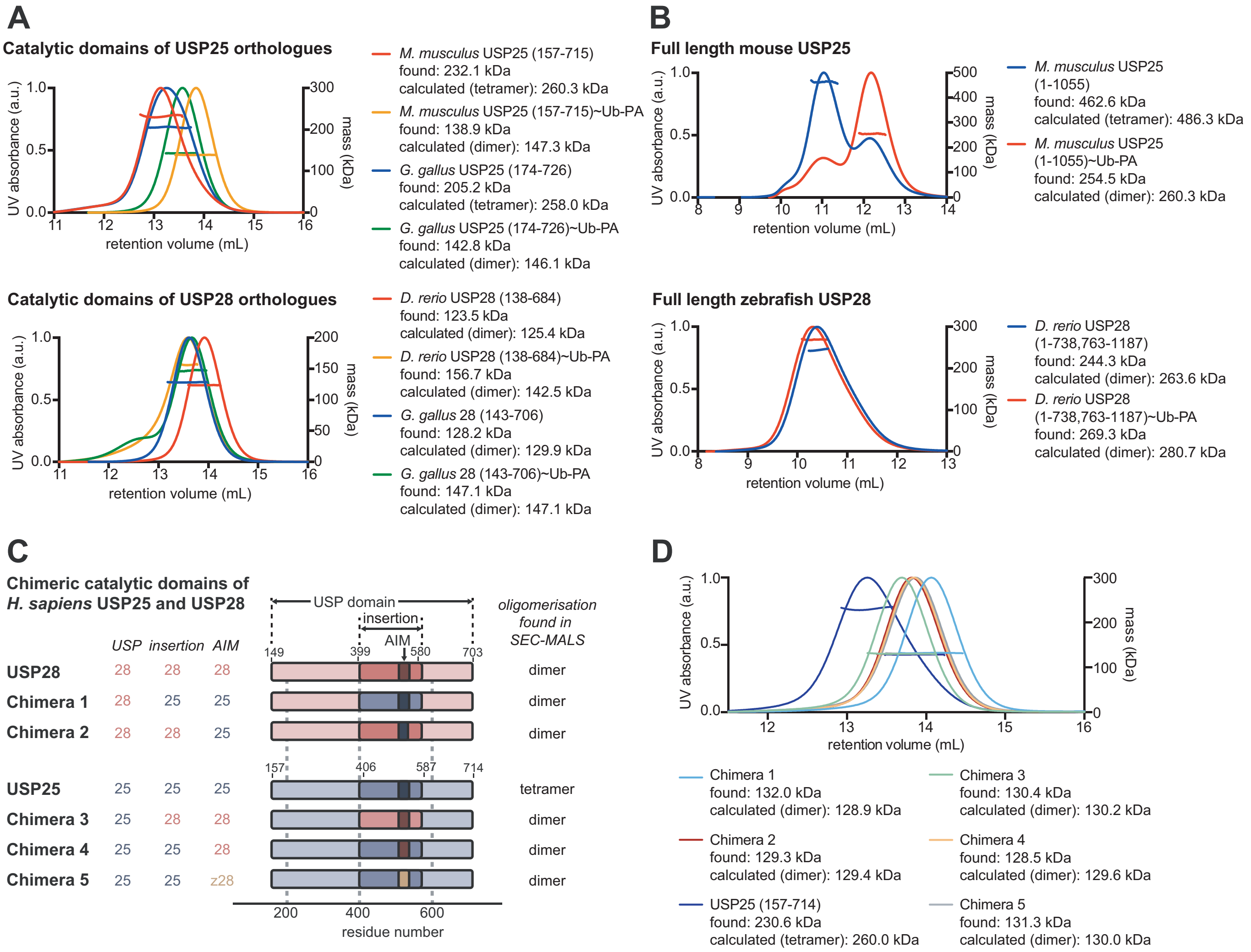

Figure S7. Molecular requirements for distinct oligomerization states of USP28 and USP25 catalytic domains. Related to Figure 7.

(A) SEC-MALS analysis of catalytic domains of USP28 orthologues (*top*) and catalytic domains of USP25 orthologues (*bottom*) both in their apo form and bound to Ub-PA. Identified masses are matched to either tetrameric or dimeric expected masses.

(B) SEC-MALS analysis of full length mouse USP25 (*top*) and full-length zebrafish USP28 (*bottom*) both in their apo form and bound to Ub-PA. Identified masses are matched to either tetrameric or dimeric expected masses.

(C) Schematic representation of chimeric catalytic domain constructs of human USP25 and USP28 proteins. Grafting of sequences was performed as indicated, based on equivalent positions identified from a sequence alignment and from superposition of structures (insertion of USP28: Y399-R580, "AIM"-equivalent sequence: S508-Q527; insertion of USP25: H406-I587, AIM: 515-534). In chimera 5, the AIM of human USP25 was replaced with the equivalent sequence of *D. rerio* USP28. The zebrafish USP28 sequence shares the central IHKPFTQ motif with the AIM of human USP25 (see Figure 4B), but contains different amino acids flanking this motif on both sides. These changes are sufficient to prevent tetramerization (see SEC-MALS data in B and D).

(D) SEC-MALS analysis of chimeric catalytic domain constructs shown in C and tetrameric USP25 as control. Identified masses are matched to either tetrameric or dimeric expected masses. Observed oligomerization states are listed in C.

Uncropped blots and gels

Supplemental Figure 8

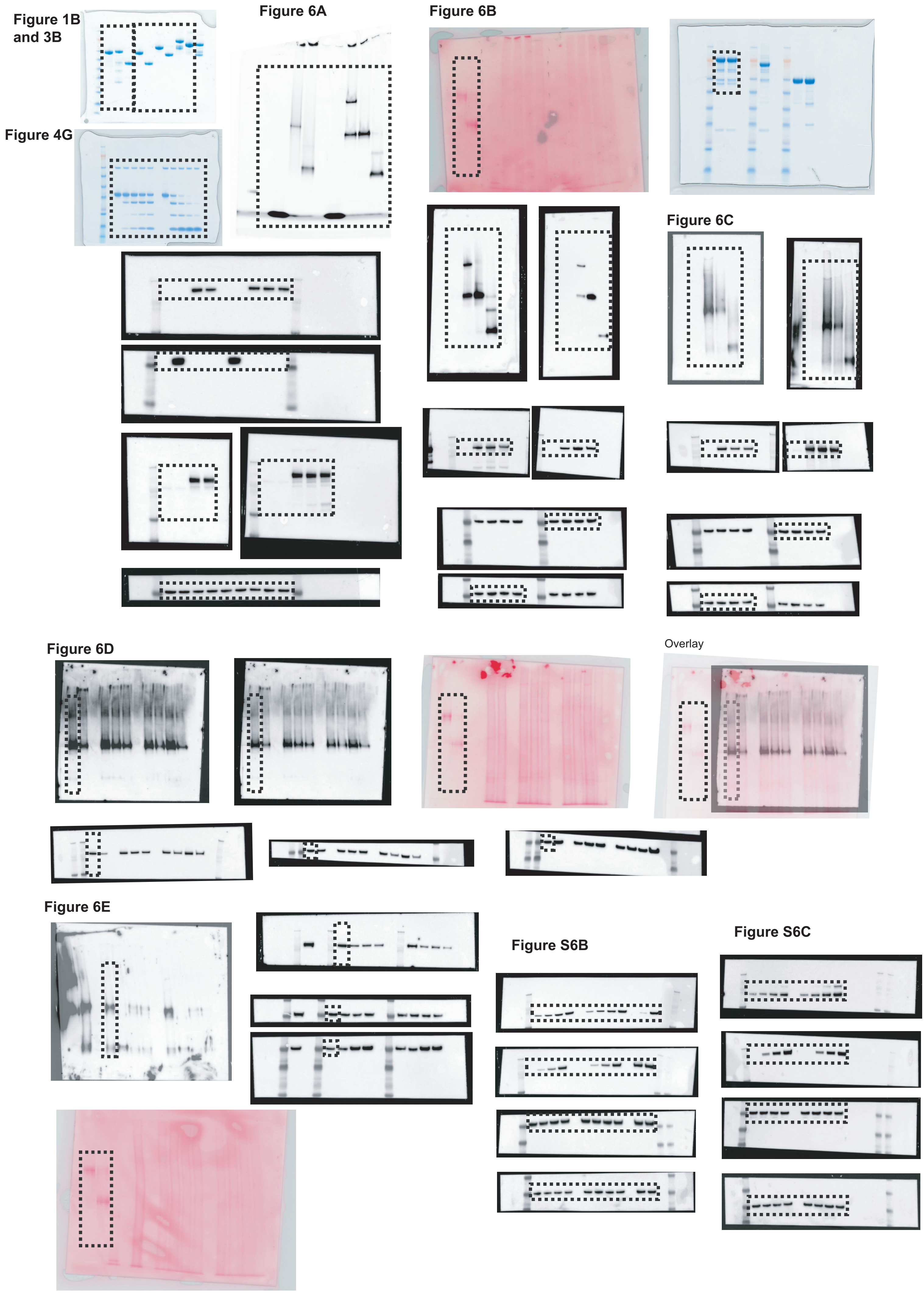

Figure S8. Uncropped gels and blots. Related to Figures 1, 3, 4, 6 and S6.

Supplemental Table 1. Parameters derived from SAXS data analysis. R_g denotes the radius of gyration obtained from Guinier plot fitting, D_{max} and R_{ave} denote the maximum distance and the average radius, respectively, obtained from real space data analysis. Related to Figures 2A, 3C and S3A.

**Sample**	**R_g (Å)**	**D_{max} (Å)**	**R_{ave} (Å)**	**Peaks in *P(r)* (Å)**
USP28 149 -703 apo	50.6	145	65.1	33.8, 79.0
USP28 149-703~Ub-PA	51.4	146	64.3	35.3, 76.6
USP28 149-Δinsert-703 apo	24.1	73	30.9	30.8
USP28 149-Δinsert-703~Ub-PA	24.1	73	31.5	30.8
USP25 157-720 apo	45.8	130	60.5	61.8
USP25 157-720~Ub-PA	46.6	138	60.3	34.0, 73.8
USP25 157-Δinsert-720 apo	24.2	73	31.0	30.0
USP25 157-Δinsert-720~Ub-PA	24.9	73	31.8	30.4
