# Supplementary material for: Distinct USP25 and USP28 Oligomerization States Regulate Deubiquitinating Activity
Source: Mol Cell. 2019 May 2;74(3):436–451.e7. doi: 10.1016/j.molcel.2019.02.030 (PMC6509359; doi:10.1016/j.molcel.2019.02.030)
Supplement: Document S2. Article plus Supplemental Information [file mmc2.pdf]

Article

Molecular Cell

Distinct USP25 and USP28 Oligomerization States Regulate Deubiquitinating Activity

Graphical Abstract

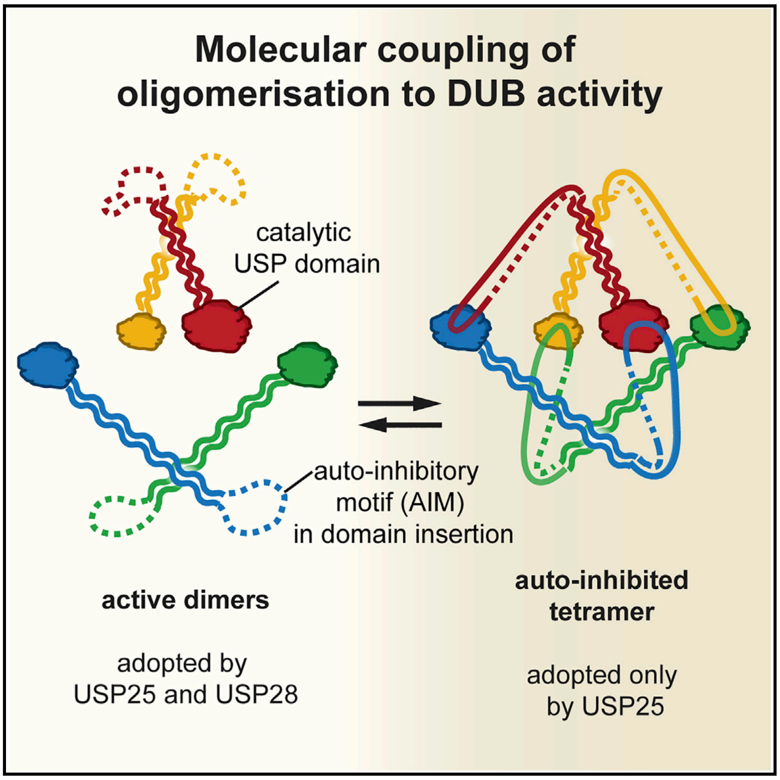

Highlights

- Insertions in USP25 and USP28 catalytic domains mediate their oligomerization
- DUB activity of USP25, but not USP28, is inhibited by tetramerization
- An autoinhibitory motif in USP25 blocks Ub binding, impairing its activity
- Dimers of USP28, as well as dimers and tetramers of USP25, exist in cells

Authors

Malte Gersch, Jane L. Wagstaff, Angela V. Toms, Bradford Graves, Stefan M.V. Freund, David Komander

Correspondence

dk@wehi.edu.au

In Brief

Deubiquitinating enzymes restrict the degree of ubiquitin-mediated signaling in cells, and their activity must therefore be tightly regulated. In this manuscript, Gersch et al. show how the catalytic activity of USP25, but not of its closely related paralog USP28, is regulated by oligomerization through sequences inserted into their catalytic domains.

Gersch et al., 2019, Molecular Cell *74*, 436–451
May 2, 2019 © 2019 Medical Research Council Laboratory of Molecular Biology. Published by Elsevier Inc.
https://doi.org/10.1016/j.molcel.2019.02.030

CellPress

Distinct USP25 and USP28 Oligomerization States Regulate Deubiquitinating Activity

Malte Gersch,[1,2,3] Jane L. Wagstaff,[1] Angela V. Toms,[4] Bradford Graves,[4] Stefan M.V. Freund,[1] and David Komander[1,5,6,7,*]

[1]Medical Research Council Laboratory of Molecular Biology, Francis Crick Avenue, Cambridge CB2 0QH, UK
[2]Chemical Genomics Centre, Max-Planck-Institute of Molecular Physiology, Otto-Hahn-Str. 11, 44227 Dortmund, Germany
[3]Department of Chemistry and Chemical Biology, Technical University Dortmund, Otto-Hahn-Str. 4a, 44227 Dortmund, Germany
[4]FORMA Therapeutics, Arsenal Street, Watertown, MA 02472, USA
[5]Ubiquitin Signalling Division, The Walter and Eliza Hall Institute of Medical Research, 1G Royal Parade, Parkville, VIC 3052, Australia
[6]Department of Medical Biology, The University of Melbourne, Melbourne, VIC 3010, Australia
[7]Lead Contact
*Correspondence: dk@wehi.edu.au
https://doi.org/10.1016/j.molcel.2019.02.030

SUMMARY

The evolutionarily related deubiquitinating enzymes (DUBs) USP25 and USP28 comprise an identical overall domain architecture but are functionally non-redundant: USP28 stabilizes c-MYC and other nuclear proteins, and USP25 regulates inflammatory TRAF signaling. We here compare molecular features of USP25 and USP28. Active enzymes form distinctively shaped dimers, with a dimerizing insertion spatially separating independently active catalytic domains. In USP25, but not USP28, two dimers can form an autoinhibited tetramer, where a USP25-specific, conserved insertion sequence blocks ubiquitin binding. In full-length enzymes, a C-terminal domain with a previously unknown fold has no impact on oligomerization, but N-terminal regions affect the dimer-tetramer equilibrium *in vitro*. We confirm oligomeric states of USP25 and USP28 in cells and show that modulating oligomerization affects substrate stabilization in accordance with *in vitro* activity data. Our work highlights how regions outside of the catalytic domain enable a conceptually intriguing interplay of DUB oligomerization and activity.

INTRODUCTION

The complement of human deubiquitinases (DUBs) comprises ~100 enzymes, including more than 50 ubiquitin-specific proteases (USPs) (Clague et al., 2013; Mevissen and Komander, 2017). USPs are precision tools that bind and deubiquitinate their substrates, often leading to substrate stabilization. What is less clear is the degree of redundancy in the system, particularly for subsets of highly similar USPs that most likely arose from gene duplication events (Figure S1A). Biological roles of DUBs are currently emerging (Clague et al., 2013), and the available data suggest non-redundant roles even for highly similar enzymes.

A striking case is the DUB pair USP28 and USP25 (Figures S1B and S1C). USP28 has been identified as a regulator of the DNA damage response (DDR) (Zhang et al., 2006) and of transcription via stabilization of c-MYC (Popov et al., 2007), which has led to intense study. USP28 adopts a mostly nuclear localization, and reported substrates include LSD1 involved in chromatin and DNA methylation (Wu et al., 2013) and 53BP1 involved in DDR and cell cycle regulation (Cuella-Martin et al., 2016). Several functions of USP28 have been linked to cullin-SCF E3 ligase biology, and genetic analysis suggested a connection between USP28, FBW7, and substrate levels (Cremona et al., 2016). Pathophysiologically, USP28 drives colorectal and non-small-cell lung cancer (Diefenbacher et al., 2014; Wang et al., 2018), which has made it a sought-after pharmacological target (Harrigan et al., 2018).

In contrast, USP25 has been linked to various cytosolic roles (Figure S1C), mostly involving inflammatory processes. Viruses and cytokines trigger ubiquitin-dependent activation of nuclear factor κB (NF-κB) and cell death, and these processes are under tight control by deubiquitinases (Harhaj and Dixit, 2012). USP25 has been genetically shown to negatively regulate TRAF3 and TRAF6 signaling downstream of Toll-like receptor 4 (TLR4) (Lin et al., 2015; Zhong et al., 2013) and TRAF3 and TRAF6 signaling downstream of the interleukin-17 (IL-17) receptor (Zhong et al., 2012). USP25 also binds tankyrases, linking it to Wnt signaling (Xu et al., 2017). Overall, USP25 and USP28 are among the biologically well-studied DUBs, yet how they regulate such diverse cellular processes is largely unknown.

USP25 and USP28 share an identical domain structure and highly homologous catalytic domains (Figure S1B). An N-terminal region incorporates a number of ubiquitin-binding domains (ubiquitin-associated [UBA] and 2 ubiquitin-interaction motifs [UIMs]) as well as a SUMO2/3-selective SUMO-interaction motif (SIM) that drives SUMOylation of a neighboring Lys residue. Modification with SUMO prevents UIM-mediated ubiquitin interactions required for efficient ubiquitin chain cleavage (Meulmeester et al., 2008), which seems to be shared by USP28

 © 2019 Medical Research Council Laboratory of Molecular Biology. Published by Elsevier Inc.
This is an open access article under the CC BY license (http://creativecommons.org/licenses/by/4.0/).

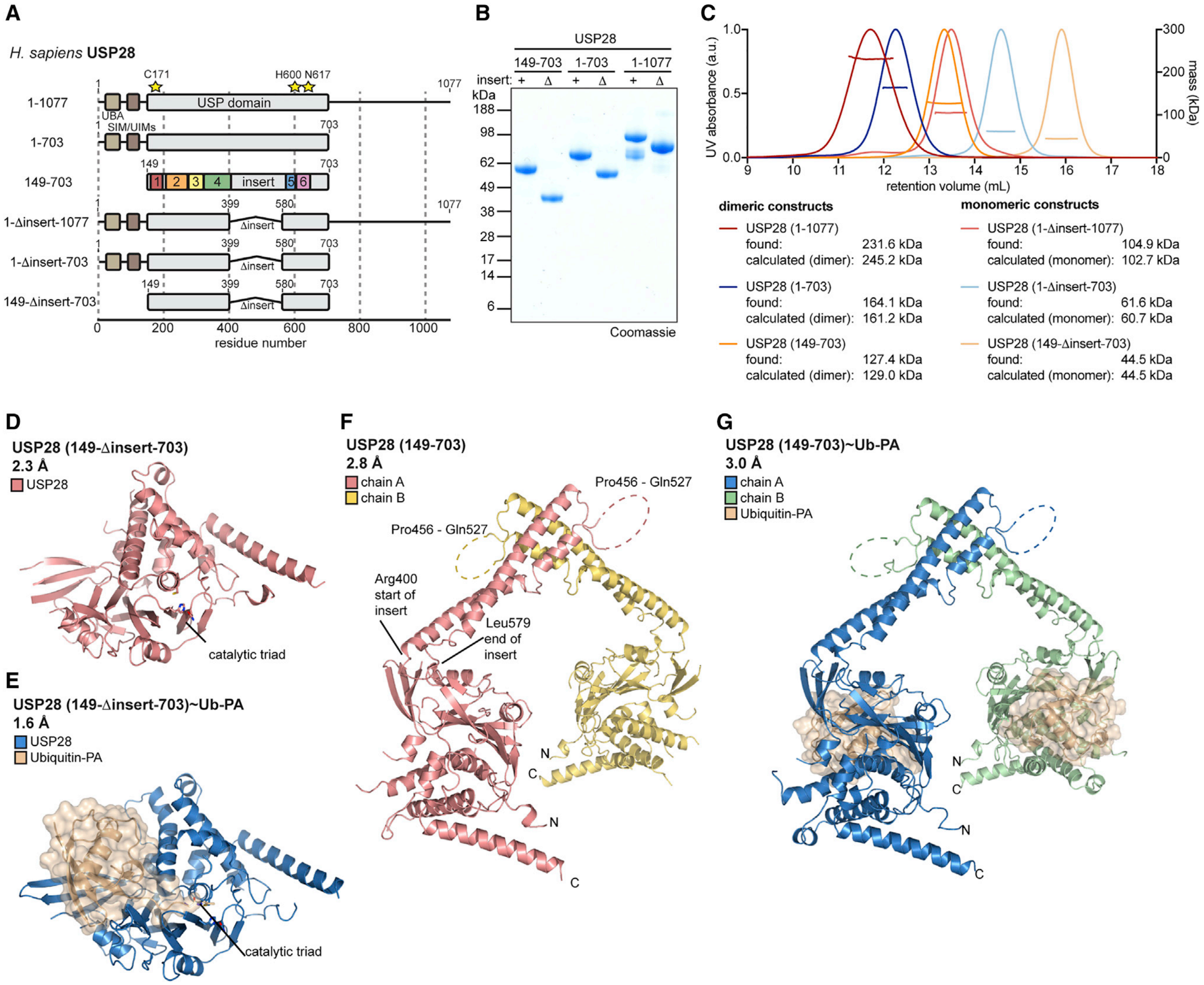

Figure 1. An Insertion in the Catalytic Domain of USP28 Mediates Its Dimerization

(A) Schematic representation of human USP28 constructs. The catalytic domain is shown in gray; an N-terminal UBA domain, SUMO-interaction motif (SIM), and ubiquitin-interaction motifs (UIMs) are shown in brown. Residues of the catalytic triad are shown as yellow stars. Colored boxes refer to USP box annotation (Ye et al., 2009).

(B) Coomassie-stained SDS-PAGE gel of indicated recombinant proteins.

(C) SEC-MALS analysis of proteins shown in (B). Identified masses are matched to either monomeric (light colors) or dimeric (dark colors) expected masses.

(D and E) Crystal structures of catalytic domains of USP28 with the insertion deleted in the apo form (D) or bound to ubiquitin-propargylamine (Ub-PA; transparent surface) (E). Catalytic triad residues are indicated.

(F and G) Crystal structures of the catalytic domain of human USP28 in its apo form (F) or bound to Ub-PA (G). N and C termini, insertion boundaries, and disordered residues are indicated.

(Zhen et al., 2014). NMR analysis showed that the N-terminal domains are embedded within a flexible sequence context (Yang et al., 2017).

Less is known about the remaining 85% of the enzymes. USP catalytic domains are usually around 350 amino acids (aa) in length; however, in USP25/28, the catalytic domains span ~550 aa due to a large, conserved insertion of unknown function at a common insertion point between USP boxes 4 and 5 (Figure 1A; Ye et al., 2009). The catalytic domains are followed by an unannotated region of ~340 aa of unknown structure; several reports suggest roles in substrate binding for the C-terminal part (Xu et al., 2017).

We here characterize USP25 and USP28 at the molecular level and report crystal structures of the catalytic domains with and without insertions, and with and without ubiquitin, as well as a structure of the USP25 C-terminal domain. We validate the structures on a biochemical level and in cells on structural and functional levels to explain the molecular distinction of USP25 and USP28. Our data reveal a further paradigm for USP regulation, showing how similar domain architectures were adopted to

Table 1. Data Collection and Refinement Statistics

	USP28 (149-Δinsert-703) (PDB: 6HEH)	USP28 (149-Δinsert-703)∼Ub-PA (PDB: 6HEI)	USP28 (149-703) (PDB: 6HEJ)	USP28 (149-703)∼Ub-PA (PDB: 6HEK)	USP25 (157-714) (PDB: 6HEL)	USP25 (748-1048) (PDB: 6HEM)
Data Collection						
Beamline	ESRF ID30B	Diamond I03	ESRF ID30B	Diamond I04-1	ESRF ID29	Diamond I02
Wavelength (Å)	0.9686	0.9762	0.9763	0.9282	0.9790	0.9795
Space group	$I4_132$	$P222_1$	$I222$	$I222$	$I4$	$P2_12_12_1$
a, *b*, *c* (Å)	189.15, 189.15, 189.15	49.51, 86.41, 97.85	104.24, 200.46, 206.06	103.21, 199.79, 204.90	139.18, 139.18, 190.47	55.96, 78.29, 84.91
α, β, γ (°)	90, 90, 90	90, 90, 90	90, 90, 90	90, 90, 90	90, 90, 90	90, 90, 90
Anisotropy correction	–	–	yes	yes	yes	–
Total reflections	233,139 (20,128)	210,197 (20,685)	163,141 (7,235)	206,967 (18,758)	111,871 (6,207)	184,886 (17,717)
Unique reflections	27,219 (2,698)	52,145 (5,114)	31,781 (1,590)	30,634 (2,785)	20,988 (1,050)	40,292 (3,983)
Resolution (Å)	50.55–2.26 (2.34–2.26)	49.51–1.64 (1.70–1.64)	143.68–2.79 (3.03–2.79)	143.05–3.03 (3.36–3.03)	59.17–2.94 (3.39–2.94)	57.56–1.72 (1.78–1.72)
Ellipsoidal resolution limits (Å) [direction]	–	–	4.48 [a*]	3.81 [a*]	3.86 [a*]	–
			2.79 [b*]	3.15 [b*]	3.86 [b*]	
			2.80 [c*]	3.02 [c*]	2.94 [c*]	
R_{merge}	0.065 (0.699)	0.049 (0.637)	0.053 (1.03)	0.100 (1.24)	0.052 (1.43)	0.071 (0.751)
R_{meas}	0.068 (0.752)	0.057 (0.733)	0.059 (1.16)	0.109 (1.35)	0.058 (1.57)	0.080 (0.852)
$I/\sigma(I)$	19.8 (2.7)	13.5 (2.3)	15.6 (1.5)	14.2 (1.6)	15.9 (1.2)	13.5 (2.5)
CC1/2	0.999 (0.827)	0.998 (0.690)	0.998 (0.551)	0.999 (0.608)	0.999 (0.501)	0.996 (0.755)
Wilson B-factor (Å^2)	44	24	115	92	103	21
Spherical completeness (%)	99.8 (99.2)	99.8 (99.5)	58.8 (13.6)	73.6 (25.3)	54.6 (7.8)	99.9 (99.9)
Ellipsoidal completeness (%)	–	–	93.5 (62.7)	95.2 (78.1)	93.4 (70.1)	–
Redundancy	8.6 (7.5)	4.0 (4.0)	5.1 (4.6)	6.8 (6.7)	5.3 (5.9)	4.6 (4.4)
Refinement						
Molecules/ASU	1	1	2	2	2	1
Reflections used for refinement	27,199 (2,681)	52,143 (5,114)	31,767	30,626	20,964	40,291 (3,982)
R_{work}/R_{free} (%)	19.6/21.5	18.6/21.4	26.1/28.6	21.8/23.7	25.4/27.8	18.1/20.9
No. atoms	2,872	3,693	6,531	8,503	7,163	2,793
Protein	2,739	3,409	6,451	8,477	7,147	2,431
Water	128	276	70	–	16	349
B-factors (Å^2)	51.5	29.8	89.1	91.9	80.8	29.3
Protein	51.5	29.1	89.2	92.0	80.8	27.9
Water	50.3	37.8	76.1	–	53.2	38.8

(*Continued on next page*)

Table 1. *Continued*

	USP28 (149-Δinsert-703) (PDB: 6HEH)	USP28 (149-Δinsert-703)~Ub-PA (PDB: 6HEI)	USP28 (149-703) (PDB: 6HEJ)	USP28 (149-703)~Ub-PA (PDB: 6HEK)	USP25 (157-714) (PDB: 6HEL)	USP25 (748-1048) (PDB: 6HEM)
RMSDs						
Bond lengths (Å)	0.004	0.007	0.006	0.005	0.018	0.008
Bond angles (°)	0.92	1.21	1.05	1.00	1.33	1.16
Ramachandran statistics: favored/allowed/outliers (%)	96.6/3.4/0.0	97.8/2.2/0.0	96.7/3.3/0.0	96.4/3.5/0.1	95.8/4.0/0.2	99.3/0.7/0.0
Rotamer outliers (%)	0.0	0.0	12.9	3.3	8.1	0.0
Clashscore	3.8	3.3	13.4	9.3	19.7	1.7

Statistics for the highest-resolution shell are shown in parentheses. Merging statistics for anisotropy-corrected datasets were calculated from ellipsoidally truncated data. Asterisks indicate reciprocal cell directions.

exist in cells in active and autoinhibited conformations, in this case by virtue of oligomerization via USP-specific insertions.

RESULTS

Biochemical Characterization of USP28

Human USP28 comprises 1,077 aa, of which the N-terminal 150 have been structurally characterized (Zhen et al., 2014). To gain insights into the catalytic domain architecture, we designed constructs with boundaries according to previous studies and secondary structure predictions (Zhen et al., 2014). Box annotation was used to identify and remove a 180-aa insertion within the catalytic domain to aid structural biology (Gersch et al., 2017; Ye et al., 2009; Figure 1A). Proteins were expressed and purified from *E. coli*, yielding homogeneous material for most constructs (Figure 1B). Unusual running behavior on gel filtration prompted more detailed size-exclusion chromatography multi-angle light scattering (SEC-MALS) analysis, revealing that proteins lacking the insertion were monomeric, and USP28 variants including the insertion were dimeric (Figure 1C). This was unchanged when catalytic domains were modified with the ubiquitin-based reactive probe ubiquitin-propargylamine (Ub-PA) (Ekkebus et al., 2013; Figure S1D). Intriguingly, at the concentrations tested, there were no apparent monomer-dimer equilibria in solution, suggesting that the insertion is necessary and sufficient to establish a tight dimerization (Figures 1C and S1D). This was further confirmed by SEC-MALS experiments using fluorescently labeled USP28 catalytic domain with Ub-PA bound: exclusively dimeric behavior was observed at concentrations below 1 nM (Figure S1E).

We further characterized DUB activity by assessing cleavage of a fluorescent Ub-KG-TAMRA substrate by USP28 variants (Figures S1F and S1G). All constructs including the insertion displayed near-identical catalytic efficiency, suggesting that N- and C-terminal regions do not interfere with a minimal ubiquitin cleavage reaction. Interestingly, deletion of the insertion decreased catalytic efficiency to ~50%, similarly for all constructs (Figure S1G).

Structural Analysis of USP28

To explain the oligomeric behavior and activity differences, we initiated structural studies. A variety of crystal structures of USP28 were determined for constructs spanning the catalytic domain (aa 149–703; Figures 1D–1G and S1H–S1K; Table 1). Crystals of USP28 without the insertion diffracted to 2.3-Å resolution (Figure 1D). Crystals of the same protein construct modified with Ub-PA diffracted to 1.6 Å (Figure 1E), and the structures were determined by molecular replacement using the previously published structure of USP7 (Hu et al., 2002) as a search model. Both structures revealed a canonical catalytic domain with high similarity to previous USP structures with and without ubiquitin; DALI analysis showed the highest similarity to USP7 (PDB: 1NBF; root-mean-square deviation [RMSD] ~2.0 Å; Z ~31.5; Figure S1L). Unlike USP7, apo USP28 showed an aligned catalytic triad (Hu et al., 2002).

Crystal structures of USP28, in which the insertion was included in the construct, yielded a dimeric entity in the asymmetric unit. An apo structure at 2.8 Å revealed a dimer of two

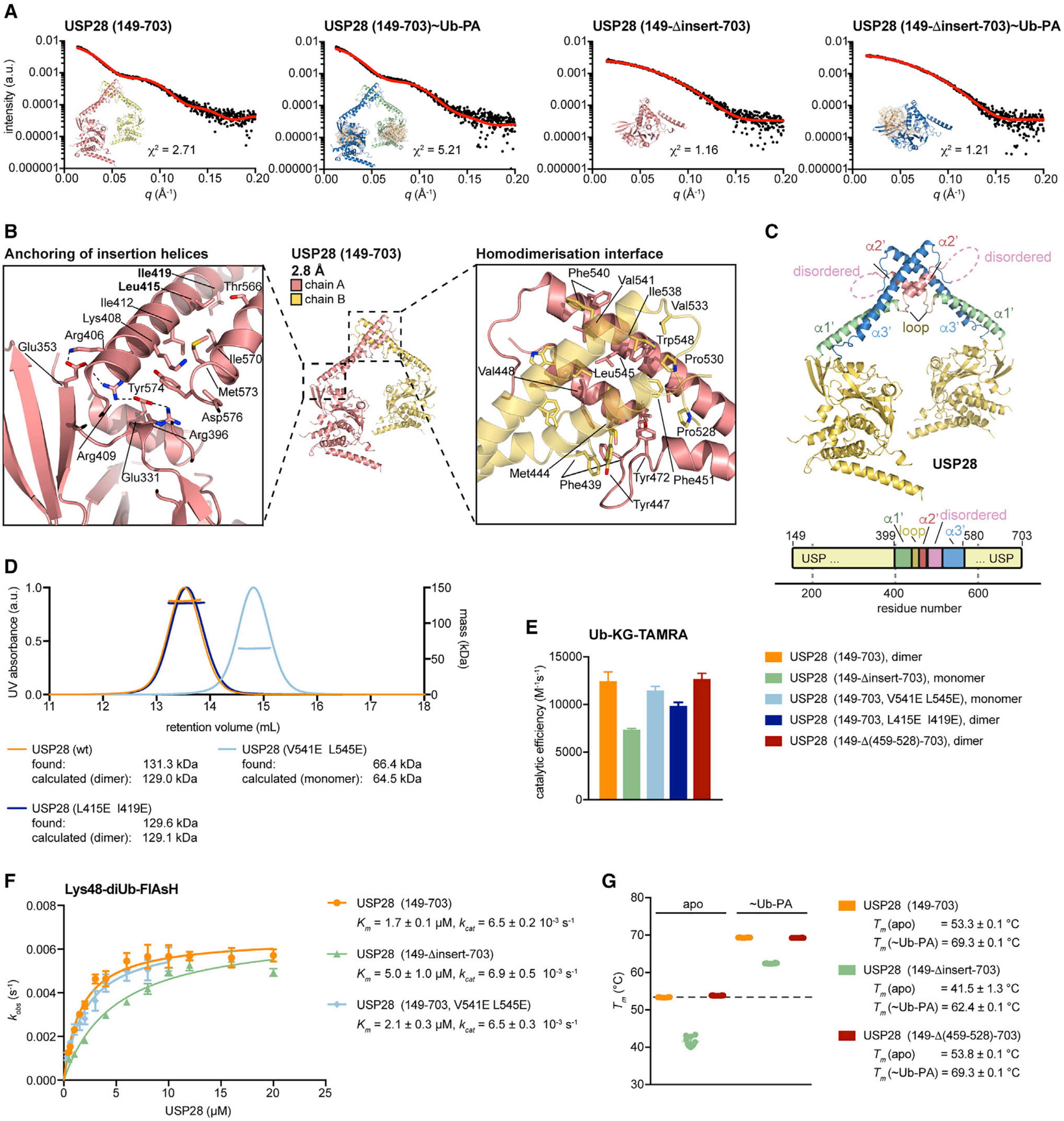

Figure 2. Structural Analysis of the USP28 Homodimerization Interface

(A) Small-angle X-ray scattering (SAXS) data (black) collected from indicated protein samples. Expected scattering curves (red) were calculated from shown crystal structures.

(B) Cartoon representation of USP28 (149–703). Boxed regions are magnified and highlight the connection of the insertion helices with the catalytic domain (left) and the dimerization interface (right).

(C) Cartoon representation (top) and schematic representation (bottom) of the USP28 (149–703) structure with the insertion annotated.

(D) SEC-MALS analysis of indicated proteins. Identified masses are matched to either monomeric or dimeric expected masses.

(E) Catalytic activities of USP28 constructs determined from Ub-KG-TAMRA cleavage assays by fluorescence anisotropy measurements. Data are shown as mean ± SD from 2–5 independent experiments. See Figure S2F for anisotropy time courses.

(legend continued on next page)

USP28 catalytic domains (Figure 1F) that are spatially separated by 56 Å (distance between catalytic Cys171 residues) through helices from the domain insertion. Consistent with spatial separation, both catalytic domains are active, as confirmed by a second structure at 3.0 Å, in which each domain is modified by Ub-PA (Figure 1G). Despite the addition of Ub-PA, both structures crystallized in near-identical crystallographic settings, perhaps explaining similar relative orientations of individual catalytic domains and dimerization domains (Figure S1M). A kink in the extended helical stalk in one molecule of the dimer generates a slight asymmetry (Figure S1N). Catalytic domains are very similar between all four structures (RMSD < 1.2 Å).

It was unclear to what degree the distinctive dimeric structure was generated by crystal packing. Small-angle X-ray scattering (SAXS) is a suitable technique to experimentally determine the shape of molecules in solution and compare it to calculated data based on a structural model (Rambo and Tainer, 2013). Indeed, although insertion-lacking catalytic domains scattered as expected from a single globular domain, a distinct SAXS profile was observed from dimeric proteins that included the insertion (Figure 2A). Calculated scattering curves from the crystal structure models were an excellent fit for all SAXS measurements in solution (Figure 2A). SAXS data can also be expressed as pairwise distance distribution functions, which for monomeric USP28 samples revealed a single peak ($r \sim 35$ Å), corresponding to internal distances within an isolated catalytic domain. Characteristically, dimeric USP28 proteins showed a second peak ($r \sim 80$ Å) that closely reflected the average distance between the centers of mass of two catalytic domains in the crystal structures (Figure S2A; Table S1). Overall, SAXS data collected in solution were in excellent agreement with the dimeric USP28 crystal structure.

This suggested that the dimerized insertion domain is relatively rigidly anchored to the catalytic core. Molecularly, outgoing and incoming helices that form the stalk of the dimerization domain appeared indeed anchored to the catalytic domain by polar contacts (Figure 2B). The insertion of USP28 protrudes as an anti-parallel α-helical stalk from the base of the fingers subdomain. A 40-aa helix ($\alpha1'$) at the start breaks to form a second, shorter (15-aa) helix ($\alpha2'$), and both pack against an anti-parallel 60-aa helix ($\alpha3'$) with hydrophobic interactions (Figures 2C and S2B–S2D). The $\alpha2'$ and $\alpha3'$ helices form a pseudo-symmetric, hydrophobic interface with the second molecule in the dimer (Figure 2B), with further contacts via the short loop between $\alpha1'$ and $\alpha2'$. Together, this generates a $\sim 100°$ angle between the helical stalks (Figure 2C). Intra- and intermolecular interface residues within the insertions are invariant in evolution (Figures S2B and S2C). A linker between the dimerization-helices $\alpha2'$ and $\alpha3'$ comprises 72 residues (aa 456–527) that are not conserved and disordered in the structures. Deletion of this region does not impact on dimerization behavior and has no apparent effect on activity *in vitro* (Figures 2E, S2E, and S2F).

Double-point mutations to disrupt dimerization (V541E L545E) or to destabilize the stalk of the insertion (L415E I419E) were introduced and showed expected monomeric or dimeric behavior on gel filtration (Figure 2D). Importantly, although removal of the insertion affected activity toward an Ub-KG-TAMRA substrate (Figures 2E and S1G), monomerized USP28 (V541E L545E) showed identical activity compared to dimeric USP28, and a variant with the insertion-destabilizing mutation (L415E I419E) was only mildly impaired in activity (Figure 2E).

We investigated the activity differences further by a full kinetic characterization using a Lys48-linked diUb-FlAsH substrate (Pruneda et al., 2016; Figure 2F). Wild-type and monomeric (V541E L545E) USP28 displayed virtually identical kinetic parameters, whereas deletion of the insertion led to a 3-fold reduction in K_m but no change in k_{cat}. Thermal shift analysis revealed that deletion of the insertion led to a drastic decrease in protein stability in both the apo and Ub-PA-bound forms, whereas deletion of the disordered region in the center of the insertion did not affect protein stability (Figure 2G).

These data showed that the USP28 insertion per se has no strong impact on activity but indirectly contributes to stabilization of the catalytic domain and its ubiquitin-binding site. Notably, the insertion protrudes out of the catalytic domain just behind the distal ubiquitin binding site (Figure S1L). A role of the insertion in stabilizing the ubiquitin-binding site is further supported by the observation that parts of the fingers subdomain in the insert-deleted apo structure were disordered but ordered in Ub-PA-bound structures (Figures 1D and 1E). We conclude that USP28 is a dimer with separated, independently active catalytic domains (Figure S2G).

Distinct Biophysical Behavior of USP25

Next, we focused our attention on USP25, whose sequence has an overall identity and similarity with USP28 of 48% and 76%, respectively, and which includes a similar insertion. Corresponding catalytic domain constructs of USP25 with and without insertion were purified (Figures 3A and 3B) and compared by SAXS and SEC-MALS in apo and Ub-PA-bound states.

SAXS profiles of insertion-deleted constructs and of the insertion-containing covalent USP25~Ub-PA complex were perfectly matched by calculated profiles from respective USP28 models (Figures 3C and S3A). Strikingly, however, the apo version of insertion-containing USP25 catalytic domain showed a distinctive SAXS profile that did not fit the profile derived from the USP28 apo structure (Figure 3C).

This was also reflected by SEC-MALS analysis. Monomeric behavior of insertion-lacking USP25 variants and dimeric behavior of the USP25~Ub-PA complex contrasted a tetrameric behavior observed for the USP25 catalytic domain when the insertion was present (Figure 3D). SAXS distance distribution analysis revealed a broad, single-peak distribution, indicative of a large, globular shape (Figure S3B).

(F) Catalytic activities of USP28 constructs determined from Lys48-diUb-FlAsH cleavage assays. Data are shown as mean ± SE from 3 independent experiments performed in technical triplicates. Kinetic parameters obtained from fitted curves are listed.

(G) Protein melting temperatures from thermal shift assays of indicated USP28 protein samples, either in the apo or Ub-PA-bound forms. Individual data points are plotted ($n = 10$), and melting temperatures are listed as mean ± SD.

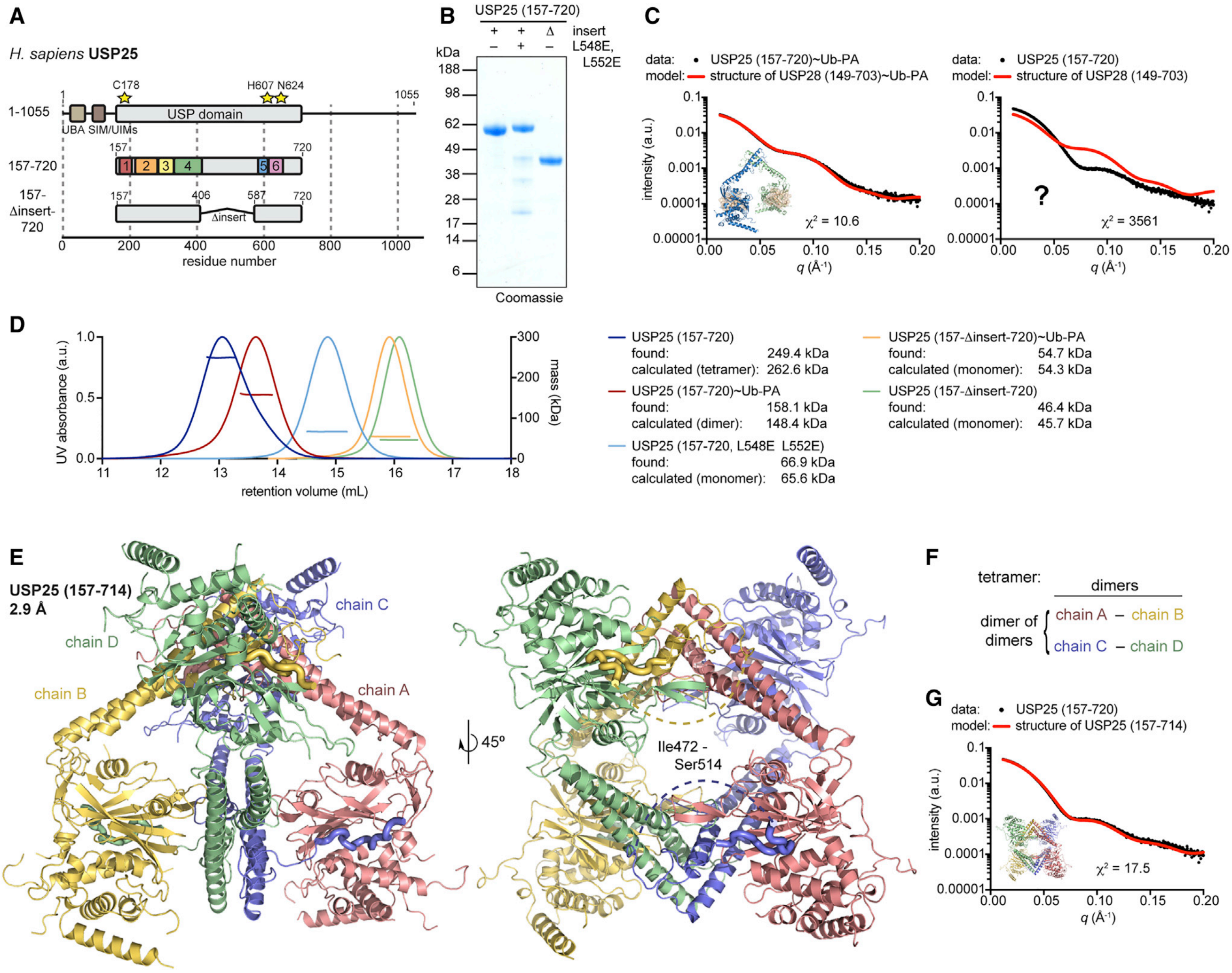

Figure 3. The Catalytic Domain of USP25 Is Tetrameric

(A) Schematic representation of human USP25 constructs as in Figure 1A. A construct ending at residue 714 was used for crystallization.

(B) Coomassie-stained SDS-PAGE gel of indicated recombinant proteins.

(C) SAXS data (black) collected from indicated USP25 protein samples and overlaid with expected scattering curves (red) calculated from USP28 crystal structures, indicating the distinct scattering of apo USP25.

(D) SEC-MALS analysis of indicated USP25 proteins. Identified masses are matched to either monomeric (light colors) or multimeric (dark colors) expected masses.

(E) Crystal structure of the catalytic domain of USP25 in two orientations. Chains A and B constitute the asymmetric unit, and chains C and D are shown from symmetry-related molecules. Residues 515–528, which contact the catalytic domain of adjacent chains, are shown as thick tubes. Dotted lines indicate disordered residues.

(F) Schematic of the USP25 tetramer as dimer of dimers.

(G) SAXS data (black, repeated from C for clarity) overlaid with the expected scattering curve (red) calculated from the tetrameric USP25 crystal structure.

Structure of Tetrameric USP25 Catalytic Domain

Attempts to crystallize this intriguing USP25 tetramer eventually resulted in a 2.9-Å crystal structure (Figures 3E, S3C, and S3D; Table 1). Interestingly, USP25 formed a dimer of dimers (Figure 3F), wherein the distinctive, V-shaped dimerization interface of one dimer inserted between catalytic domains of a second dimer, forming a symmetric tetramer (Figure 3E). Calculation of SAXS profiles from this structure generated a perfect fit for the previously unassigned experimental data (Figure 3G). Also, as expected from the ubiquitin-bound USP25 dimer observed in SAXS, the individual dimers of apo USP25 resembled USP28, with structurally similarly dimerized insertions and catalytic domains (Figures S3E–S3H). Superposition based on the dimerization domain reveals a slight rotation of catalytic domains with respect to the insertion; superposition based on the catalytic domain explains how this is generated by a different angle of how the insertions protrude from the catalytic core (Figure S3H). As in USP28, all residues that interact in *cis*

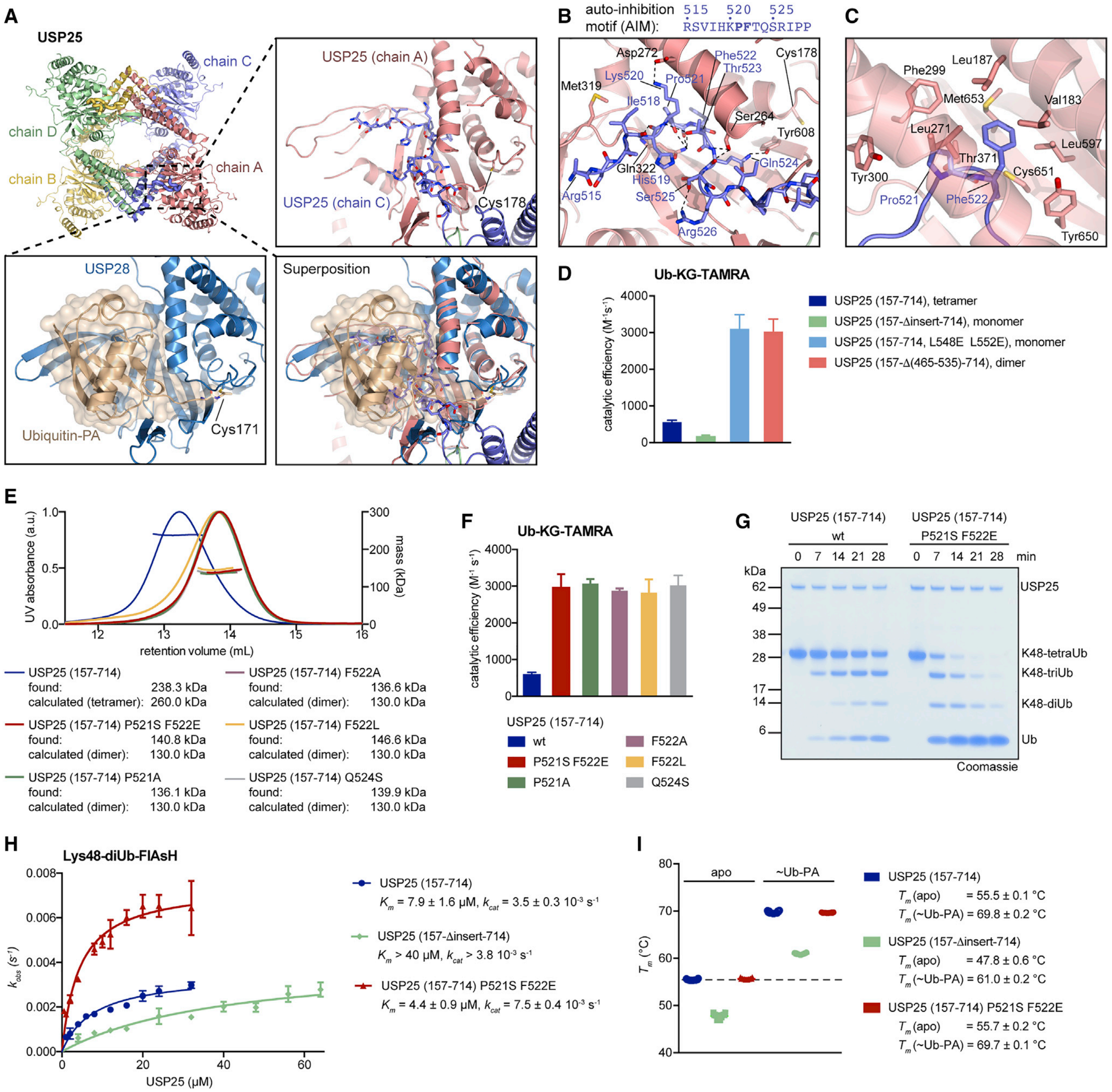

Figure 4. Tetramerization of USP25 Mediates Autoinhibition In *trans* through a Conserved AIM within the Domain Insertion

(A) Top left: cartoon representation of the tetrameric catalytic domain of USP25. The area shown as close up is indicated. Top right: close-up view of chain A with the AIM of chain C shown as sticks is shown. Bottom left: USP28 (149-Δinsert-703) bound to Ub-PA under a semitransparent surface is shown. Bottom right: Superposition of the close ups shows the mutually exclusive binding of the AIM and ubiquitin. The catalytic cysteines Cys171 (USP25) and Cys178 (USP25) are shown for orientation.

(B) Detailed view of the interaction between AIM and ubiquitin-binding site of USP25. Dashed lines indicate polar interactions.

(C) A hydrophobic pocket in the USP25 catalytic domain accommodates Pro521 and Phe522 of the AIM.

(D) Catalytic activities of USP25 constructs determined from Ub-KG-TAMRA cleavage assays by fluorescence anisotropy measurements. Data are shown as mean ± SE from 5 independent experiments. See Figure S4G for anisotropy time courses.

(E) SEC-MALS analysis of indicated USP25 proteins. Identified masses are matched to either tetrameric or dimeric expected masses.

(F) Catalytic activities of USP25 constructs analyzed in (E), determined from Ub-KG-TAMRA cleavage assays by fluorescence anisotropy measurements. Data are shown as mean ± SE from 3 independent experiments. See Figure S4H for anisotropy time courses.

(legend continued on next page)

and in *trans* are highly conserved throughout species (Figures S3E–S3G), and conserved residues coincide with residues that were observed to be structured (Figure S3F). Moreover, mutation of the equivalent set of hydrophobic residues in the dimerization interface (L548E L552E) led to the formation of monomeric USP25 (Figure 3D), further substantiating the notion that a dimeric arrangement is required for tetramerization.

An Auto-inhibition Motif Links USP25 Oligomerization to Activity

Tetramerization orients catalytic domains such that ubiquitin-binding sites face outward and are accessible to solvent. Blocking loops 1 and 2, regulatory elements that line the C terminus of a bound ubiquitin (Clerici et al., 2014), are pried open by direct interactions with the insertion stalk provided by the second dimer.

Importantly, we found that the tetramer was autoinhibited. A 14-residue section of the linker that is disordered in USP28 becomes ordered in USP25 and binds the ubiquitin binding site in a neighboring catalytic domain in the tetramer; this way all four catalytic domains are unable to bind ubiquitin (Figures 3E, 4A, and S4A–S4C). The ordered 14-mer autoinhibitory motif (AIM) is highly conserved in USP25 and centers on a Pro-Phe motif that inserts deeply into a grove below the α5 helix of the catalytic domain (Figures 4B, 4C, S4D, and S4E).

We confirmed the autoinhibitory nature of the AIM biochemically. Removal of the AIM in a construct that lacked all residues disordered in USP28 (construct 157-Δ(465-535)-714) led to dimeric USP25 (Figure S4F), which was 6-fold more active than tetrameric wild-type protein (Figures 4D and S4G). The same activity was obtained for a USP25 construct that included the AIM but was monomerized by mutations in the dimerization domain (L548E L552E). Deletion of the entire insertion (i.e., including the dimerization region) decreased activity, similar to what had been seen for USP28 (Figures 4D and S1G). Point mutations within the Pro-Phe motif or of the adjacent Gln524 disrupted the tetramer (Figure 4E) and activated USP25 accordingly for Ub-KG-TAMRA (Figures 4F and S4H) as well as polyubiquitin (Figure 4G) cleavage. Quantitative kinetic analysis of Lys48-diubiquitin-FlAsH cleavage revealed that the activation mediated by the P521S F522E mutation was due to the combination of a 2-fold higher k_{cat} and a 2-fold lower K_m (Figure 4H). As for USP28, deletion of the insertion of USP25 led to a more than 5-fold reduction in K_m (Figure 4H) and a large protein destabilization (Figure 4I). Tetrameric and dimeric forms of USP25 showed identical melting temperatures. These data confirm that the insertions of both USP25 and USP28 are important to stabilizing the catalytic domains and their ubiquitin-binding sites.

Mechanism of the AIM

The AIM spans across the ubiquitin-binding site at the connection between the Palm and Fingers subdomains. The entire stretch would interfere with ubiquitin binding, as the AIM physically occupies much of the ubiquitin binding surface, forming numerous polar contacts (Figure 4B). Importantly, strong anchoring is provided by a section of the AIM, aa 519–524, that inserts itself into the core of the catalytic domain, occupying an unexpected pocket below the α5 helix. In particular, Pro521 and Phe522 rest deeply within the USP core (Figures 4C and S4A), and this interaction acts as a wedge that pushes the α5 helix away from the catalytic domain, e.g., when compared to apo and Ub-PA-bound states of USP28 (Figures S4D and S4E). A clear difference between inhibited and active structures is the conformation of an α5 hydrophobic core residue, Phe259 (USP25)/Phe266 (USP28), the sidechain of which rotates and moves >7 Å within the Palm subdomain; in active USP28, it anchors α5 in an active position and links the α5 helix to the activity state of the catalytic cysteine (Figure S4E).

The importance of correct α5 helix positioning as a regulatory mechanism has previously been noted also for USP7. A C-terminal activating peptide of USP7 binds on the opposite site of α5 compared to the AIM and seemingly pushes it into an active position (Figure S4D; Faesen et al., 2011; Rougé et al., 2016), and USP7 point mutations to improve α5 anchoring activate the enzyme (Özen et al., 2018). Together, this emphasizes the important role of α5 positioning in activating and inhibiting USP enzymes.

Cleavage of polyubiquitin requires the binding of a distal and of a proximal ubiquitin moiety across the active site. Superposition of tetrameric USP25 and of the structure of inactive USP30 in complex with Lys6-linked diubiquitin revealed that a catalytic domain of USP25 within the tetramer is unable to bind polyubiquitin because the proximal ubiquitin would clash with the helical stalks of another dimer (Figure S4I). Consequently, the ability of tetrameric USP25 to cleave polyubiquitin indicates that autoinhibited tetrameric and dimeric forms are in an equilibrium. Because disruption of the tetramer activates the enzyme for cleavage of longer chains, polyubiquitin alone seems insufficient to fully activate USP25 (Figure 4G). This transient dissociation of the tetramer would also explain that Ub-PA was able to generate a dimeric USP25 species (Figure 3D).

We conclude that USP25 forms an autoinhibited dimer of dimers, in which the insertion not only performs the key underlying dimerization role but also acts, via its AIM, as a regulatory element. We are not aware that a similar mechanism of activity regulation for another DUB has been described (Mevissen and Komander, 2017).

A Dimer-Tetramer Equilibrium in Full-Length USP25

We next set out to understand whether regions outside of the catalytic domain can regulate the oligomeric state of USP25. The region N-terminal to the catalytic domain comprises several regulatory elements embedded into a flexible sequence context (Meulmeester et al., 2008; Zhen et al., 2014; Yang et al., 2017). A

(G) Time course analysis of Lys48-linked tetraUb cleavage. The assay was performed three times with consistent results.

(H) Catalytic activities of USP25 constructs determined from Lys48-diUb-FlAsH cleavage assays. Data are shown as mean ± SE from 3 independent experiments performed in technical triplicates. Kinetic parameters obtained from fitted curves are listed.

(I) Protein melting temperatures from thermal shift assays of indicated samples. Individual data points are plotted (n = 10), and melting temperatures are listed as mean ± SD.

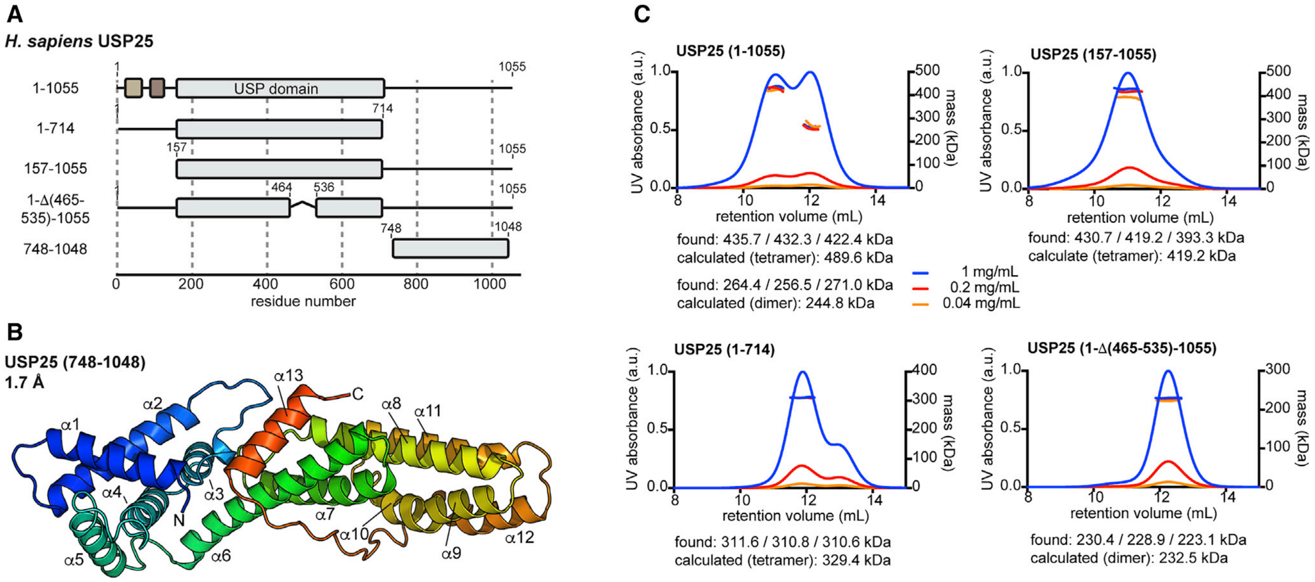

Figure 5. A Dimer-Tetramer Equilibrium in Full-Length USP25

(A) Schematic representation of human USP25 constructs as in Figure 3A. The deleted sequence between residues 464 and 536 corresponds to the sequence that was found to be disordered in USP28 and includes the AIM in USP25.

(B) Crystal structure of the C-terminal domain of USP25 in spectral colors from blue (N terminus) to red (C terminus). α helices are numbered consecutively.

(C) SEC-MALS analysis of a five-fold dilution series (blue: 1 mg/mL; red: 0.2 mg/mL; yellow: 0.04 mg/mL sample concentration) of indicated USP25 proteins. Identified masses are matched to either tetrameric or dimeric expected masses.

C-terminal region comprising ~340 aa of unknown structure and function, yet with high conservation, is shared by USP25 and USP28 (Figures 5A, S5A, and S5B). We embarked on structural studies for this domain, excluding a C-terminal peptide in USP25 (aa 1,049–1,055) that facilitates an interaction with tankyrases to mediate their stabilization (Xu et al., 2017). A 1.7-Å crystal structure of USP25 (748–1,048) was solved by *ab initio* molecular replacement (Bibby et al., 2012) and revealed an α-helical domain (Figures 5B, S5C, and S5D; Table 1) without any similarity to previously reported structures in the protein data bank according to DALI (Holm and Laakso, 2016). The C-terminal region has been implicated in substrate binding for both USP25 and USP28 (Xu et al., 2017; Bosch-Comas et al., 2006; Serra et al., 2014) and harbors the insertion site for isoform-specific sequences.

High sequence similarity suggested a similar fold in USP28 (Figure S5E), which could not be crystallized from several constructs. Despite the relatively large size, NMR spectra for the 39-kDa ^{15}N-labeled USP28 C-terminal domain (aa 736–1,077) were of high quality with discrete resonances, and relaxation behavior was consistent with a monomeric domain. NMR is an excellent method to pick up low-affinity interactions, yet the addition neither of unlabeled USP28 catalytic domain (aa 149–740) nor of unlabeled USP28 N terminus (aa 1–159) showed perturbation in the spectra, suggesting that the C-terminal domain forms an independent entity (Figure S5F). This was consistent with data that N- or C-terminal deletions of USP28 did not change its oligomerization or activity (Figures 1C and S1G).

In USP25, the situation was again different. C-terminally extended USP25 (aa 157–1,055) was exclusively tetrameric (Figures 5A and 5C), indicating that the C-terminal domain does not affect oligomeric behavior per se, as also found for USP28. In contrast, inclusion of the N terminus (aa 1–714 or full-length) unveiled a tetrameric species but also a dimeric species not present in constructs lacking the N terminus (Figures 5A and 5C). Although these data show that also full-length USP25 can adopt a tetrameric species from the dimerization of dimers, it hinted that the N terminus may regulate the oligomerization of USP25. A dimer-tetramer equilibrium as observed for full-length USP25 is consistent with the basal cleavage activity of tetrameric USP25 for polyubiquitin chains (Figures 4G and S4I).

Validation of Oligomeric States in Cells

We next sought to establish whether the distinct oligomeric states of USP28 and USP25 also form in cells. We transfected HEK293 cells with vectors encoding full-length, GFP-tagged versions of both proteins in wild-type and mutant forms and assessed their oligomerization in cell lysates by native PAGE and in-gel GFP fluorescence. We observed three oligomerization states that separated according to our predictions from the *in vitro* studies, and we assigned these as tetramer, dimer, and monomer (Figure 6A). Prompt processing of the samples after cell lysis by native PAGE was essential to observe the species assigned as tetramer (incubation of the lysate on ice for 5 h led to a decrease in its abundance, presumably due to its dissociation).

Because the migration behavior of protein complexes in native PAGE is dependent on their size as well as on their shape, we used recombinant full-length USP25 as a custom marker.

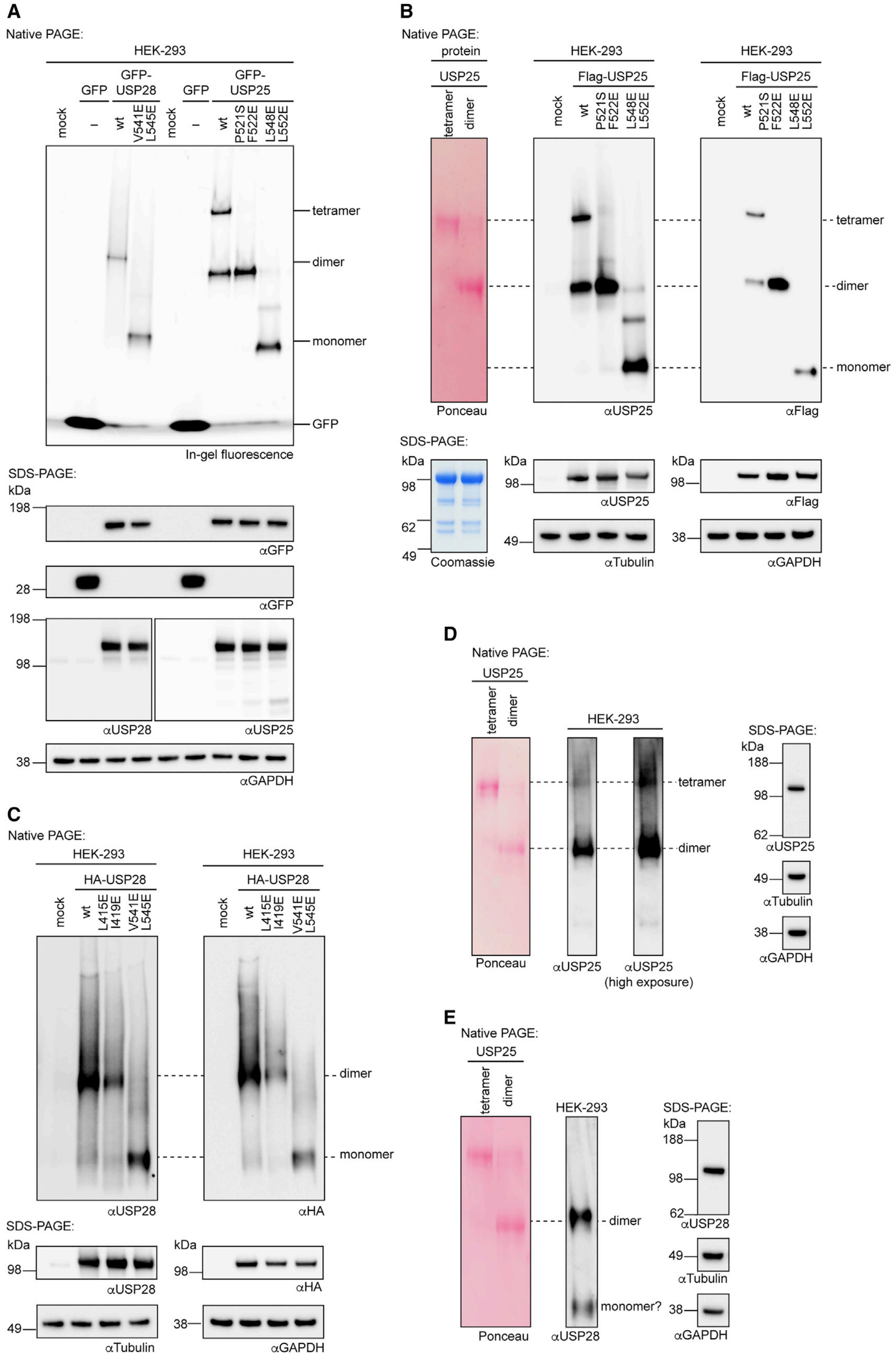

(legend on next page)

Size-exclusion chromatography immediately before native PAGE analysis allowed the separation of almost exclusively tetrameric and dimeric samples (Figures 6B and S6A). When analyzed on a native PAGE gel alongside lysates from cells that were transfected with FLAG-tagged USP25 versions, immunoblotting allowed us to validate the identity of the species directly (Figure 6B). Ectopically expressed wild-type USP25 showed both tetrameric and dimeric populations with migration behavior identical to the recombinant protein samples, whereas USP25 mutated in the AIM (P521S F522E) was exclusively dimeric. Disruption of the dimerization interface (L548E L552E) led to monomeric protein in line with the *in vitro* findings. An equivalent experiment with hemagglutinin (HA)-tagged USP28 revealed that mutation of the dimerization interface (V541E L545E), but not mutations destabilizing the stalk (L415E I419E), changed the oligomerization in cells according to *in vitro* predictions (Figures 2D and 6C).

By immunoblotting for endogenous USP25 in HEK293 lysate following native PAGE, we observed an intense band corresponding to a dimeric oligomerization and a weak band that migrated exactly as the recombinant tetrameric species (Figure 6D). Albeit weaker, this band was consistently observed in three independent experiments, supporting that endogenous USP25 exists as a tetramer in cells, albeit at low levels under steady-state conditions. No tetrameric arrangement was observed in the analysis of endogenous USP28, which was mainly dimeric, with a second, much weaker band migrating at a size consistent with a monomer (Figures 6C and 6E).

Collectively, these results demonstrate that the distinctly different oligomerization states of USP25 and USP28 also exist in cell lysate, both in ectopically expressed and endogenous proteins. This establishes the relevance of the observed structural arrangements and corroborates the role of the AIM of USP25 in mediating the formation of a tetrameric arrangement in cells. Although we cannot exclude that other proteins partake in cellular USP25 or USP28 complexes, the similar sizes of purified and cellular endogenous proteins probably suggest that USP25 and USP28 are not exclusively stably associated with large-protein machineries.

Functional Relevance of Oligomeric States

To investigate the functional consequences of disrupting the respective higher oligomeric states of USP25 and USP28, we assessed their ability to stabilize previously identified substrates in cells (Wu et al., 2013; Xu et al., 2017). When the chromatin modulator and histone demethylase LSD1 was co-expressed with USP28, both dimeric wild-type and monomeric mutant USP28 were able to stabilize LSD1 to the same degree in a concentration-dependent manner (Figure S6B). Thus, the dimeric arrangement of USP28 may have a functional relevance in other cellular contexts, but it seems dispensable for the stabilization of LSD1, in line with *in vitro* activity data (Figures 2E and 2F).

Further, we observed that strictly dimeric, mutant USP25 (P521S F522E) was more effective in stabilizing ectopically expressed tankyrase-2 than wild-type USP25 (Figure S6C). The small but consistent increase in substrate levels (Figure S6D) is in line with about one-third of the wild-type proteins forming an autoinhibited tetramer in cells under the conditions tested (see Figure 6B). These results suggest a functional role of the AIM in mediating autoinhibition by the coupling of autoinhibition and oligomerization in a cellular context.

Molecular Distinction of USP25 and USP28

The AIM in USP25 is strictly conserved (Figure 7A), whereas the equivalent sequence in USP28, which was disordered in the structures (Figures 1F and 1G), lacks conservation (Figure 7A). This suggests that autoinhibition by tetramerization has been a feature specific to USP25, but not USP28, throughout evolution (Figure 7B). To experimentally investigate this hypothesis, we assessed the oligomeric states of selected orthologs. USP25 catalytic domains from chicken (*Gallus gallus*) and mouse (*Mus musculus*) were tetrameric but became dimeric upon Ub-PA binding (Figure S7A). Like full-length human USP25 (Figure S3J), also full-length mouse USP25 showed a dimer-tetramer equilibrium (Figure S7B). In contrast, human USP28 did not display any sign of tetrameric behavior *in vitro* and in cells (Figures 1, 2, and 6). Importantly, USP28 catalytic domains from chicken and zebrafish (*Danio rerio*), and full-length zebrafish USP28, were exclusively dimeric (Figures S7A and S7B).

To establish the molecular requirements for tetramer formation of USP25, we created chimeric catalytic domains of USP25 and USP28 in which either the entire inserts (chimeras 1 and 3) or the AIM of USP25 and its equivalent sequence in USP28 (chimeras 2 and 4) were swapped (Figure S7C). SEC-MALS analysis revealed that all chimeras were clean dimers (Figure S7D), which shows that the USP25 autoinhibition motif is necessary (chimera 4 is dimeric), but not sufficient (chimeras 1 and 2 are dimeric), for tetramer formation. Instead, the molecular combination of the USP25 catalytic domain and the USP25 insertion, including its AIM, was strictly required for tetramer formation. To our surprise, we noticed sequences in several USP28

Figure 6. Analysis of Oligomerization States Adopted by USP25 and USP28 in Cells

(A) Lysates from HEK293 cells transfected with vectors for the expression of GFP-tagged proteins or controls as indicated were analyzed by in-gel fluorescence after separation by native PAGE (top) or immunoblotting after separation by SDS-PAGE (bottom).

(B) Lysates from HEK293 cells transfected with vectors for the expression of FLAG-tagged USP25 proteins or empty vector control as indicated were analyzed by immunoblotting after separation by native PAGE (top) or SDS-PAGE (bottom). As a size standard, tetrameric and dimeric fractions of recombinant, full-length human USP25 were used (see Figure S6A), visualized by Ponceau staining after native PAGE (top, same membrane as the blots to the right; see Figure S8 for uncropped blots and an overlay of the membranes) or Coomassie staining (bottom, separate gel).

(C) Analysis as in (B) with indicated HA-tagged USP28 proteins.

(D) Lysate of untreated HEK293 cells was separated by native PAGE (left) or SDS-PAGE (right) and analyzed by immunoblotting for endogenous USP25 as indicated. Size standard as in (B) is shown.

(E) Analysis as in (D) with immunoblotting for endogenous USP28.

For all assays, at least three independent experiments were performed and consistent results were obtained. See Figure S8 for uncropped blots.

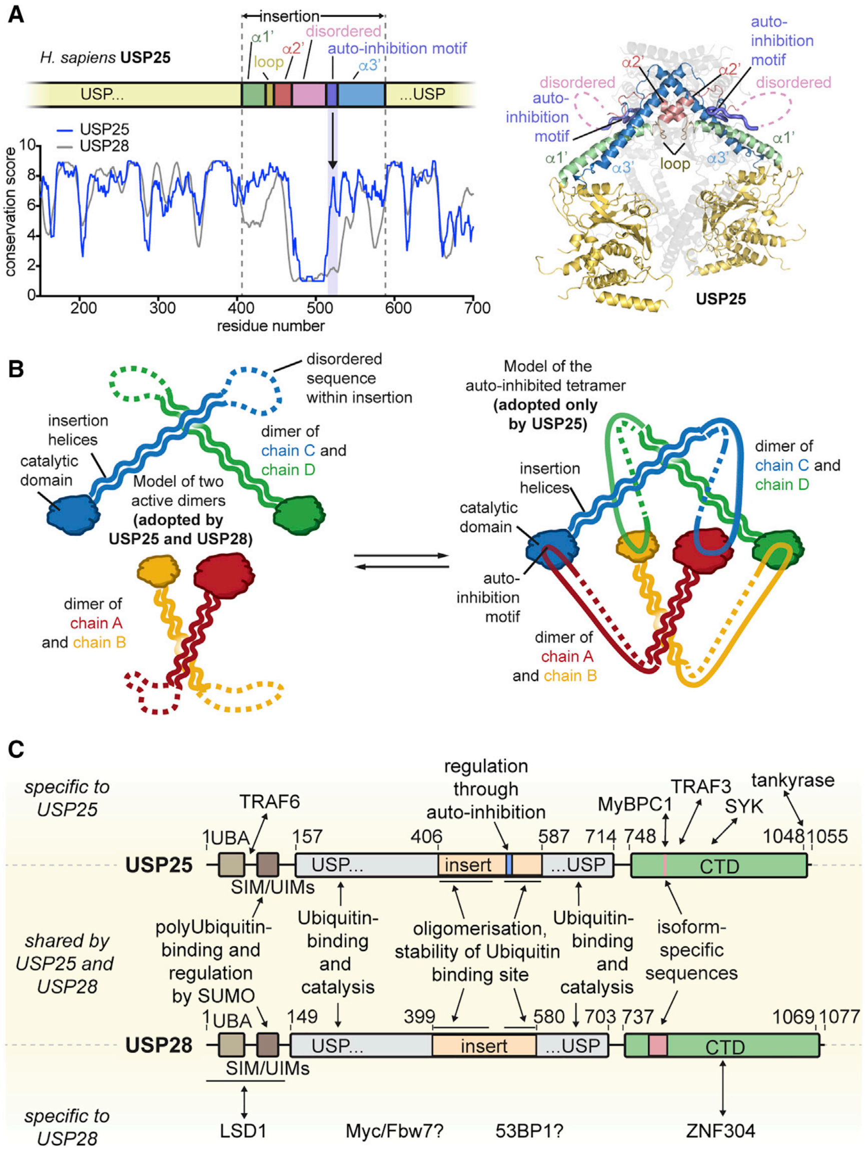

Figure 7. Molecular Comparison of USP28 and USP25

(A) Schematic representation of the sequence of the USP25 catalytic domain with parts of the insertion highlighted in different colors. Sequence conservation of USP25 and USP28 catalytic domains plotted as a 5-residue rolling window average of scores obtained from the ConSurf webserver (left). Cartoon representation of chains A and B of the USP25 crystal structure with the same color code (right). The other chains completing the tetramer are shown as transparent cartoons in gray. The spike in the USP25 sequence conservation at residue 522 corresponding to the AIM is highlighted with a black arrow.

(B) Cartoon model showing how tetramerization facilitates autoinhibition in *trans* in USP25 (left and right), but not USP28 (left).

(C) Schematic representation of domain topologies of human USP25 and USP28. Boundaries of indicated domains and motifs are shown with residue numbers. Interactions and features either specific to USP25 (top), shared by USP25 and USP28 (middle), or specific to USP28 (bottom) are linked to the relevant sequence regions with black arrows.

homologs that were near identical to the AIM of human USP25 (Figures S2C and S3F). The most prominent case was zebrafish USP28, which shares the central IHKPFTQ motif with human USP25 but has mutations in the flanking parts and is still dimeric (see chimera 5 in Figure S7D and Figure S7B). This shows that subtle changes within the AIM or around it are sufficient to prevent tetramerization.

These data help define the molecular distinction between USP25 and USP28. They show that, although the AIM is necessary, but not sufficient, for tetramerization (and autoinhibition) in USP25, the equivalent motif in USP28 is unable to induce tetramerization. Although potentially restrained by crystal packing and more flexible in solution, it is worth noting that USP25 and USP28 dimers showed large differences in the relative positions of catalytic domains with respect to insertion and AIM (Figure S3H), and this may also prevent regulatory USP28 tetramerization.

DISCUSSION

We here provide a molecular comparison of two USP enzymes, USP25 and USP28, which are highly similar at the sequence level, yet display striking differences in their oligomerization capabilities and concomitant regulation of enzymatic activities. Our data show that the catalytic domains of USP25 and USP28 share insertion sequences that (1) support distal ubiquitin binding, (2) are important for protein stability, and (3) mediate oligomerization in surprisingly distinct ways not anticipated from sequence similarity alone (Figures 7B and 7C). The presence of a conserved AIM in USP25 family members endows USP25 with a paralog-specific link of oligomerization and activity. Such coupling of oligomerization and activity is reminiscent of, e.g., UDP-α-D-glucose-6-dehydrogenase, which forms an inactive hexamer that can be broken into active dimers through allosteric regulation (Keul et al., 2018). Evolutionarily, the described mechanism likely originated from the dimer, as its shape constitutes a requirement for the formation of the observed autoinhibited tetramer (Figure 3D). All of our mechanistic insights are in full agreement with a recent crystal structure of a longer USP25 fragment in an autoinhibited tetrameric state, which forms a dimer-tetramer equilibrium in solution (Liu et al., 2018).

In addition, we validated the oligomeric differences of USP25 and USP28 in cells and observed endogenous USP25 to be mainly dimeric with a small tetrameric population (Figure 6).

This establishes the relevance of the observed oligomerizations and will fuel future work into how cells utilize these enzymatic states with different activities e.g., during signaling.

Because the tetramer of USP25 was observed only in small quantities at the endogenous level but readily formed upon overexpression, it is tempting to speculate that autoinhibition of USP25 is regulated by local concentrations. Rather than asking how autoinhibition is relieved, it may be sensible to ask how and under which conditions the tetramer is formed and the enzyme is inactivated. Molecular crowding of USP25 (by means of recruitment to, e.g., TRAF E3 ligase complexes; see Introduction) could lead to tetramer formation and quench the local deubiquitination activity, potentially enabling a negative feedback loop. Although ubiquitin, in principle, is able to overcome the autoinhibition of tetrameric USP25, the presence of polyubiquitin in the micromolar concentration range is insufficient for full activation (Figure 4D). However, we cannot exclude a regulatory role by polyubiquitin at higher local concentration. Future studies should investigate the interplay of USP25 oligomerization with ligase assemblies at their receptors.

The existence of USP28 as a constitutive dimer is also intriguing. USP28 has multiple substrates, including the important proteins FBW7, c-MYC, and 53BP1, and our structure-based refinement of the USP28 domain architecture will aid the identification of their binding mechanisms (Figure 7C). Many cullin-SCF E3 ligase complexes are dimeric, which is often facilitated by adaptor dimerization; this concept extends to FBW7 (Tang et al., 2007). Adaptation of a specific dimeric USP structure in which catalytic domains are independently active and spatially separated may invoke intriguing mechanisms of how ligase-DUB interplay is established. Despite dimerization of USP28 being dispensable for the stabilization of LSD1 (Figure S7B), future studies including putative ubiquitinated cullin-based substrates could lead to further mechanistic and biological insights into USP biology.

Collectively, we have identified molecular underpinnings of how two very similar USP enzymes can be functionally different, further highlighting that gene duplication does not necessarily facilitate functional redundancy. It is fascinating how subtle changes in sequence regulate oligomeric behavior with direct impact on enzymatic activity. All of the described features of USP25 and USP28 arise from sequences outside the core catalytic domain (Figure 7C). Our data illustrate a conceptually intriguing interplay of catalytic and non-catalytic elements in USP DUBs to achieve diverging functions of highly homologous enzymes and, for USP25, also reveal a mechanism of autoinhibition that could be pharmaceutically explored. In this respect, USP enzymes are rich in regulatory capabilities and will continue to provide a fertile ground for future discoveries, as well as provide new pharmaceutical targets.

STAR★METHODS

Detailed methods are provided in the online version of this paper and include the following:

- KEY RESOURCES TABLE
- CONTACT FOR REAGENT AND RESOURCE SHARING
- EXPERIMENTAL MODEL AND SUBJECT DETAILS
- METHOD DETAILS
  - Cloning and constructs
  - Protein expression and purification
  - Covalent ubiquitin-propargylamine complexes
  - Crystallization
  - Data collection, structure solution and refinement
  - Small-angle X-ray scattering
  - Fluorescence polarization assays
  - Gel-based ubiquitin-chain cleavage assay
  - SEC-MALS
  - Fluorescence-detection size-exclusion chromatography
  - Thermal shift assay
  - NMR spectroscopy
  - Native PAGE, in-gel GFP fluorescence and immunoblotting analysis
- QUANTIFICATION AND STATISTICAL ANALYSIS
- DATA AND SOFTWARE AVAILABILITY

SUPPLEMENTAL INFORMATION

Supplemental Information can be found online at https://doi.org/10.1016/j.molcel.2019.02.030.

ACKNOWLEDGMENTS

We thank beamline scientists at Diamond Light Source (DLS) for support at beamlines I02, I03, I04-1, and B21 and at the European Synchrotron Radiation Facility (ESRF) for support at beamlines ID29 and ID30B. Access to DLS was supported in part by the EU FP7 infrastructure grant BIOSTRUCT-X (contract no. 283570). We are grateful to Farid El Oualid (UbiQ) and Paul Geurink and Huib Ovaa (LUMC) for reagents; to Sylvie Urbé and Michael Clague (Liverpool) for discussions and reagents; to Robert Rambo and Nathan Cowieson (DLS) for support during SAXS data collections; to Ingo Greger (MRC LMB) and Janet Deane (CIMR) for access to F-SEC and SEC-MALS chromatography systems; to Andrew Leslie and Alexey Murzin (MRC LMB) for discussions; to members of the Komander lab, in particular Jonathan Pruneda, Paul Elliott, and Rune Busk Damgaard; to the DUB Alliance; and to MRC LMB for reagents, discussions, and advice. This work was supported by the Medical Research Council (U105192732), the European Research Council (724804), and the Lister Institute for Preventive Medicine. M.G. was supported through a College Post-Doctoral Associateship by Jesus College, Cambridge.

AUTHOR CONTRIBUTIONS

M.G. designed and performed all experiments. J.L.W. and S.M.V.F. performed and analyzed NMR experiments. A.V.T. and B.G. contributed initial structural and biochemical insights in the early phase of the project. M.G. and D.K. analyzed data and wrote the manuscript. D.K. directed the research and obtained funding.

DECLARATION OF INTERESTS

D.K. is part of the DUB Alliance that includes CRUK Therapeutic Discovery Laboratories and FORMA Therapeutics. M.G. was funded by the DUB Alliance. A.V.T. and B.G. are current or former employees of FORMA Therapeutics.

Received: August 15, 2018
Revised: December 20, 2018
Accepted: February 20, 2019
Published: March 26, 2019

REFERENCES

Adams, P.D., Afonine, P.V., Bunkóczi, G., Chen, V.B., Echols, N., Headd, J.J., Hung, L.-W., Jain, S., Kapral, G.J., Grosse Kunstleve, R.W., et al. (2011). The Phenix software for automated determination of macromolecular structures. Methods *55*, 94–106.

Bibby, J., Keegan, R.M., Mayans, O., Winn, M.D., and Rigden, D.J. (2012). AMPLE: a cluster-and-truncate approach to solve the crystal structures of small proteins using rapidly computed ab initio models. Acta Crystallogr. D Biol. Crystallogr. *68*, 1622–1631.

Bosch-Comas, A., Lindsten, K., Gonzàlez-Duarte, R., Masucci, M.G., and Marfany, G. (2006). The ubiquitin-specific protease USP25 interacts with three sarcomeric proteins. Cell. Mol. Life Sci. *63*, 723–734.

Clague, M.J., Barsukov, I., Coulson, J.M., Liu, H., Rigden, D.J., and Urbé, S. (2013). Deubiquitylases from genes to organism. Physiol. Rev. *93*, 1289–1315.

Clerici, M., Luna-Vargas, M.P.A., Faesen, A.C., and Sixma, T.K. (2014). The DUSP-Ubl domain of USP4 enhances its catalytic efficiency by promoting ubiquitin exchange. Nat. Commun. *5*, 5399.

Cremona, C.A., Sancho, R., Diefenbacher, M.E., and Behrens, A. (2016). Fbw7 and its counteracting forces in stem cells and cancer: oncoproteins in the balance. Semin. Cancer Biol. *36*, 52–61.

Cuella-Martin, R., Oliveira, C., Lockstone, H.E., Snellenberg, S., Grolmusova, N., and Chapman, J.R. (2016). 53BP1 integrates DNA repair and p53-dependent cell fate decisions via distinct mechanisms. Mol. Cell *64*, 51–64.

Diefenbacher, M.E., Popov, N., Blake, S.M., Schülein-Völk, C., Nye, E., Spencer-Dene, B., Jaenicke, L.A., Eilers, M., and Behrens, A. (2014). The deubiquitinase USP28 controls intestinal homeostasis and promotes colorectal cancer. J. Clin. Invest. *124*, 3407–3418.

Ekkebus, R., van Kasteren, S.I., Kulathu, Y., Scholten, A., Berlin, I., Geurink, P.P., de Jong, A., Goerdayal, S., Neefjes, J., Heck, A.J.R., et al. (2013). On terminal alkynes that can react with active-site cysteine nucleophiles in proteases. J. Am. Chem. Soc. *135*, 2867–2870.

Emsley, P., Lohkamp, B., Scott, W.G., and Cowtan, K. (2010). Features and development of Coot. Acta Crystallogr. D Biol. Crystallogr. *66*, 486–501.

Evans, P.R., and Murshudov, G.N. (2013). How good are my data and what is the resolution? Acta Crystallogr. D Biol. Crystallogr. *69*, 1204–1214.

Faesen, A.C., Dirac, A.M.G., Shanmugham, A., Ovaa, H., Perrakis, A., and Sixma, T.K. (2011). Mechanism of USP7/HAUSP activation by its C-terminal ubiquitin-like domain and allosteric regulation by GMP-synthetase. Mol. Cell *44*, 147–159.

Favier, A., and Brutscher, B. (2011). Recovering lost magnetization: polarization enhancement in biomolecular NMR. J. Biomol. NMR *49*, 9–15.

Gersch, M., Gladkova, C., Schubert, A.F., Michel, M.A., Maslen, S., and Komander, D. (2017). Mechanism and regulation of the Lys6-selective deubiquitinase USP30. Nat. Struct. Mol. Biol. *24*, 920–930.

Harhaj, E.W., and Dixit, V.M. (2012). Regulation of NF-κB by deubiquitinases. Immunol. Rev. *246*, 107–124.

Harrigan, J.A., Jacq, X., Martin, N.M., and Jackson, S.P. (2018). Deubiquitylating enzymes and drug discovery: emerging opportunities. Nat. Rev. Drug Discov. *17*, 57–78.

Holm, L., and Laakso, L.M. (2016). Dali server update. Nucleic Acids Res. *44* (W1), W351–W355.

Hu, M., Li, P., Li, M., Li, W., Yao, T., Wu, J.-W., Gu, W., Cohen, R.E., and Shi, Y. (2002). Crystal structure of a UBP-family deubiquitinating enzyme in isolation and in complex with ubiquitin aldehyde. Cell *111*, 1041–1054.

Kabsch, W. (2010). XDS. Acta Crystallogr. D Biol. Crystallogr. *66*, 125–132.

Keul, N.D., Oruganty, K., Schaper Bergman, E.T., Beattie, N.R., McDonald, W.E., Kadirvelraj, R., Gross, M.L., Phillips, R.S., Harvey, S.C., and Wood, Z.A. (2018). The entropic force generated by intrinsically disordered segments tunes protein function. Nature *563*, 584–588.

Lin, D., Zhang, M., Zhang, M.-X., Ren, Y., Jin, J., Zhao, Q., Pan, Z., Wu, M., Shu, H.-B., Dong, C., and Zhong, B. (2015). Induction of USP25 by viral infection promotes innate antiviral responses by mediating the stabilization of TRAF3 and TRAF6. Proc. Natl. Acad. Sci. USA *112*, 11324–11329.

Liu, B., Sureda-Gómez, M., Zhen, Y., Amador, V., and Reverter, D. (2018). A quaternary tetramer assembly inhibits the deubiquitinating activity of USP25. Nat. Commun. *9*, 4973.

McCoy, A.J., Grosse-Kunstleve, R.W., Adams, P.D., Winn, M.D., Storoni, L.C., and Read, R.J. (2007). Phaser crystallographic software. J. Appl. Cryst. *40*, 658–674.

Meulmeester, E., Kunze, M., Hsiao, H.H., Urlaub, H., and Melchior, F. (2008). Mechanism and consequences for paralog-specific sumoylation of ubiquitin-specific protease 25. Mol. Cell *30*, 610–619.

Mevissen, T.E.T., and Komander, D. (2017). Mechanisms of deubiquitinase specificity and regulation. Annu. Rev. Biochem. *86*, 159–192.

Michel, M.A., Komander, D., and Elliott, P.R. (2018). Enzymatic assembly of ubiquitin chains. Methods Mol. Biol. *1844*, 73–84.

Nicholls, R.A., Long, F., and Murshudov, G.N. (2012). Low-resolution refinement tools in REFMAC5. Acta Crystallogr. D Biol. Crystallogr. *68*, 404–417.

Özen, A., Rougé, L., Bashore, C., Hearn, B.R., Skelton, N.J., and Dueber, E.C. (2018). Selectively modulating conformational states of USP7 catalytic domain for activation. Structure *26*, 72–84.e7.

Popov, N., Wanzel, M., Madiredjo, M., Zhang, D., Beijersbergen, R., Bernards, R., Moll, R., Elledge, S.J., and Eilers, M. (2007). The ubiquitin-specific protease USP28 is required for MYC stability. Nat. Cell Biol. *9*, 765–774.

Pruneda, J.N., Durkin, C.H., Geurink, P.P., Ovaa, H., Santhanam, B., Holden, D.W., and Komander, D. (2016). The molecular basis for ubiquitin and ubiquitin-like specificities in bacterial effector proteases. Mol. Cell *63*, 261–276.

Rambo, R.P., and Tainer, J.A. (2013). Super-resolution in solution X-ray scattering and its applications to structural systems biology. Annu. Rev. Biophys. *42*, 415–441.

Rougé, L., Bainbridge, T.W., Kwok, M., Tong, R., Di Lello, P., Wertz, I.E., Maurer, T., Ernst, J.A., and Murray, J. (2016). Molecular understanding of USP7 substrate recognition and C-terminal activation. Structure *24*, 1335–1345.

Schneidman-Duhovny, D., Hammel, M., Tainer, J.A., and Sali, A. (2016). FoXS, FoXSDock and MultiFoXS: Single-state and multi-state structural modeling of proteins and their complexes based on SAXS profiles. Nucleic Acids Res. *44* (W1), W424–W429.

Serra, R.W., Fang, M., Park, S.M., Hutchinson, L., and Green, M.R. (2014). A KRAS-directed transcriptional silencing pathway that mediates the CpG island methylator phenotype. eLife *3*, e02313.

Tang, X., Orlicky, S., Lin, Z., Willems, A., Neculai, D., Ceccarelli, D., Mercurio, F., Shilton, B.H., Sicheri, F., and Tyers, M. (2007). Suprafacial orientation of the SCFCdc4 dimer accommodates multiple geometries for substrate ubiquitination. Cell *129*, 1165–1176.

Wang, X., Liu, Z., Zhang, L., Yang, Z., Chen, X., Luo, J., Zhou, Z., Mei, X., Yu, X., Shao, Z., et al. (2018). Targeting deubiquitinase USP28 for cancer therapy. Cell Death Dis. *9*, 186.

Wei, Y., Thompson, J., and Floudas, C.A. (2011). CONCORD: a consensus method for protein secondary structure prediction via mixed integer linear optimization. Proc. Royal Soc. A, Math. Phys. Eng. Sci. *468*, 20110514.

Wu, Y., Wang, Y., Yang, X.H., Kang, T., Zhao, Y., Wang, C., Evers, B.M., and Zhou, B.P. (2013). The deubiquitinase USP28 stabilizes LSD1 and confers stem-cell-like traits to breast cancer cells. Cell Rep. *5*, 224–236.

Xu, D., Liu, J., Fu, T., Shan, B., Qian, L., Pan, L., and Yuan, J. (2017). USP25 regulates Wnt signaling by controlling the stability of tankyrases. Genes Dev. *31*, 1024–1035.

Yang, Y., Shi, L., Ding, Y., Shi, Y., Hu, H.-Y., Wen, Y., and Zhang, N. (2017). Structural and functional investigations of the N-terminal ubiquitin binding region of Usp25. Biophys. J. *112*, 2099–2108.

CellPress

Ye, Y., Scheel, H., Hofmann, K., and Komander, D. (2009). Dissection of USP catalytic domains reveals five common insertion points. Mol. Biosyst. *5*, 1797–1808.

Zhang, D., Zaugg, K., Mak, T.W., and Elledge, S.J. (2006). A role for the deubiquitinating enzyme USP28 in control of the DNA-damage response. Cell *126*, 529–542.

Zhen, Y., Knobel, P.A., Stracker, T.H., and Reverter, D. (2014). Regulation of USP28 deubiquitinating activity by SUMO conjugation. J. Biol. Chem. *289*, 34838–34850.

Zhong, B., Liu, X., Wang, X., Chang, S.H., Liu, X., Wang, A., Reynolds, J.M., and Dong, C. (2012). Negative regulation of IL-17-mediated signaling and inflammation by the ubiquitin-specific protease USP25. Nat. Immunol. *13*, 1110–1117.

Zhong, B., Liu, X., Wang, X., Liu, X., Li, H., Darnay, B.G., Lin, X., Sun, S.-C., and Dong, C. (2013). Ubiquitin-specific protease 25 regulates TLR4-dependent innate immune responses through deubiquitination of the adaptor protein TRAF3. Sci. Signal. *6*, ra35.

STAR★METHODS

KEY RESOURCES TABLE

REAGENT or RESOURCE	SOURCE	IDENTIFIER
Antibodies		
Rabbit monoclonal anti-USP25	abcam	Cat. # ab187156
Rabbit polyclonal anti-USP28	proteintech	Cat. # 17707-1-AP
Mouse monoclonal anti-Flag M2	Sigma	Cat. # F3165
Mouse monoclonal anti-HA	BioLegend	Cat. # 16B12
Goat polyclonal anti-GFP	abcam	Cat. # ab6673
Mouse monoclonal anti-GFP JL-8	TaKaRa	Cat. # 632381
Mouse monoclonal anti-GAPDH	Ambion	Cat. # AM4300
Mouse monoclonal anti-Tubulin	Sigma	Cat. # T6199
Sheep anti-mouse IgG-HRP	Sigma	Cat. # GENXA931
Donkey anti-rabbit IgG-HRP	Sigma	Cat. # GENA934
Donkey anti-goat IgG-HRP	Santa Cruz Biotech	Cat. # sc-2020
Bacterial and Virus Strains		
E. coli TOP10	Thermo Fisher	Cat. # C404010
E. coli Rosetta2(DE3)pLacI	Merck	Cat. # 71404
Chemicals, Peptides, and Recombinant Proteins		
Ubiquitin-KG-TAMRA	P. Geurink, H. Ovaa (LUMC)	n/a
KG-TAMRA	P. Geurink, H. Ovaa (LUMC)	n/a
NT-495 dye	Nanotemper	Cat. # MO-L003
BSA	Pierce	Cat. # 23209
Polyethylenimine, Linear, MW 25000	Polysciences	Cat. # 23966
Critical Commercial Assays		
Phusion High-Fidelity DNA Polymerase	NEB	Cat. # M0530
Clarity Western ECL substrate	Bio-Rad	Cat. # 1705060
SuperSignal Femto Maximum Sensitivity Substrate	ThermoFisher	Cat. # 34094
Deposited Data		
Structure of USP28 (149-703)	This study	PDB: 6HEJ
Structure of USP28 (149-703)~Ub-PA	This study	PDB: 6HEK
Structure of USP28 (149-Δinsert-703)	This study	PDB: 6HEH
Structure of USP28 (149-Δinsert-703)~Ub-PA	This study	PDB: 6HEI
Structure of USP25 (157-714)	This study	PDB: 6HEL
Structure of USP25 (748-1048)	This study	PDB: 6HEM
Experimental Models: Cell Lines		
HEK293	ATCC	Cat. # CRL-1573
Recombinant DNA		
Codon-optimized human USP28 gene in pMK-RQ	This study	MG-22-21
Human USP28 (1-1077) in pOPINB	This study	MG-31-04
Human USP28 (1-Δinsert-1077) in pOPINB	This study	MG-31-05
Human USP28 (1-703) in pOPINB	This study	MG-31-02
Human USP28 (1-Δinsert-703) in pOPINB	This study	MG-31-03
Human USP28 (149-703) in pOPINB	This study	MG-26-50
Human USP28 (149-Δinsert-703) in pOPINB	This study	MG-26-54
Human USP28 (149-703) V541E L545E in pOPINB	This study	MG-31-12
Human USP28 (149-703) L415E I419E in pOPINB	This study	MG-31-15

(*Continued on next page*)

Continued

REAGENT or RESOURCE	SOURCE	IDENTIFIER
Human USP28 (149-Δ(459-528)-703) in pOPINB	This study	MG-31-20
Human USP25 (157-720) in pOPINB	This study	MG-31-70
Human USP25 (157-Δinsert-720) in pOPINB	This study	MG-31-72
Human USP25 (157-720) L548E L552E in pOPINB	This study	MG-31-71
Human USP25 (157-714) in pOPINB	This study	MG-32-75
Human USP25 (157-Δinsert-714) in pOPINB	This study	MG-32-95
Human USP25 (157-714) L548E L552E in pOPINB	This study	MG-32-96
Human USP25 (157-Δ(465-535)-714) in pOPINB	This study	MG-32-77
Human USP25 (1-1055) in pOPINB	This study	MG-32-71
Human USP25 (1-714) in pOPINB	This study	MG-32-72
Human USP25 (157-1055) in pOPINB	This study	MG-32-89
Human USP25 (1-Δ(465-535)-1055) in pOPINB	This study	MG-32-93
D. rerio USP28 (138-684) in pOPINB	This study	MG-38-47
G. gallus USP28 (143-706) in pOPINB	This study	MG-38-55
M. musculus USP25 (157-715) in pOPINB	This study	MG-38-53
G. gallus USP25 (174-726) in pOPINB	This study	MG-38-57
M. musculus USP25 (1-1055) in pOPINB	This study	MG-38-52
D. rerio USP28 (1-738,763-1187) in pOPINB	This study	MG-38-46
Human USP25 (157-714) P521S F522E in pOPINB	This study	MG-38-84
Human USP25 (157-714) P521A in pOPINB	This study	MG-38-85
Human USP25 (157-714) F522A in pOPINB	This study	MG-38-86
Human USP25 (157-714) F522L in pOPINB	This study	MG-38-87
Human USP25 (157-714) Q524S in pOPINB	This study	MG-38-88
Chimera: Human USP25 (157-[Insert of Human USP28]-714) in pOPINB	This study	MG-38-79
Chimera: Human USP28 (149-[Insert of Human USP25]-703) in pOPINB	This study	MG-38-80
Chimera: Human USP25 (157-[AIM of Human USP28]-714) in pOPINB	This study	MG-38-81
Chimera: Human USP28 (149-[AIM of Human USP25]-703) in pOPINB	This study	MG-38-82
Chimera: Huma USP25 (157-[AIM of *D. rerio* USP28]-714) in pOPINB	This study	MG-38-83
Human USP25 (748-1048) in pOPINB	This study	MG-31-80
Human USP28 (736-1077) in pOPINB	This study	MG-31-10
GFP in pOPINE	This study	MG-52-30
GFP-USP28 (2-1077) in pOPINE	This study	MG-52-31
GFP-USP28 (2-1077) V541E L545E in pOPINE	This study	MG-52-69
GFP-USP25 (2-1055) in pOPINE	This study	MG-52-64
GFP-USP25 (2-1055) P521S F522E in pOPINE	This study	MG-52-65
GFP-USP25 (2-1055) L548E L552E in pOPINE	This study	MG-52-68
Flag-USP25 (2-1055) in pOPINE	This study	MG-52-49
Flag-USP25 (2-1055) P521S F522E in pOPINE	This study	MG-52-50
Flag-USP25 (2-1055) L548E L552E in pOPINE	This study	MG-32-08
HA-USP28 (2-1077) in pOPINE	This study	MG-23-34
HA-USP28 (2-1077) V541E L545E in pOPINE	This study	MG-32-13
HA-USP28 (2-1077) L415E I419E in pOPINE	This study	MG-32-23
HA-USP28 (2-1077) C171A in pOPINE	This study	MG-32-21

(*Continued on next page*)

Continued		
REAGENT or RESOURCE	SOURCE	IDENTIFIER
HA-Tankyrase-2 (2-1166) in pOPINE	This study	MG-52-35
Flag-LSD1 (2-852) in pOPINE	This study	MG-52-33
Software and Algorithms		
XDS Version November 11, 2017	W. Kabsch (MPI Heidelberg)	http://homes.mpimf-heidelberg.mpg.de/∼kabsch/xds/
Autoproc	Global Phasing Ltd	https://www.globalphasing.com/
CCP4 7.0.060	CCP4 team	http://www.ccp4.ac.uk/
Phenix 1.13-2998	Phenix team	https://www.phenix-online.org/
Staraniso webserver	Global Phasing Ltd	http://staraniso.globalphasing.org
AMPLE	Bibby et al., 2012	http://pcwww.liv.ac.uk/∼drigden/
ScÅtter 3.0	R. Rambo (DLS Didcot)	http://www.bioisis.net/
Fast SAXS Profile Computation with Debye Formula webserver	Schneidman-Duhovny et. al. 2016	https://modbase.compbio.ucsf.edu/foxs/
Pymol v1.8.2.2	Schrödinger LLC	https://pymol.org/2/
CONCORD webserver	Wei et al., 2011	http://helios.princeton.edu/CONCORD/
ConSurf webserver	Biosof LLC	http://consurf.tau.ac.il/2016/
DALI webserver	Holm and Laakso, 2016	http://ekhidna2.biocenter.helsinki.fi/dali/
Pro-origami webserver	A. Stivala (U Melbourne)	http://munk.cis.unimelb.edu.au/pro-origami/
NetWheels	A. Mól, W. Fontes, M. Castro (U Brasília)	http://www.lbqp.unb.br/NetWheels/
ESPript 3.0	P. Gouet and X. Robert (Lyon)	http://espript.ibcp.fr/ESPript/ESPript/
Astra 6.1	Wyatt Technology	https://www.wyatt.com/products/software/astra
Topspin 3.1	Bruker	https://www.bruker.com
Image Lab	Bio-Rad	http://www.bio-rad.com/en-uk/product/image-lab-software?ID=KRE6P5E8Z
Illustrator CS6	Adobe	https://www.adobe.com/uk/products/illustrator.html

CONTACT FOR REAGENT AND RESOURCE SHARING

Requests for resources and reagents should be directed to and will be fulfilled by the lead contact, David Komander (dk@wehi.edu.au).

EXPERIMENTAL MODEL AND SUBJECT DETAILS

HEK293 cells (ATCC CRL-1573) were maintained in DMEM + GlutaMAX supplemented with 10% (v/v) fetal bovine serum (FBS) and penicillin-streptomycin at 37°C in a humidified atmosphere containing 5% CO_2. Cells were tested negative for mycoplasma contamination with a Lonza MycoAlert Assay.

METHOD DETAILS

Cloning and constructs

Human USP28 constructs were cloned from a gene obtained from GeneArt (Thermo Fisher) with codon-optimization for bacterial expression. Human USP25, LSD1 and TNKS2 were cloned from vectors kindly provided by Sylvie Urbe, Bradley Bernstein (Addgene plasmid #49042) and Marc de la Roche, respectively. Mouse, chicken and zebrafish orthologs were cloned from cDNA libraries.

Protein sequences correspond to Uniprot entries Q96RU2 (*H. sapiens* USP28), Q9UHP3 (*H. sapiens* USP25), P57080 (*M. musculus* USP25), Q5ZID5 (*G. gallus* USP28), F1NCR4 (*G. gallus* USP25), E7FD72 (*D. rerio* USP28), O60341 (*H. sapiens* LSD1) and Q9H2K2 (*H. sapiens* TNKS2).

Constructs were assembled using In-Fusion cloning (Clontech) into pOPINB (all constructs for bacterial expression) or pOPINE (constructs for transient transfection of mammalian cells with N-terminal tags as indicated) vectors and amplified in *E. coli* TOP10 cells (Thermo Fisher). Site-directed mutagenesis was carried out using the QuikChange method or with overlap extension PCR.

Boundaries were chosen according to secondary structure predictions using the Concord webserver (Wei et al., 2011). A triple GS linker was used to replace deleted sequences in constructs where a deletion is indicated.

GFP-tagged constructs were cloned with a monomeric (A206K) eGFP sequence.

Protein expression and purification

Expression was carried out in *E. coli* Rosetta2(DE3)pLacI cells (Merck) that were chemically transformed with expression vector and subsequently grown in 2xTY media supplemented with kanamycin (50 mg/L) and chloramphenicol (34 mg/L). Cultures were grown at 37°C for ~3-4 h until an OD_{600} of 1.0 and then cooled to 18°C for ~1 h. Expression was induced by addition of 0.5 mM isopropyl-β-D-1-thiogalactopyranoside (IPTG) and cultures were kept shaking at 18°C over night. Following harvest by centrifugation, cell pellets were flash-frozen in liquid nitrogen and stored at –80°C. ^{15}N-labeled protein was obtained from expression in M9(–) media supplemented with 1 g/L ^{15}N-ammonium chloride and as described otherwise.

All purification steps were carried out on Äkta Explorer, Äkta Purifier and Äkta Pure systems (GE Healthcare) at 4°C. Pellets were resuspended in buffer A (50 mM sodium phosphate pH 8.0, 300 mM NaCl, 4 mM β-mercaptoethanol, 20 mM imidazole) supplemented with DNaseI and lysozyme. After lysis by sonication (4 min, 55% power, 10 s on / 10 s off, Fisherbrand FB705 sonicator), the lysate was cleared by centrifugation (22 000 rpm, 30 min, 4°C), filtered, and loaded on 5 mL HisTrap FF columns. Elution was carried out with a gradient over 6 column volumes (CV) into buffer B (as buffer A, with 500 mM imidazole). Protein containing fractions were pooled, supplemented with 3C protease and dialyzed into buffer C (25 mM Tris pH 8.5, 50 mM NaCl, 5 mM DTT) for 2-24 h at 4°C.

For anion-exchange chromatography, samples were loaded onto a 6 mL Resource Q column equilibrated in buffer C and eluted with a 20 CV gradient into buffer D (25 mM Tris pH 8.5, 500 mM NaCl, 5 mM DTT). Peak fractions were pooled, concentrated via centrifugation and loaded onto HiLoad 16/600 Superdex 200 pg or Superdex 75 pg columns for size-exclusion chromatography in buffer E (20 mM Tris pH 8.0, 100 mM NaCl, 5 mM DTT) or buffer F (as buffer E, with 5% (v/v) glycerol). The purity of peak fractions was assessed by SDS-PAGE. Fractions were concentrated at 4°C and 3,200 xg in spin concentrators (10 kDa MWCO, Viva spin) and flash-frozen in liquid nitrogen. All protein concentrations were determined by absorption at 280 nm unless noted otherwise.

Covalent ubiquitin-propargylamine complexes

Ub(1-75)-PA and His_6-3C-Ub(1-75)-PA suicide probes were assembled as described previously (Gersch et al., 2017) and analyzed by intact protein mass spectrometry confirming > 95% purity (expected mass of the His_6-3C-Ub(1-75)-PA probe: 10 657 Da, mass found: 10 657 Da).

Covalent attachment of His_6-3C-Ub(1-75)-PA to USP25/USP28 proteins was carried out following the anion exchange step. A 1.3 eq molar excess of probe was spiked into the pooled elution fractions and allowed to react for 1–4 h at 4°C. Dialysis into buffer A was carried out and the sample was loaded onto a 5 mL HisTrap for affinity chromatography. Following elution with buffer B, the protein complex was treated with 3C protease for 1 h at 4°C and subjected to gel filtration chromatography into buffer E as described above.

Crystallization

Crystallization experiments were carried out in 96-well sitting-drop vapor diffusion plates in MRC format (Molecular Dimensions) at 18°C and set up using a mosquito HTS robot (TTP Labtech). Typical drop ratios of 200 nL + 200 nL and 500 nL + 500 nL (protein solution + reservoir solution) were used for coarse screening and fine screening, respectively, unless noted otherwise.

USP28 (149-703) was concentrated to 13.3 mg/mL in buffer F and crystallized in 0.8 M ammonium sulfate, 100 mM sodium citrate pH 5.5 as large cuboids (~200x100x40 μm^3). The crystal-containing drop was overlaid with an equal volume of cryo solution made of reservoir (three parts) and glycerol (one part) and incubated for 2 min. Crystals were then transferred into the pure cryo solution and harvested after another 2 min incubation.

USP28 (149-703)~Ub-PA was concentrated to 14.1 mg/mL in buffer F and crystallized in 8% (w/v) PEG 3350, 100 mM sodium citrate pH 5.4 and 200 mM ammonium acetate as large cuboids (250x140x100 μm^3). Cryoprotection was achieved by swiping the crystal quickly through a cryo solution containing (20% (v/v) PEG 400, 10% (w/v) PEG 3350, 100 mM sodium citrate pH 5.6, 200 mM ammonium acetate) followed by immediate vitrification in liquid nitrogen.

USP28 (149-Δinsert-703) was concentrated to 15 mg/mL in buffer E and crystallized in 12% (w/v) PEG 8000, 100 mM sodium chloride, 200 mM lithium sulfate, 100 mM MES pH 6.6 as large cubes. Crystals were cryoprotected with a solution containing the components of the reservoir and 25% (v/v) ethylene glycol.

USP28 (149-Δinsert-703)~Ub was concentrated to 25 mg/mL in buffer E and crystallized in 22% (w/v) PEG 3350, 300 mM potassium sodium tartrate, yielding small cuboid crystals (40x50x90 μm^3) growing to their maximal size within 7 days. Cryoprotection was achieved by transferring the crystals into a solution containing the components of the reservoir and 25% (v/v) ethylene glycol.

USP25 (157-714) was concentrated to 11.5 mg/mL in buffer E and crystallized in 12% (w/v) PEG 3350, 167 mM magnesium acetate as long rods (2000x100x100 μm^3). Crystals were harvested into a cryo solution containing 12% (w/v) PEG 3350, 150 mM magnesium acetate, 20 mM Tris pH 8.0, 25% (v/v) glycerol, incubated for 5 min and vitrified in liquid nitrogen.

USP25 (748-1048) was concentrated to 11.6 mg/mL in buffer E and crystallized in 20% (w/v) PEG 4000, 600 mM sodium chloride, 100 mM MES pH 6.5 as needles (300x50x30 μm^3). Crystals were cryoprotected by transferring them into a solution containing the components of the reservoir and 25% (v/v) glycerol.

Data collection, structure solution and refinement

Diffraction data were collected at 100 K at the Diamond Light Source (DLS), Harwell, UK on beamlines I02, I03 and I04-1, and at the European Synchrotron Radiation Facility (ESRF), Grenoble, France on beamlines ID29 and ID30B. Datasets leading to structures of USP28 (149-Δinsert-703), USP28 (149-Δinsert-703)~Ub-PA and USP25 (748-1048) were processed using XDS (Kabsch, 2010), and scaled using Aimless (Evans and Murshudov, 2013) in the CCP4 suite of programs. The datasets leading to the structures of USP28 (149-703), USP28 (149-703)~Ub-PA and USP25 (157-714) showed a high degree of anisotropy, and anisotropy correction was hence performed using the STARANISO web server (http://staraniso.globalphasing.org). For this purpose, the data were indexed and integrated in the respective space groups by XDS (USP28 (149-703)~Ub-PA and USP25 (157-714)) or autoproc (USP28 (149-703)) and the unmerged datasets were submitted to the STARANISO server. R_{free} flags were then added to the merged and ellipsoidally scaled output data using the Phenix reflection file editor. Anisotropy correction greatly improved the quality and interpretability of the electron density maps and was critical for converging refinement runs.

The structure of USP28 (149-Δinsert-703)~Ub-PA was solved by molecular replacement using MR Phaser (McCoy et al., 2007) and a chainsaw-derived search model of USP7 in complex with ubiquitin (PDB: 1NBF). Model building using Coot (Emsley et al., 2010) and refinement with Phenix.Refine (Adams et al., 2011) yielded the final structure that was used as a search model to solve the apo structure of USP28 (149-Δinsert-703).

The coordinates of the USP28 (149-Δinsert-703)~Ub-PA structure were then used as a search model to phase the data leading to the structure of USP28 (149-703)~Ub-PA. Two copies of the search model could be placed, and clear helical density extending away from the catalytic USP domain was visible. Several rounds of manual model building using Coot, refinement by Refmac (with external restraints against the high-resolution structure of USP28 (149-Δinsert-703)~Ub-PA generated by ProSmart (Nicholls et al., 2012)) and Phenix.Refine were used to arrive at the final model. The sequence register of the domain insertion could be assigned unambiguously due to the clear connectivity to the catalytic domain. The apo structure of USP28 (149-703) was solved by molecular replacement using two copies of the high resolution catalytic domain structure of USP28 (149-Δinsert-703) as well as one copy of the dimeric insertion sequence of the USP28 (149-703)~Ub-PA structure as search models.

The dataset leading to the structure of USP25 (157-714) was initially processed in spacegroup *I*422 and solved by molecular replacement using chainsaw models of the catalytic domain derived from the USP28 (149-Δinsert-703) structure and the insertion sequence of one copy of the USP28 (149-703)~Ub-PA structure. However, refinement stalled at an R_{free} of ~37% with little interpretable difference density. The dataset was hence reprocessed in space group *P*1 with anisotropy correction as described above. Space group validation runs were carried out with the Zanuda pipeline as implemented in the CCP4 suite of programs using models obtained from perturbation of the intermediate *I*422 model with Phenix.Dynamics. Consistently, automated refinement with Refmac resulted in a ~2%–3% high R_{free} in *I*422 compared to all other space groups (*C*2, *I*222, *F*222, *I*4). The final model was obtained from anisotropy-corrected data processed in spacegroup *I*4 and refined with Phenix.Refine to an R_{free} of 27.8%.

Secondary structure prediction of the USP25 C-terminal sequence suggested a high α-helical content. The structure of USP25 (748-1048) was subsequently solved by *ab initio* molecular replacement with 15 residue polyalanine helices as implemented in AMPLE (Bibby et al., 2012) with an automated pipeline using MR Phaser for generating initial phases, SHELXE for phasing and density modification, Refmac for refinement and ArpWarp for model building, yielding a near-finished model with an R_{free} of 26%. Further rounds of refinement were carried out with Phenix.Refine to arrive at the final model with an R_{free} of 20.9%.

Data collection, anisotropy correction and refinement statistics are given in Table 1.

Small-angle X-ray scattering

SAXS analysis was carried out at beamline B21 at Diamond Light Source, Harwell, UK. Samples (45 μL at 3-12 mg/mL) were subjected to size exclusion chromatography on a Shodex KW-403 column in buffer E and subsequent SAXS analysis. The program ScÅtter was used for data analysis (http://www.bioisis.net, Robert P. Rambo, Diamond Light Source). Peak frames were averaged and background-corrected against frames of the same run. Guinier fits were carried out to determine *I*(0) and R_g by truncating the data at the low *q* range to satisfy the $q \times R_g$ requirement of > 1.30. Guinier plots were linear and confirmed the absence of aggregates. Data from ~0.013 $Å^{-1} < q <$ ~0.22 $Å^{-1}$ extrapolated to zero angle were used for real space analysis. D_{max} was fit manually to the *P*(*r*) distributions to obtain good agreement between real space and reciprocal space derived *I*(0) and R_g values. All samples were measured in duplicate with near identical results. The FoXS webserver (Schneidman-Duhovny et al., 2016) was used for the calculation of a theoretical scattering profile of structures and its fitting to an experimental profile (χ^2 values are given in the figures). See Table S1 for parameters derived from SAXS data analysis.

Fluorescence polarization assays

DUB activity was quantified by fluorescence polarization assays using Ub-KG-TAMRA or Lys48-diUb-FlAsH (Pruneda et al., 2016) substrates. Kinetic experiments were carried out in black, round-bottom, non-binding surface 384-well plates (Greiner) at 25°C in PBS (phosphate-buffered saline, pH 7.4) supplemented with 5 mM DTT and 0.05 mg/mL BSA with 0.1 μM substrate concentration and indicated enzyme concentrations in 20 μL reactions. Data were recorded on a PheraStar plate reader (BMG Labtech), equipped with an optic module using 540 nm/590 nm (TAMRA) and 485 nm/520 nm (FlAsH) filter pairs for excitation and emission, respectively. For TAMRA assays, typically one read per min over 60 min was recorded, whereas for FlAsH assays one read per 20-30 s was recorded to cover the steep decline in polarization at high enzyme concentrations. Polarization values of 50 mP for free KG-TAMRA

and 160 mP for Ub-KG-TAMRA were determined in a cuvette-based spectrofluorometer and used as reference before conversion into anisotropies (mA). Data were analyzed using Microsoft Excel and Graphpad Prism and catalytic efficiencies were derived as described previously (Gersch et al., 2017). All experiments were performed with technical triplicates and in at least two independent experiments.

Gel-based ubiquitin-chain cleavage assay

Cleavage of Lys48-tetraUb (assembled from wild-type ubiquitin as reported by Michel et al., 2018) was followed by SDS-PAGE and Coomassie staining. 100 μL reactions were set up by mixing 50 μL of USP25 (2x: 0.6 μM) and 50 μL of Lys48-tetraUb (2x: 0.17 mg/mL, 5 μM) in 20 mM Tris pH 8.0, 100 mM sodium chloride and 5 mM DTT. Aliquots were taken at several time points by mixing 20 μL with 5 μL of LDS sample buffer, of which 10 μL were run on a gel. Samples were preequilibrated at 37°C, and reactions were incubated 37°C.

SEC-MALS

Size-exclusion multi-angle light scattering (SEC-MALS) analysis was carried out using an Agilent 1200 Series chromatography system coupled to a DAWN Heleos II multi-angle light scattering detector as well as an Optilab rEX refractive index detector (Wyatt Technology). Samples (100 μL of 1-2 mg/mL protein solutions unless noted otherwise) were subjected to size-exclusion chromatography on a Superdex 200 10/300 column run at a flow of 0.5 mL/min in PBS supplemented with 2 mM DTT. Masses and errors were derived from analysis in Astra 6.1 (Wyatt Technology) following calibration with BSA.

Fluorescence-detection size-exclusion chromatography

USP28(149-703)~Ub-PA was buffer-exchanged into PBS on a Superdex 200 10/300 column, concentrated via centrifugation and fluorescently labeled with NHS-functionalized dye NT-495 (MO-L003, Nanotemper, Munich). Free dye was removed by gel filtration into PBS + 2 mM DTT. Comparison of the peak profiles was used to confirme that labeling did not interfere with dimerization. Protein concentration and labeling efficiency (90%) were determined through absorption measurements at 280 nm and 493 nm, respectively. Fluorescence-detection size-exclusion chromatography (F-SEC) analysis was carried out on an Äkta Purifier coupled to an AUC-905 autosampler, a Superdex 200 Increase 3.2/300 column and a Hitachi 5440 FL detector (Excitation: 493 nm, Emission: 521 nm) in PBS + 2 mM DTT running at 0.05 mL/min with 25 μL injections of sample. PMT voltage and averaging time were varied to ensure the dynamic range of the detector was used. Curves were scaled in signal intensity with regard to the integral of the peak according to the injected amount of protein for comparison of all samples on one a.u. scale.

Thermal shift assay

Thermal shift assays were carried out on a Corbett RG-6000 real time PCR cycler (30°C to 85°C with 7 s per 0.5°C). Samples contained 4 μM protein and 4x Sypro Orange dye in PBS supplemented with 5 mM DTT. Protein melting curves were obtained as the maxima of d*F*/d*T* versus *T* plots. All data were recorded with 10 technical replicates and were consistent accross two independent experiments.

NMR spectroscopy

All NMR data were collected at 298 K using Bruker Avance spectrometers with ^{1}H resonance frequencies of 600 or 800 MHz fitted with ^{1}H{^{13}C,^{15}N} triple-resonance cryoprobes with 5% D_2O added to each sample as a lock solvent. ^{1}H-^{15}N BEST–TROSY (band selective excitation short transients transverse relaxation optimized spectroscopy) experiments were collected as standard ^{1}H,^{15}N-2D correlation experiments with an in-house optimized pulse sequence (Favier and Brutscher, 2011). Data used for the overlay of 2D spectra for binding tests were recorded with ^{15}N-labeled samples at 75 μM to which equimolar amounts of a different, unlabeled protein were added where indicated. Samples were prepared in PBS + 4 mM DTT. All spectra were processed using the program Topspin 3.1 and analyzed using the program Sparky 3.115.

Native PAGE, in-gel GFP fluorescence and immunoblotting analysis

HEK293 cells (ATCC CRL-1573) were maintained in DMEM + GlutaMAX supplemented with 10% (v/v) fetal bovine serum (FBS) and penicillin-streptomycin at 37°C in a humidified atmosphere containing 5% CO_2. Cells were tested negative for mycoplasma contamination with a Lonza MycoAlert Assay. On day one, cells were seeded in 6 well dishes (0.6 – 1 million cells / well). On day two, cells were transfected with 3 μg vector and 9 μg PEI premixed in 200 μL OPTI-MEM. On day three, cells were washed with ice-cold PBS and lysed in lysis buffer (150 μL/well) containing 50 mM Tris pH 7.5, 150 mM sodium chloride, 2 mM EDTA, 10% (w/v) glycerol, 0.8% (v/v) NP-40, protease inhibitors (Complete EDTA-free protease inhibitor tablets, Roche), 4 mM DTT and 10 mM beta-mercaptoethanol. After incubation for 10 min at 4°C, 150 μL/well lysis buffer supplemented with 10 mM magnesium chloride and 1:1000 benzonase (Merck) were added. Following clearance by centrifugation (14,000 xg, 10 min, 4°C), protein concentrations of the supernatants were measured by Bradford assay and BSA as standard. Whole cell lysate samples for SDS-PAGE analysis were obtained by mixing supernatant and 4x LDS sample buffer.

For native PAGE analysis, lysates were mixed 1:1 with 2x native PAGE sample buffer (200 mM Tris pH 8.6, 20% (v/v) glycerol, 0.005% (w/v) bromophenol blue) and promptly analyzed. Proteins were separated on NuPAGE 3%–8% Tris-Acetate protein gels

(1.0 mm, ThermoFisher) with 25 mM Tris, 192 mM glycine, pH 8.2-8.4 as running buffer at constant 150 V for 110 min at room temperature. For the measurement of in-gel GFP fluorescence, gels were imaged on an Amersham Typhoon Biomolecular Imager using the Cy2 channel (GE Healthcare). For analysis by immunoblotting, gels were incubated for 10 min in native PAGE running buffer supplemented with 0.1% (w/v) SDS with gentle agitation. Proteins were then transferred to a nitrocellulose membrane using the Trans-Blot Turbo system (Bio-Rad). Membranes were blocked in a 5% (w/v) milk solution in PBS-T (PBS + 0.1% (v/v) Tween-20) for 20 min and incubated for 1 h at room temperature with a primary antibody recognizing either USP25 (abcam, ab187156, 1:1000), Flag (Sigma, F3165, 1:2000), USP28 (proteintech, 17707-1-AP, 1:1000), HA (BioLegend, 16B12, 1:1000), GFP (abcam, ab6673, 1:1000), GAPDH (Ambion, AM4300, 1:10000) or Tubulin (Sigma, T6199, 1:4000). All antibodies were used in 3% (w/v) BSA in PBS-T with 0.02% (w/v) sodium azide. Membranes were subsequently incubated with donkey anti-rabbit IgG-HRP (Sigma, GENA934, 1:5000), sheep anti-mouse IgG-HRP (Sigma, GENXA931, 1:5000) or donkey anti-goat IgG-HRP (Santa Cruz Biotech, sc-2020, 1:5000) in blocking solution for 1 h at room temperature. Blots were developed with Clarity Western ECL substrate (Bio-Rad) or Amersham Western Blotting Detection Reagent (GE Healthcare), and imaged using a ChemiDoc MP Imaging System (Bio-Rad).

As a customized size marker, we used recombinant full-length human USP25 analyzed on a HiLoad 16/600 Superdex 200 pg column in buffer E immediately before the experiment. Fractions corresponding to the tetrameric and dimeric oligomerization states (see Figure S6A) were concentrated to 1.0 mg/mL, and samples were prepared as described above for native PAGE analysis. Blots were stained by Ponceau S solution after the transfer and imaged, followed by the blocking step. Ponceau S images and immunoblots were aligned based on the membrane contours (see Figure S8).

For co-transfection experiments, cells were transfected with 0.6 μg/well of Flag-LSD1 or HA-TNKS2 vector, and up to 3 μg/well of vector of USP28 or USP25, respectively, topped up to 3 μg with empty vector where appropriate. Samples were harvested as described above and analyzed by SDS-PAGE and immunoblotting.

QUANTIFICATION AND STATISTICAL ANALYSIS

Statistical details of the experiments can be found in the figure legends and in the figures. Data are given as mean ± standard deviation (SD) or mean ± standard error (SEM) as defined in the legend.

DATA AND SOFTWARE AVAILABILITY

Protein structures have been deposited with the protein data bank under PDB: 6HEH, 6HEI, 6HEJ, 6HEK, 6HEL, 6HEM.

Molecular Cell, Volume *74*

Supplemental Information

Distinct USP25 and USP28 Oligomerization States Regulate Deubiquitinating Activity

Malte Gersch, Jane L. Wagstaff, Angela V. Toms, Bradford Graves, Stefan M.V. Freund, and David Komander

Supplemental Figure 1

A

USP DUBs			identity	similarity
USP12	USP46		88%	96%
USP18	USP41		79%	83%
USP5	USP13		58%	82%
USP44	USP49		58%	78%
USP20	USP33		56%	78%
**USP25**	**USP28**		**48%**	**76%**
USP6	USP32		56%	68%
USP16	USP45		36%	66%
USP35	USP38		40%	66%
USP9X	USP24		27%	58%
USP2	USP21		35%	57%
USP26	USP29	USP37	29%	56%
USP4	USP11	USP15	33%	56%
USP31	USP43		33%	54%
USP36	USP42		28%	49%
USP22	USP27	USP51	39%	49%

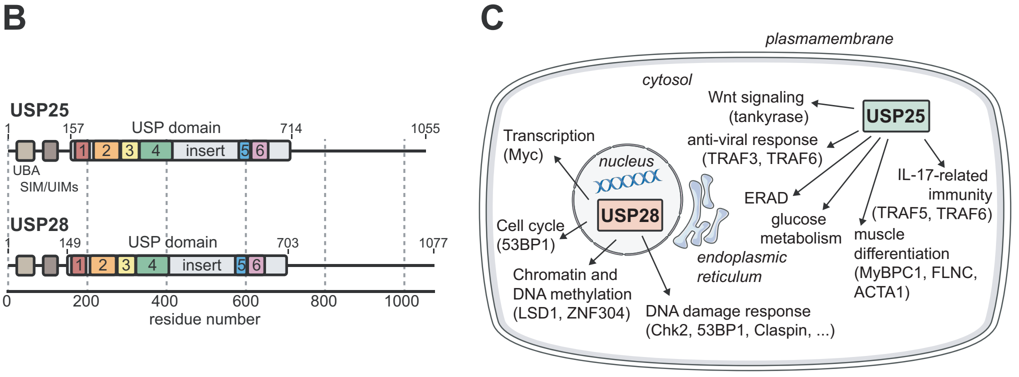

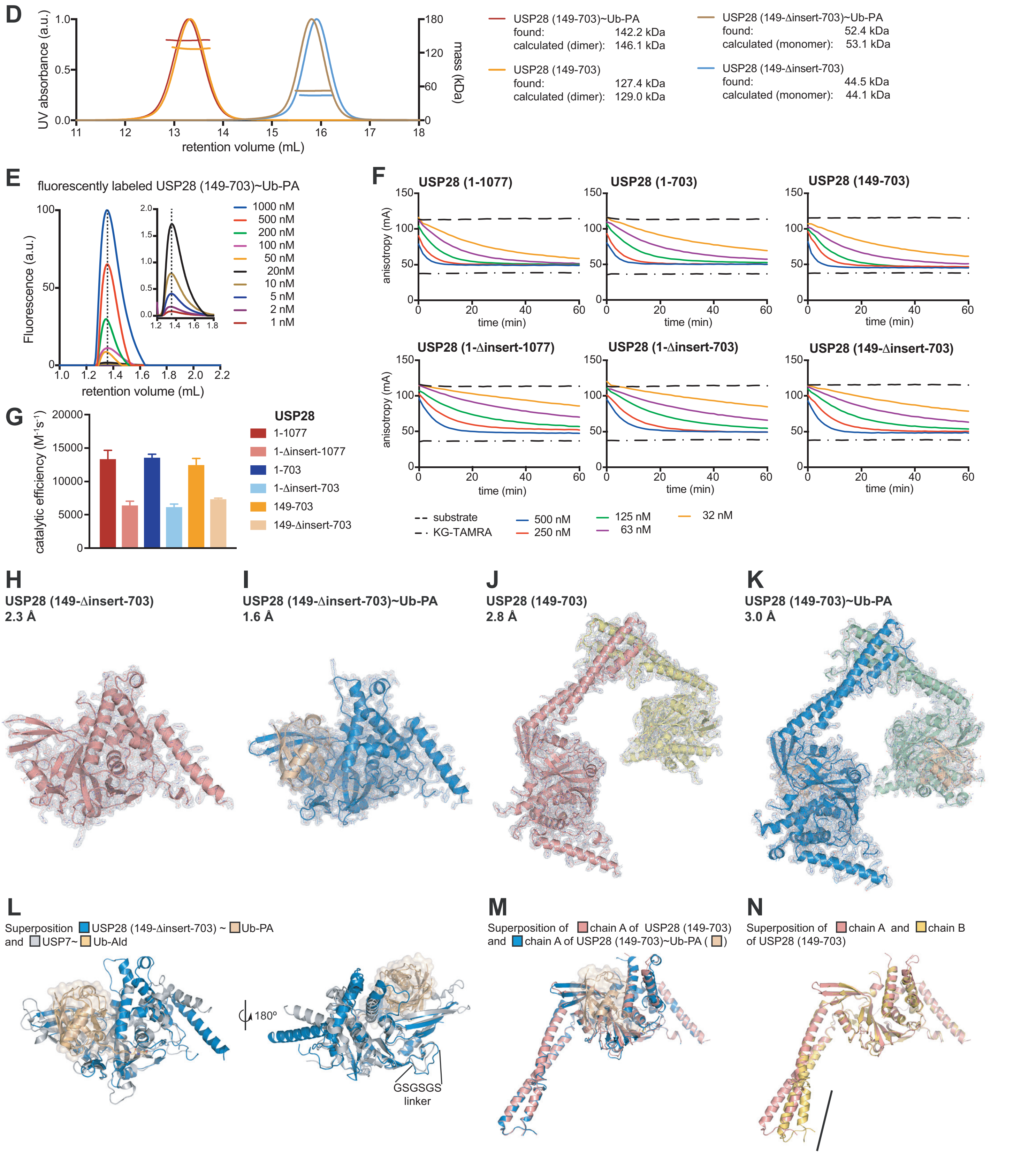

Figure S1. Cellular roles for USP25 and USP28. Biochemical and structural analysis of human USP28 proteins. Related to Figure 1.

(A) List of pairs or triplets of highly homologous human USPs DUBs. The percentage of identical residues as well as identical and similar residues is given. Data are based on alignments of full length protein sequences carried out with the Uniprot webserver. USP25 and USP28 are highlighted in bold.

(B) Schematic representation of human USP25 and USP28 constructs. The catalytic domain is shown in grey, an N-terminal UBA domain, SUMO-interaction motif (SIM) and ubiquitin-interaction motifs (UIMs) are shown in brown. Colored boxes indicate the conserved boxes characteristic of USP domains (Ye et al., 2009).

(C) Previously identified cellular roles linked to USP25 and USP28 indicated by arrows. Subcellular compartments and structures labeled in italics.

(D) SEC-MALS analysis of indicated constructs either in the apo form or bound to Ub-PA. Data of samples in the apo form are as shown in Figure 1C for easy comparison. Identified masses are matched to either monomeric or dimeric expected masses.

(E) A dilution series of a fluorescently labeled USP28 (149-703)~Ub-PA protein sample was analyzed on a size-exclusion chromatography system fitted with a fluorescence detector. Data collected on a more sensitive detector setting are shown as an inlet figure. The same central peak position is indicated with dotted lines in both graphs. The given concentrations correspond to the injected sample concentrations.

(F) Representative fluorescence anisotropy-time traces of Ub-KG-TAMRA cleavage assays from averaged technical triplicates of indicated USP28 constructs and concentrations.

(G) Catalytic activities of USP28 constructs determined from data shown in F. Data are shown as mean ± standard deviation from 2-5 independent experiments, each collected from technical triplicates.

(H, I, J and K) Overall views of the asymmetric units of indicated crystal structures (see Figure 1D-G for cartoon representations). Residues are shown as lines and in cartoon representation. Overlaid electron density maps in grey correspond to weighted $2|F_O|–|F_C|$ electron density contoured at 1 σ.

(L) Superposition of the Ub-PA-bound structure of USP28 (149-∆insert-703) and USP7 bound to ubiquitin-aldehyde (PDB-ID: 1NBF).
(M) Superposition of the apo and Ub-PA-bound structures of USP28 (149-703).
(N) Superposition of the two chains forming the asymmetric unit of the apo structure of USP28 (149-703). Notable differences in the insertion sequence are highlighted with a black line.

Supplemental Figure 2

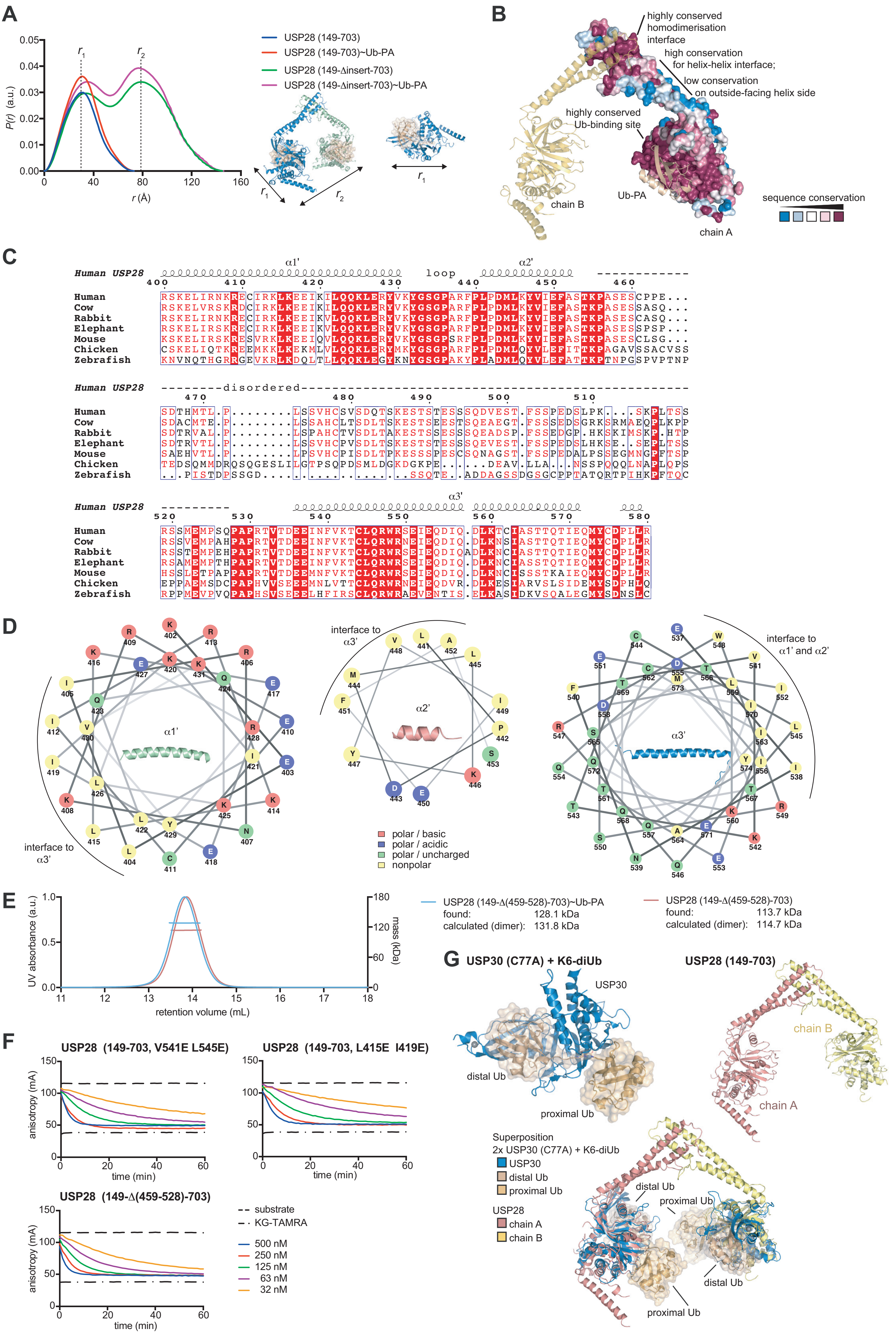

Figure S2. Structural analysis of the USP28 insertion sequences. Related to Figure 2.

(A) Pair-wise distance distribution functions *P(r)* calculated from SAXS data shown in Figure 2A for indicated constructs. The most frequent distances are highlighted with dashed lines and labeled r_1 and r_2. These are similar to the radius of gyration of the isolated USP domain (r_1) as well as the distance between the two USP domains in the dimer (r_2).

(B) Sequence conservation calculated from 96 USP28 protein sequences annotated as USP28 in the Uniprot database mapped as colored surface on chain A of the USP28 (149-703) structure. Chain B as well as the position of Ub-PA of the USP28 (149-Δinsert-703)~Ub-PA complex structure are shown as semi-transparent cartoons.

(C) Sequence alignment of representative USP28 members of vertebrate families for the insertion sequence. Secondary structure and disorder assignments were carried out with respect to the USP28 (149-703) structure.

(D) Helical wheel diagrams for the three helices α1', α2' and α3' of the USP28 insertion generated with the NetWheels webserver. Residues are colored according to their chemical nature as indicated. Interfaces are labeled and highlighted with black arcs. Cartoon representation of the respective helices as shown in Figure 2C.

(E) SEC-MALS analysis of USP28 catalytic domain constructs where the disordered part of the insertion is deleted (149-Δ(459-528)-703).

(F) Representative fluorescence anisotropy-time traces of Ub-KG-TAMRA cleavage assays from averaged technical triplicates of indicated USP28 constructs and concentrations. Catalytic efficiencies are reported in Figure 2E.

(G) Cartoon representation of inactive USP30 bound to K6-linked diUb (PDB-ID: 5OHP), with distal and proximal ubiquitin moieties labeled and also shown as transparent surfaces (*top left*). Cartoon representation of USP28 (149-703) (*top right*). Superposition of two copies of the USP30 complex on each of the catalytic domains of USP28 (*bottom*).

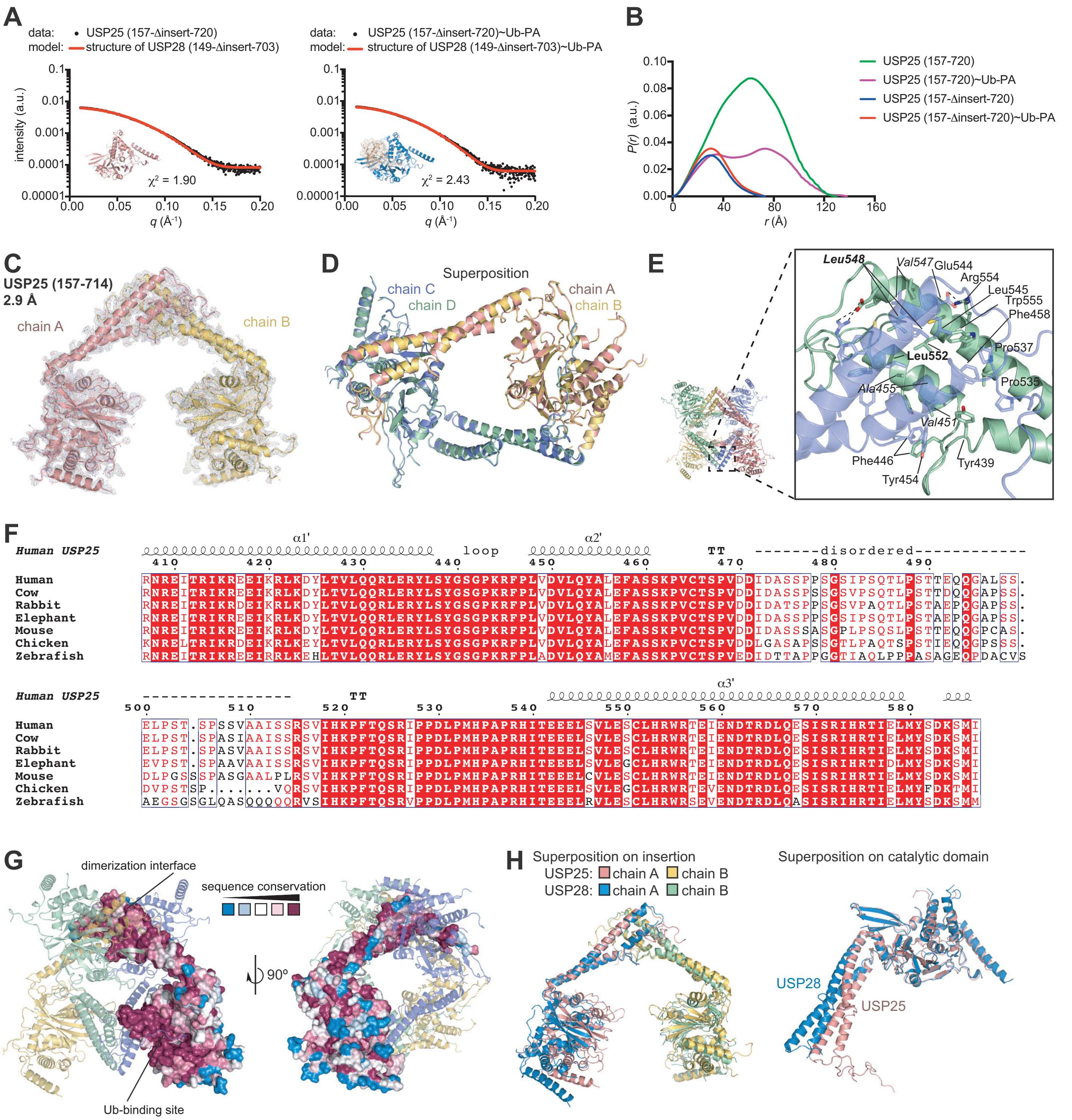
A
data: • USP25 (157-Δinsert-720)
model: structure of USP28 (149-Δinsert-703)
intensity (a.u.)
χ² = 1.90
q (Å⁻¹)
data: • USP25 (157-Δinsert-720)~Ub-PA
model: structure of USP28 (149-Δinsert-703)~Ub-PA
χ² = 2.43
q (Å⁻¹)
B
P(r) (a.u.)
r (Å)
USP25 (157-720)
USP25 (157-720)~Ub-PA
USP25 (157-Δinsert-720)
USP25 (157-Δinsert-720)~Ub-PA
C
USP25 (157-714)
2.9 Å
chain A
chain B
D
Superposition
chain C
chain D
chain A
chain B
E
Leu548
Val547
Glu544
Arg554
Leu545
Trp555
Phe458
Pro537
Leu552
Ala455
Pro535
Val451
Phe446
Tyr439
Tyr454
F
Human USP25
α1'
loop
α2'
TT
-------disordered------------
Human RNREITRIKREEIKRLKDYLTVLQQRLERYLSYGSGPKRFPLVDVLQYALEFASSKPVCTSPVDDIDASSPPSGSIPSQTLPSTTEQQGALSS.
Cow RNREITRIKREEIKRLKDYLTVLQQRLERYLSYGSGPKRFPLVDVLQYALEFASSKPVCTSPVDDIDASSPSSGSVPSQTLPSTTDQQGAPSS.
Rabbit RNREITRIKRDEIKRLKDYLTVLQQRLERYLSYGSGPKRFPLVDVLQYALEFASSKPVCTSPVDDIDATSPPSGSVPAQTLPSTAEPQGAPSS.
Elephant RNREITRIKREEIKRLKDYLTVLQQRLERYLSYGSGPKRFPLVDVLQYALEFASSKPVCTSPVDDIDASSPPSGSIPSQTLPSTAEPQGAPSS.
Mouse RNREITRIKREEIKRLKDYLTVLQQRLERYLSYGSGPKRFPLVDVLQYALEFASSKPVCTSPVDDIDASSSASGPLPSQSLPSTTEQQGPCAS.
Chicken KNRELTRIKRDEIKRLKEYLTVLQQRLERYLSYGSGPKRFPLVDVLQYALEFASSKPVCTSPVDDLGASAPSSGTLPAQTSPSTIEQQGPSSS.
Zebrafish RNREITRIKREEIRRLKEHLTVLQQRLERYLSYGSGPKRFPLADVLQYAMEFASSKPVCTSPVEDIDTTAPPGGTIAQLPPPASAGEQPDACVS
Human USP25
α3'
Human ELPST.SPSSVAAISSRSVIHKPFTQSRIPPDLPMHPAPRHITEEELSVLESCLHRWRTEIENDTRDLQESISRIHRTIELMYSDKSMI
Cow ELPST.SPASIAAISSRSVIHKPFTQSRIPPDLPMHPAPRHITEEELSVLESCLHRWRTEIENDTRDLQESISRIHRTIELMYSDKSMI
Rabbit ELPST.SPASVAAISSRSVIHKPFTQSRIPPDLPMHPAPRHITEEELSVLESCLHRWRTEIENDTRDLQESISRIHRTIELMYSDKSMI
Elephant EVPST.SPAAVAAISSRSVIHKPFTQSRIPPDLPMHPAPRHITEEELSVLEGCLHRWRTEIENDTRDLQESISRIHRTIDLMYSDKSMI
Mouse DLPGSSSPASGAALPLRSVIHKPFTQSRIPPDLPMHPAPRHITEEELCVLESCLHRWRTEIENDTRDLQESISRIHRTIELMYSDKSMI
Chicken DVPSTSP.......VQRSVIHKPFTQSRIPPDLPMHPAPRHITEEELSVLEGCLHRWRTEVENDTRDLQESISRIHRTIELMYFDKTMI
Zebrafish AEGSGSGLQASQQQQQRVSIHKPFTQSRVPPDLPMHPAPRHITEEELRVLESCLHRWRSEVENDTRDLQASISRIHRTIELMYSDKSMM
G
dimerization interface
sequence conservation
90°
Ub-binding site
H
Superposition on insertion
USP25: chain A chain B
USP28: chain A chain B
Superposition on catalytic domain
USP28
USP25

Figure S3. Structural and biochemical analysis of the catalytic domain of USP25. Related to Figure 3.

(A) Small angle X-ray scattering (SAXS) data shown as black dots which was collected from indicated USP25 protein samples. Expected scattering curves are shown in red that were calculated from solved USP28 X-ray crystal structures shown as small inlets.

(B) Pair-wise distance distribution functions *P(r)* calculated from SAXS data shown in Figures 3C and S3A for indicated constructs.

(C) Overall views of the asymmetric unit of the USP25 crystal structure. Residues are shown as lines and in cartoon representation. The overlaid electron density map in grey corresponds to weighted $2|F_O|–|F_C|$ electron density contoured at 1 σ.

(D) Superposition of chain A on top of chain B forming one asymmetric unit. A superposition of symmetry-related chains C (corresponding to A) and D (corresponding to B) is also shown.

(E) Close-up view of the indicated dimerization interface of USP25 chains C and D (compare to Figure 2B on the right for the respective USP28 interaction). Relevant residues are shown as sticks. Residues mutated to generate monomeric USP25 are labeled in bold, residues characteristically different from the respective USP28 interface are labeled in italics.

(F) Sequence alignment of representative USP25 members of vertebrate families for the insertion sequence. Secondary structure and disorder assignments were carried out with respect to the USP25 catalytic domain structure.

(G) Sequence conservation calculated from USP25 protein sequences mapped as colored surface on chain A of the USP25 (157-714) structure in two orientations. Chains B, C and D are shown as semi-transparent cartoons.

(H) Superpositions of cartoon representations of the crystal structures of USP25 and USP28. Shown are an alignment of dimeric forms (*left*) and individual catalytic domains (*right*).

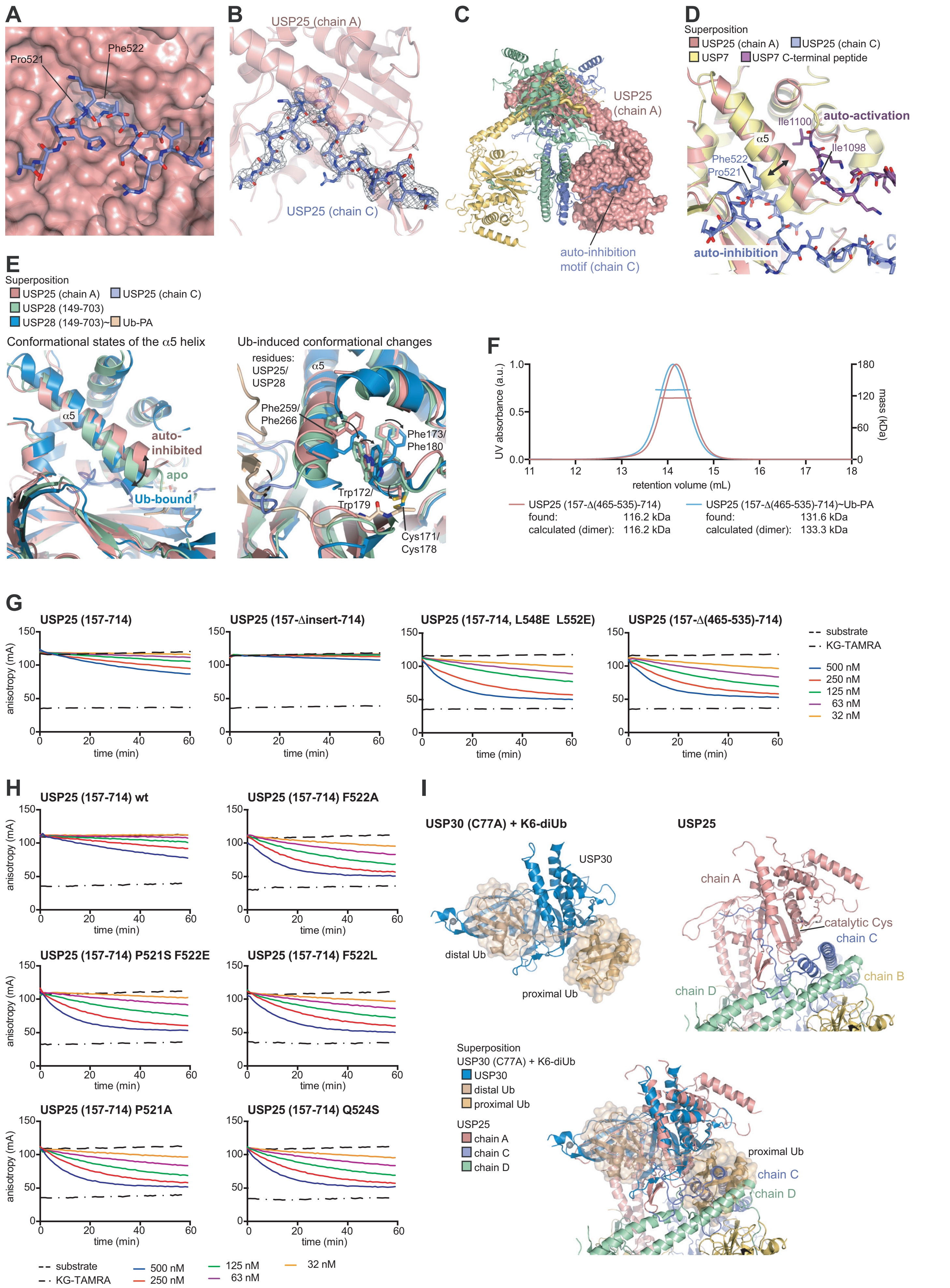
A
Pro521
Phe522
B
USP25 (chain A)
USP25 (chain C)
C
USP25
(chain A)
auto-inhibition
motif (chain C)
D
Superposition
USP25 (chain A)
USP25 (chain C)
USP7
USP7 C-terminal peptide
Ile1100
auto-activation
α5
Ile1098
Phe522
Pro521
auto-inhibition
E
Superposition
USP25 (chain A)
USP25 (chain C)
USP28 (149-703)
USP28 (149-703)~Ub-PA
Conformational states of the α5 helix
α5
auto-
inhibited
apo
Ub-bound
Ub-induced conformational changes
residues:
USP25/
USP28
α5
Phe259/
Phe266
Phe173/
Phe180
Trp172/
Trp179
Cys171/
Cys178
F
UV absorbance (a.u.)
mass (kDa)
retention volume (mL)
USP25 (157-Δ(465-535)-714)
found: 116.2 kDa
calculated (dimer): 116.2 kDa
USP25 (157-Δ(465-535)-714)~Ub-PA
found: 131.6 kDa
calculated (dimer): 133.3 kDa
G
USP25 (157-714)
USP25 (157-Δinsert-714)
USP25 (157-714, L548E L552E)
USP25 (157-Δ(465-535)-714)
anisotropy (mA)
time (min)
substrate
KG-TAMRA
500 nM
250 nM
125 nM
63 nM
32 nM
H
USP25 (157-714) wt
USP25 (157-714) F522A
USP25 (157-714) P521S F522E
USP25 (157-714) F522L
USP25 (157-714) P521A
USP25 (157-714) Q524S
anisotropy (mA)
time (min)
substrate
KG-TAMRA
500 nM
250 nM
125 nM
63 nM
32 nM
I
USP30 (C77A) + K6-diUb
USP30
distal Ub
proximal Ub
USP25
chain A
catalytic Cys
chain C
chain B
chain D
Superposition
USP30 (C77A) + K6-diUb
USP30
distal Ub
proximal Ub
USP25
chain A
chain C
chain D
proximal Ub
chain C
chain D

Figure S4. Mechanism of conserved auto-inhibition through oligomerization in USP25. Related to Figure 4.

(A) Close-up view of the pocket within the catalytic domain of USP25 (shown as red surface) that engages the auto-inhibition motif (shown as blue sticks).

(B) Electron density map in grey of the autoinhibition-motif contoured at 0.8 σ corresponding to weighted $2|F_O|–|F_C|$ electron density. The USP25 catalytic domain is shown as a semitransparent cartoon.

(C) Cartoon representation of tetrameric USP25 as in Figure 3D. Chain A is shown as a surface to highlight binding of the AIM of chain C to the catalytic domain of chain A.

(D) Superposition of cartoon representations of USP25 (red) and USP7 (yellow, PDB-ID: 5JTJ) in complex with the auto-inhibition motif (blue) and the USP7 C-terminal activating peptide (purple), respectively. Their binding to opposite sites of the α5 helices of the catalytic domains and the diverging α5 conformations are highlighted by a black arrow. Key hydrophobic residues in both peptidic motifs are labeled.

(E) Superposition of indicated crystal structures in cartoon representation showing the different positioning of the α5 helix in the catalytic USP domains depending on auto-inhibition or ubiquitin binding (*left*). A putative conformational relay mechanism links the positioning of the α5 helix through the sidechain conformation of Phe259 (USP25) / Phe266 (USP28) to the catalytic cysteine and its following aromatic residues (*right*). Conformational changes are highlighted with black arrows. The cartoon representation of ubiquitin is not shown in the left panel for clarity.

(F) SEC-MALS analysis of USP25 constructs in which the sequence corresponding to the part that is disordered in USP28 is deleted (construct corresponds to USP28 (149-Δ(459-528)-703), see Figure S2F).

(G and H) Representative fluorescence anisotropy-time traces of Ub-KG-TAMRA cleavage assays of indicated USP25 proteins and concentrations. Catalytic efficiencies are shown in Figure 4D and 4F.

(I) Cartoon representation of inactive USP30 bound to K6-linked diubiquitin (PDB-ID: 5OHP), with distal and proximal ubiquitin moieties labeled and also shown as transparent surfaces (*top left*). Cartoon representation of USP25 (157-714) (*top right*). Superposition of both structures on the USP domain of chain A of USP25,

showing the clashing of the proximal ubiquitin moiety in its putative position with chains C and D from the second USP25 dimer (*bottom*).

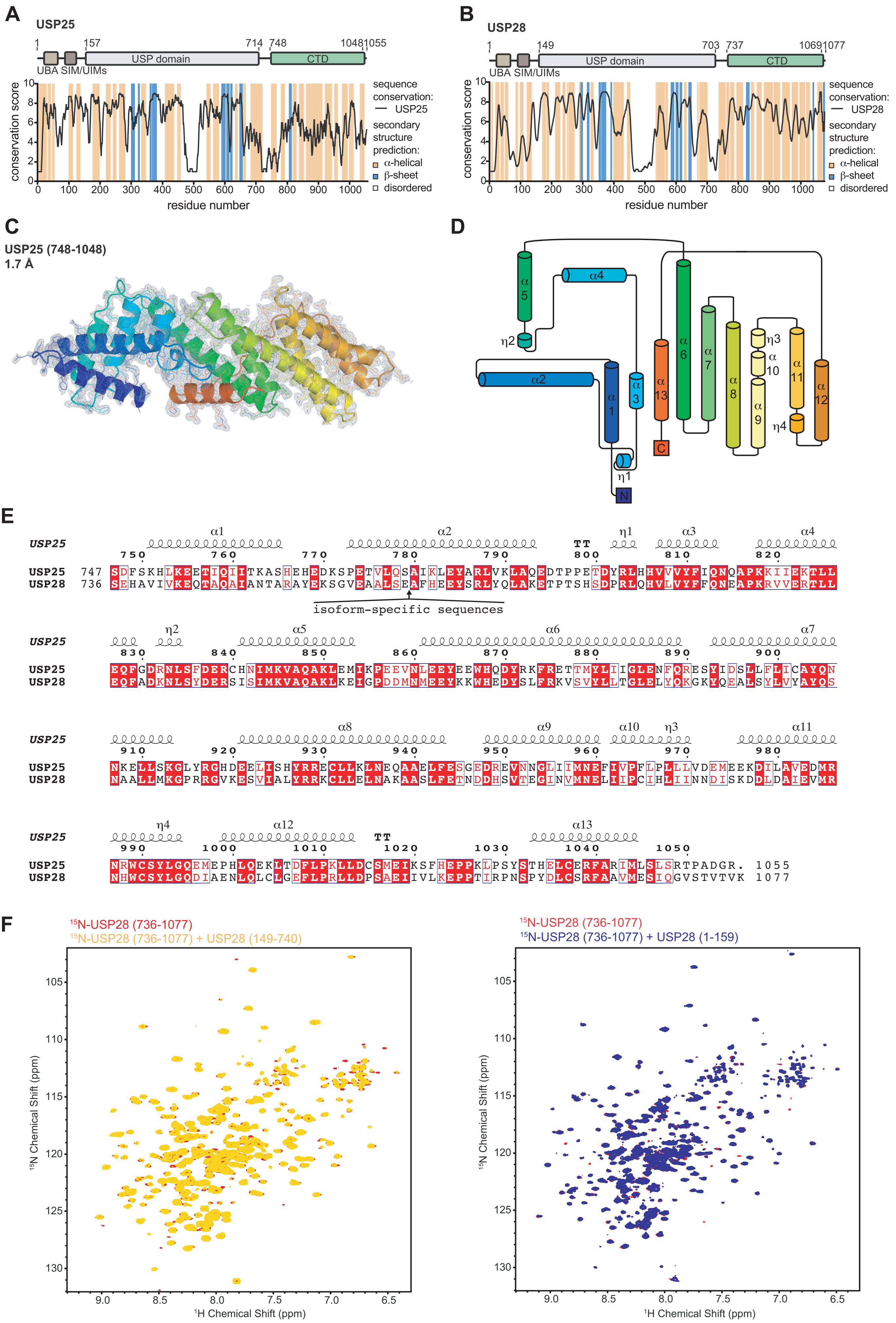
A
USP25
1 157 714 748 1048 1055
USP domain
CTD
UBA SIM/UIMs
conservation score
residue number
sequence conservation:
USP25
secondary structure prediction:
α-helical
β-sheet
disordered
B
USP28
1 149 703 737 1069 1077
USP domain
CTD
UBA SIM/UIMs
conservation score
residue number
sequence conservation:
USP28
secondary structure prediction:
α-helical
β-sheet
disordered
C
USP25 (748-1048)
1.7 Å
D
N
C
E
USP25 747 SDFSKHLKEETIQIITKASHEHEDKSPETVLQSAIKLEYARLVKLAQEDTPPETDYRLHHVVVYFIQNQAPKKIIEKTLL
USP28 736 SEHAVIVKEQTAQAIANTARAYEKSGVEAALSEAFHEEYSRLYQLAKETPTSHSDPRLQHVLVYFFQNEAPKRVVERTLL
isoform-specific sequences
USP25 EQFGDRNLSFDERCHNIMKVAQAKLEMIKPEEVNLEEYEEWHQDYRKFRETTMYLIIGLENFQRESYIDSLLFLICAYQN
USP28 EQFADKNLSYDERSISIMKVAQAKLKEIGPDDMNMEEYKKWHEDYSLFRKVSVYLLTGLELYQKGKYQEALSYLVYAYQS
USP25 NKELLSKGLYRGHDEELISHYRRECLLKLNEQAAELFESGEDREVNNGLIIMNEFIVPFLPLLLVDEMEEKDILAVEDMR
USP28 NAALLMKGPRRGVKESVIALYRRKCLLELNAKAASLFETNDDHSVTEGINVMNELIIPCIHLIINNDISKDDLDAIEVMR
USP25 NRWCSYLGQEMEPHLQEKLTDFLPKLLDCSMEIKSFHEPPKLPSYSTHELCERFARIMLSLSRTPADGR. 1055
USP28 NHWCSYLGQDIAENLQLCLGEFLPRLLDPSAEIIVLKEPPTIRPNSPYDLCSRFAAVMESIQGVSTVTVK 1077
F
15N-USP28 (736-1077)
15N-USP28 (736-1077) + USP28 (149-740)
15N-USP28 (736-1077)
15N-USP28 (736-1077) + USP28 (1-159)
15N Chemical Shift (ppm)
1H Chemical Shift (ppm)

Figure S5. Structural analysis of the C-terminal domains of USP25 and USP28. Related to Figure 5.

(A) Sequence conservation of USP25 plotted as a rolling window average in black of scores obtained from the ConSurf webserver. Secondary structure prediction of human USP25 from the Concord webserver indicated as background color. Schematic representation of the domain architecture of USP25 above the graph including the newly identified C-terminal domain.

(B) Analysis as in (A) for USP28.

(C) Overall view of the asymmetric unit of the crystal structures of USP25 (748-1048). Residues are shown as lines and in cartoon representation. The overlaid electron density map in grey corresponds to weighted $2|F_O|–|F_C|$ electron density contoured at 1 σ.

(D) Topology diagram of the USP25 (748-1048) structure generated with the Pro-origami webserver. α helices and 3_{10} helices (η) are consecutively numbered.

(E) Sequence alignment of the C-terminal regions of human USP25 and USP28 with secondary structure elements indicated according to the USP25 (748-1048) structure. The insertion point where sequences of varying length are added to both USP25 and USP28 in isoforms lies within α2 of the CUEL domain. These sequences at least for USP25 customize the function of the DUB by facilitating different protein-protein interactions (Bosch-Comas et al., 2006). With large parts of the inserted sequences being predicted to be disordered, it is unknown how these would structurally remodel the C-terminal region of USP25 and USP28.

(F) ^{1}H-^{15}N BEST-TROSY NMR spectra showing the ^{15}N-labeled C-terminal domain of USP28 (736-1077) (red) overlaid with ^{15}N-labeled USP28 (736-1077) in the presence of unlabeled USP28 (149-740) (yellow, *left*) or unlabeled USP28 (1-159) (blue, *right*). Labeled proteins were at 75 µM and unlabeled proteins were added to equimolar amounts.

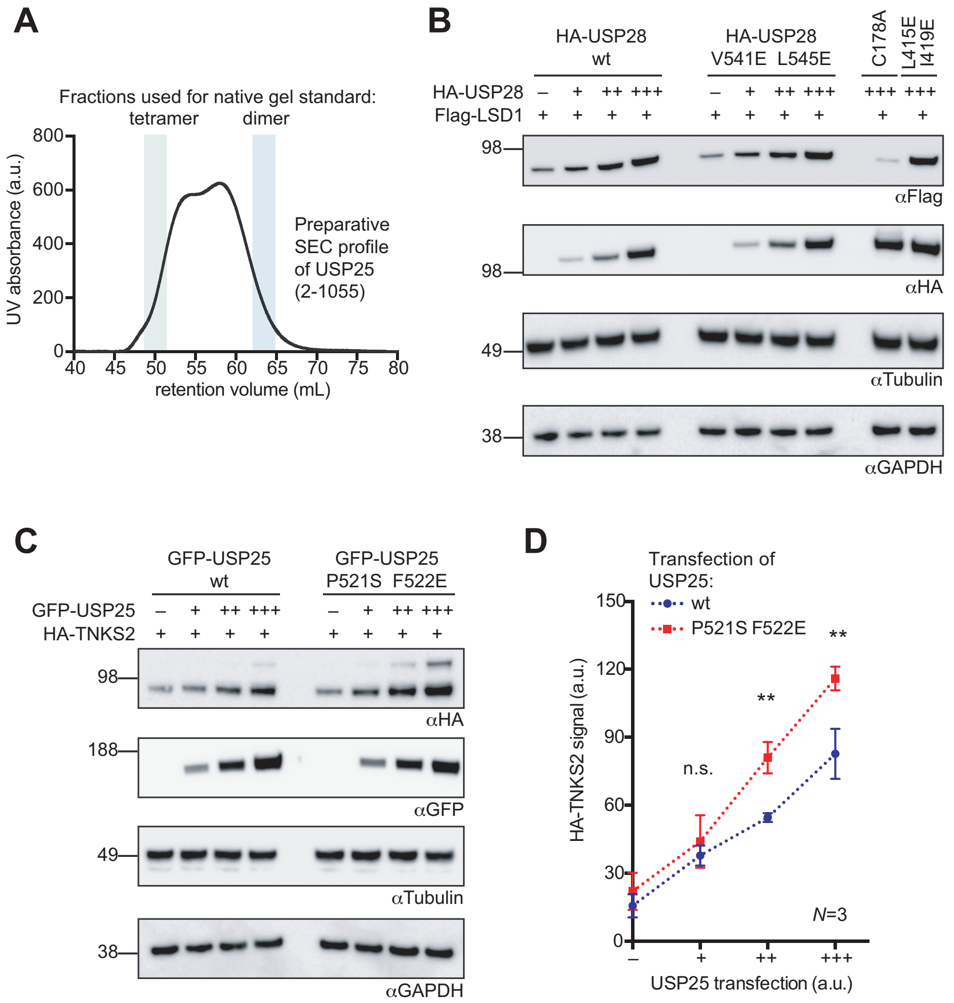
A
Fractions used for native gel standard:
tetramer
dimer
UV absorbance (a.u.)
800
600
400
200
0
40 45 50 55 60 65 70 75 80
retention volume (mL)
Preparative SEC profile of USP25 (2-1055)
B
HA-USP28 wt
HA-USP28 V541E L545E
C178A
L415E I419E
HA-USP28 – + ++ +++ – + ++ +++ +++ +++
Flag-LSD1 + + + + + + + + + +
98
αFlag
98
αHA
49
αTubulin
38
αGAPDH
C
GFP-USP25 wt
GFP-USP25 P521S F522E
GFP-USP25 – + ++ +++ – + ++ +++
HA-TNKS2 + + + + + + + +
98
αHA
188
αGFP
49
αTubulin
38
αGAPDH
D
Transfection of USP25:
wt
P521S F522E
HA-TNKS2 signal (a.u.)
150
120
90
60
30
0
n.s.
**
**
N=3
– + ++ +++
USP25 transfection (a.u.)

Figure S6. Functional assessment of oligomerization states of USP25 and USP28. Related to Figure 6.

(A) Size-exclusion chromatogram of full-length human USP25. Fractions used as size standards for native PAGE analysis are indicated with colors.

(B) Immunoblotting for indicated epitopes in lysates from HEK-293 cells transfected with vectors for the expression of Flag-LSD1 and increasing amounts of vector for HA-tagged USP28 proteins.

(C) Experiment as in B with HA-TNKS2 as substrate and increasing amounts of vector of GFP-tagged USP25 constructs. Since transfection of identical vector amounts of USP25 (P521S F522E) and USP25 wt led to a larger amount of protein of USP25 (P521S F522E), presumably due to its higher activity and increased auto-deubiquitination, the amounts of transfected vector were adjusted to achieve similar cellular protein concentrations.

(D) Quantification of the HA-TNKS2 immunoblotting signal from three independent repeats of the experiment shown in C. Data are shown as mean ± standard deviation (**: $p < 0.01$, two-tailed *t*-test; n.s., not significant).

Supplemental Figure 7

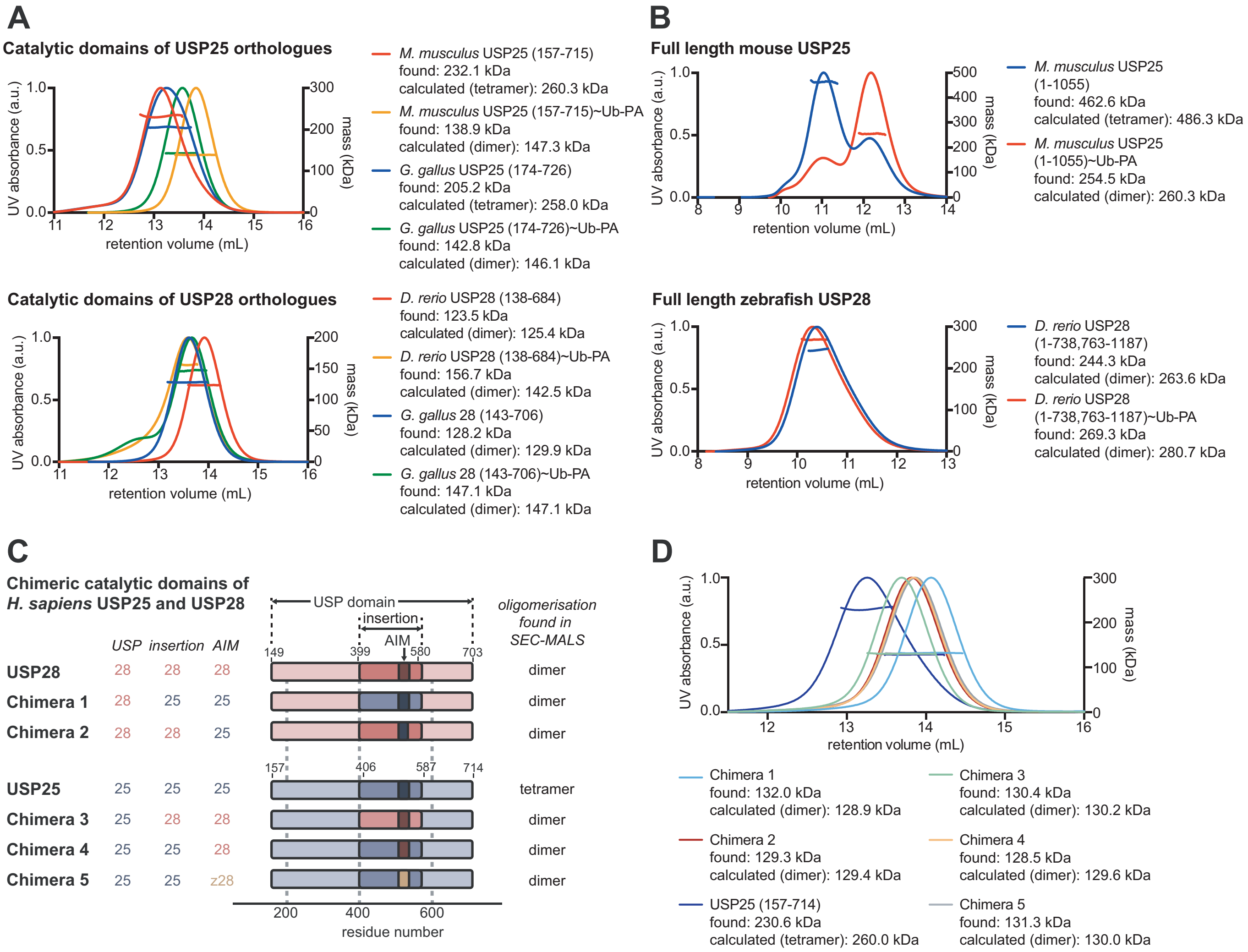

Figure S7. Molecular requirements for distinct oligomerization states of USP28 and USP25 catalytic domains. Related to Figure 7.

(A) SEC-MALS analysis of catalytic domains of USP28 orthologues (*top*) and catalytic domains of USP25 orthologues (*bottom*) both in their apo form and bound to Ub-PA. Identified masses are matched to either tetrameric or dimeric expected masses.

(B) SEC-MALS analysis of full length mouse USP25 (*top*) and full-length zebrafish USP28 (*bottom*) both in their apo form and bound to Ub-PA. Identified masses are matched to either tetrameric or dimeric expected masses.

(C) Schematic representation of chimeric catalytic domain constructs of human USP25 and USP28 proteins. Grafting of sequences was performed as indicated, based on equivalent positions identified from a sequence alignment and from superposition of structures (insertion of USP28: Y399-R580, "AIM"-equivalent sequence: S508-Q527; insertion of USP25: H406-I587, AIM: 515-534). In chimera 5, the AIM of human USP25 was replaced with the equivalent sequence of *D. rerio* USP28. The zebrafish USP28 sequence shares the central IHKPFTQ motif with the AIM of human USP25 (see Figure 4B), but contains different amino acids flanking this motif on both sides. These changes are sufficient to prevent tetramerization (see SEC-MALS data in B and D).

(D) SEC-MALS analysis of chimeric catalytic domain constructs shown in C and tetrameric USP25 as control. Identified masses are matched to either tetrameric or dimeric expected masses. Observed oligomerization states are listed in C.

Uncropped blots and gels Supplemental Figure 8

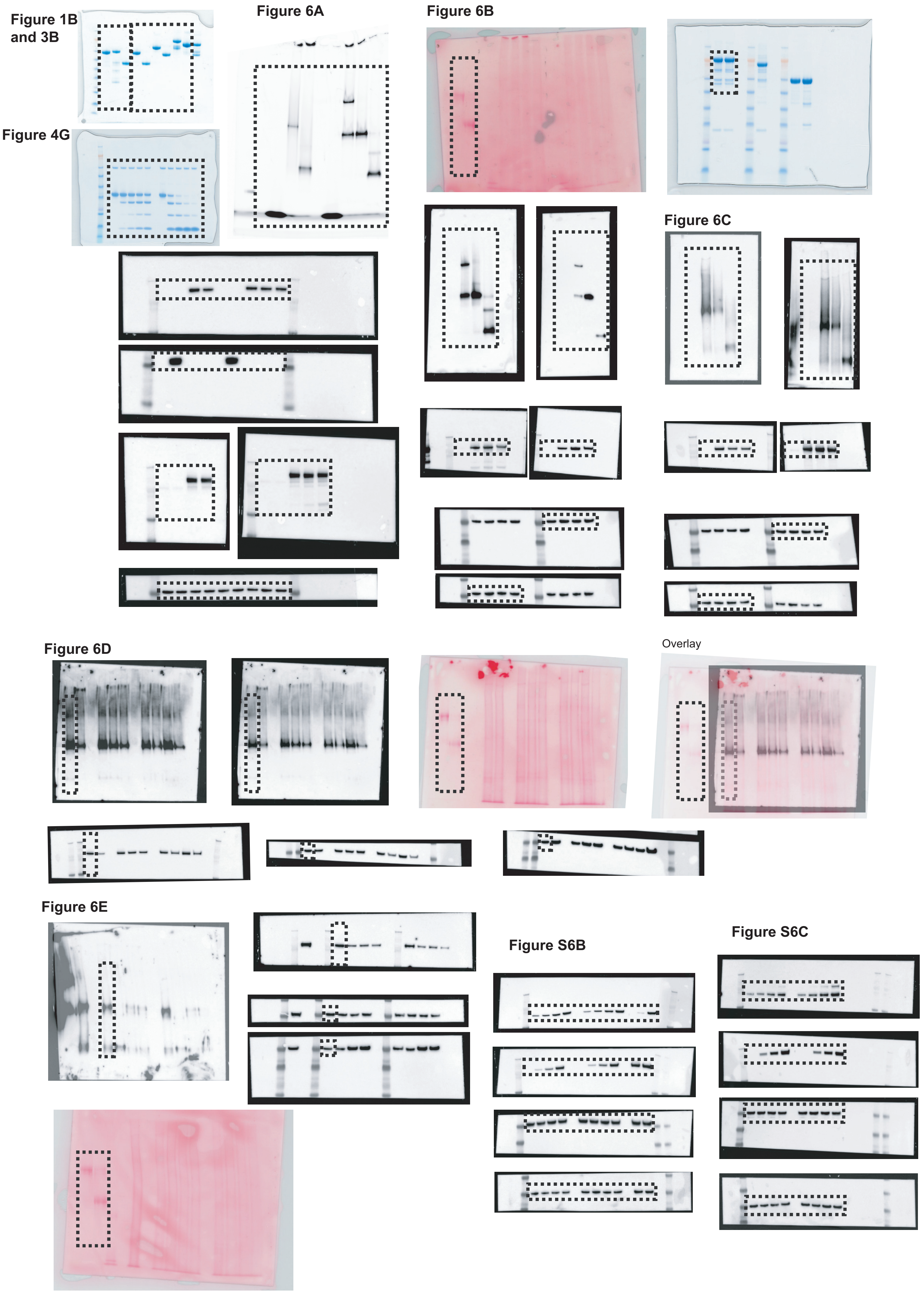

Figure S8. Uncropped gels and blots. Related to Figures 1, 3, 4, 6 and S6.

Supplemental Table 1. Parameters derived from SAXS data analysis. R_g denotes the radius of gyration obtained from Guinier plot fitting, D_{max} and R_{ave} denote the maximum distance and the average radius, respectively, obtained from real space data analysis. Related to Figures 2A, 3C and S3A.

**Sample**	**R_g (Å)**	**D_{max} (Å)**	**R_{ave} (Å)**	**Peaks in *P(r)* (Å)**
USP28 149 -703 apo	50.6	145	65.1	33.8, 79.0
USP28 149-703~Ub-PA	51.4	146	64.3	35.3, 76.6
USP28 149-Δinsert-703 apo	24.1	73	30.9	30.8
USP28 149-Δinsert-703~Ub-PA	24.1	73	31.5	30.8
USP25 157-720 apo	45.8	130	60.5	61.8
USP25 157-720~Ub-PA	46.6	138	60.3	34.0, 73.8
USP25 157-Δinsert-720 apo	24.2	73	31.0	30.0
USP25 157-Δinsert-720~Ub-PA	24.9	73	31.8	30.4
